# Molecular Dynamics of Ionic Liquids from Fast-Field Cycling NMR and Molecular Dynamics Simulations

**DOI:** 10.1021/acs.jpcb.2c01372

**Published:** 2022-09-12

**Authors:** Julian B. B. Beckmann, Daniel Rauber, Frederik Philippi, Kateryna Goloviznina, Jordan A. Ward-Williams, Andy J. Sederman, Mick D. Mantle, Agílio Pádua, Christopher W. M. Kay, Tom Welton, Lynn F. Gladden

**Affiliations:** † Department of Chemical Engineering and Biotechnology, University of Cambridge, Philippa Fawcett Drive, Cambridge CB3 0AS, United Kingdom; ‡ Department of Chemistry, Saarland University, Campus B2.2, 66123 Saarbrücken, Germany; § Department of Chemistry, Molecular Sciences Research Hub, Imperial College London, White City Campus, London W12 0BZ, United Kingdom; ∥ Laboratoire de Chimie, École Normale Supérieure de Lyon & CNRS, 69364 Lyon, France; ⊥ London Centre for Nanotechnology, University College London, 17-19 Gordon Street, London WC1H 0AH, United Kingdom

## Abstract

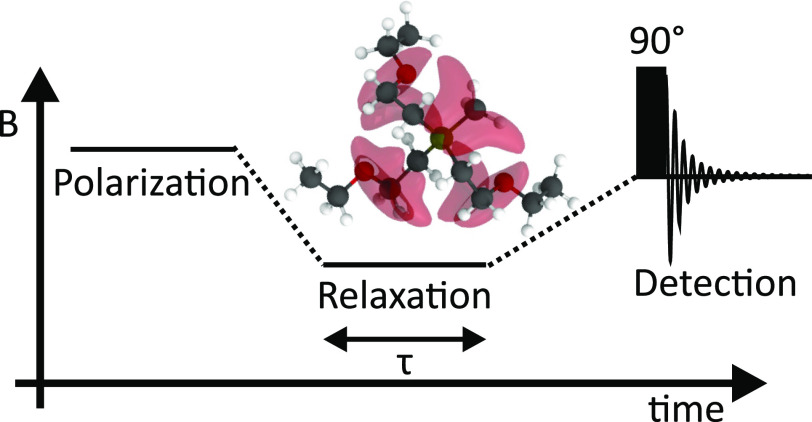

Understanding the connection between the molecular structure of ionic liquids and their properties is of paramount importance for practical applications. However, this connection can only be established if a broad range of physicochemical properties on different length and time scales is already available. Even then, the interpretation of the results often remains ambiguous due to the natural limits of experimental approaches. Here we use fast-field cycling (FFC) to access both translational and rotational dynamics of ionic liquids. These combined with a comprehensive physicochemical characterization and MD simulations provide a toolkit to give insight into the mechanisms of molecular mechanics. The FFC results are consistent with the computer simulation and conventional physicochemical approaches. We show that curling of the side chains around the positively charged cationic core is essential for the properties of ether-functionalized ionic liquids, and we demonstrate that neither geometry nor polarity alone are sufficient to explain the macroscopic properties.

## Introduction

Ionic liquids are a class of liquid materials with a wide range of current and potential applications, resulting from their unique combination of properties, namely, the features of both molecular liquids and conventional molten salts. However, ionic liquids also suffer from inherent disadvantages compared to molecular liquids which hamper their wider use. In particular, the dynamics of ionic liquids are in general much slower than those of molecular liquids. This is of special concern in the field of electrochemistry, where electrochemical devices, such as rechargeable batteries^1^ and supercapacitors,^2^ are limited in their charging and discharging rates when ionic liquids are used in a pure, undiluted state.

Key for the successful utilization of ionic liquids is detailed knowledge about how their properties emerge from the molecular structure. Only then will it be possible to fulfill the promise of ionic liquids as “designer solvents”. However, variations in the molecular structure often effect changes in more than one parameter at a time. In particular, flexibility and functionalization are not independent. It is, therefore, desirable to limit the number of varied parameters where possible, an approach we will refer to as targeted modification.^3^

Beyond the practical aspects of optimizing ionic liquid transport properties, there are still many controversies about the interrelation between molecular motion and macroscopic properties. For instance, little is known about the coupling between rotational and translational motion of the ions and how this relates to the liquid structure. Nevertheless, it is well-established that ionic liquids show structural and dynamical heterogeneities, which can be interpreted as a signature of ion caging.^4^ The cage dynamics^5^ of ions have been reported to be responsible for important experimental observations such as the breakdown of the Stokes–Einstein relation^6,7^ and subdiffusive behavior on short time scales.^8,9^ The lifetime of the ions inside the cage of ions of opposite charge was reported to be the rate-determining step for the ionic liquid dynamics.^5^ Furthermore, ionic liquids show structural relaxation that obey the Vogel–Fulcher–Tammann (VFT) behavior instead of an Arrhenius-type *T*-dependence, similar to other fragile glass-forming materials.^10^

Fast-field cycling (FFC) is an NMR method that allows the longitudinal relaxation to be probed across a Larmor frequency range of 4 orders of magnitude.^11,12^ The benefit of FFC compared to more established relaxation measurement techniques at fixed magnetic fields such as inversion or saturation recovery is the ability to study the magnetic field strength dependency of the relaxation process, the so-called NMR dispersion (NMRD). Due to the close interconnection of the relaxation process with molecular dynamics, NMRD data in combination with theoretical relaxation models can be a rich source of dynamic information. For instance, FFC experiments can be used to probe rotational and translational movements simultaneously,^11−13^ thereby providing additional information that cannot be obtained using pulse-field gradient NMR, which measures translational motion.^14^ Thus, the application of FFC to ionic liquids can provide unique insights regarding rotational and translational dynamics.^15−18^

Computer simulation, in particular, classical atomistic molecular dynamics (MD), provides a tool to connect molecular structure with macroscopic properties. The idea is to choose a force field (i.e., an effective Hamiltonian) that emulates the effects of the key interactions in the system, without having to rely on computationally expensive first-principles calculations. MD can give insights to how macroscopic properties arise on a collective level in the many cases where this is not obvious from the force field itself. A key advantage of MD simulations is that the force field can be altered deliberately in ways that would not be feasible experimentally. Hence, key components of the molecular interactions can be identified, provided that the object of study is well-represented in the first place. Common examples are MD simulations in which the barrier for rotation around a particular bond is restricted, thus allowing differences to the native simulation to be evaluated.^19−23^ MD simulations are thus prime candidates for targeted modifications, far beyond what is experimentally possible.

**Figure 1:**
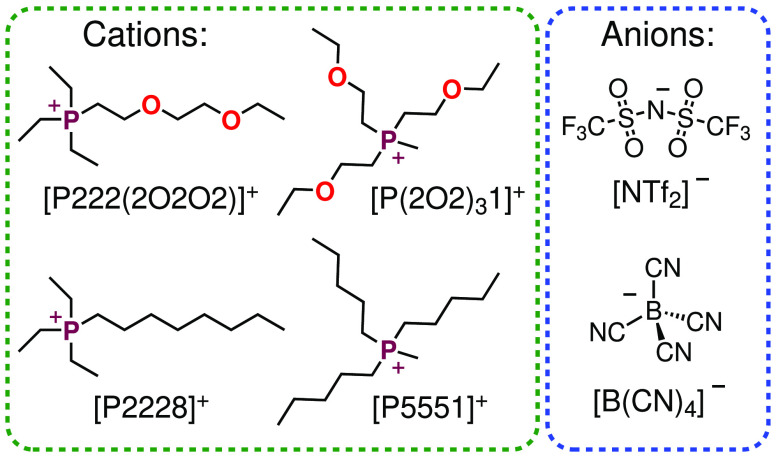
Molecular structures and abbreviations of the ionic liquid cations and anions used in this work. The numbers in the cation nomenclature refer to the number of carbon atoms in the hydrocarbon segments.

Here, we investigated ionic liquids based on quarternary phosphonium cations with pure alkyl side chains and analogues of same chain length but with ether substitutions in the side chain. The chemical structures and nomenclature of the ionic liquids are given in Figure 1. The numbers in the cation nomenclature refer to the number of C atoms in the alkyl chains (2: ethyl; 5: pentyl; 8: octyl) and the ether side groups (2O2: 2-ethoxy-ethyl; 2O2O2: 2-(2-ethoxy–ethoxy)ethyl). We measured the transport properties on macroscopic characteristics of interest (viscosity and molar conductivity) to correlate these with the dynamics of the ions on the molecular scale. As model systems, we chose the triethyl octyl phosphonium cation [P2228]^+^, which has three short and a long side chain, as well as the cation methyl tripentyl phosphonium [P5551]^+^, consisting of a short methyl group and three hydrocarbon chains of medium length. These cations were selected to investigate the effect of different cation structures, while keeping the molar mass approximately constant. The results were compared to the cation [P222(2O2O2)]^+^ where the methylene groups in the γ- and ζ-position are replaced by an ether group and the  bearing ether functionalities in all three γ-positions of the side groups. These cations were chosen as large differences in the dynamic properties between ionic liquids cations with purely hydrocarbon side chains and those with ether functionalities have been observed previously.^24,25^ The explanation for the accelerated dynamics of the ether substituted ionic liquids was suggested to be an altered cation conformation where the ether chain curls around the positively charged cation center.^26,27^ Such curled cations have contracted structures compared to the linear, alkylated analogues, a more pronounced shielding of the cation charge and a higher degree of freedom for anion coordination around the cation. These combined effects lead to overall faster dynamics of the ether ionic liquids and are more pronounced for multiple ether substitution in the side chains.^25^ Each of these cations was combined with bis(trifluoromethylsulfonyl)imide  and the tetracyanoborate  anions. The anions were chosen to obtain insight into the possible contributions of geometry and conformational flexibility (the nonspherical, flexible  anion showing a dynamic equilibrium between cis and trans conformers, and the rigid, spherical ). From an NMR point of view, the  anion has the benefit that due to the low natural abundance of ^15^N and ^13^C, only negligible amounts of  nuclei are contained in the anions, which facilitates the theoretical description of the relaxation process. However, due to its importance in ionic liquid research, we also conducted NMR relaxation experiments of  containing ionic liquids despite their more theoretically challenging relaxation behavior due to the occurrence of ^19^F in the anion.

Using ^1^H FFC, we have investigated the NMR dispersion of the ionic liquids presented in Figure 1 for at least five different temperatures, depending on the sample, and in an overall Larmor frequency range from 10 kHz to 40 MHz. Subsequent modeling of the resulting NMRD profiles facilitated the estimation of rotational and translational correlation times.

## Theory

### Relaxation Theory

The most prominent and versatile approach for a theoretical description of a FFC experiment is the Redfield perturbation theory approach.^12,13,28^ For bulk liquids, the secular part of the dipolar Hamiltonian averages to zero and relaxation is determined solely by the weak nonsecular dipolar interactions, which do not vanish under motional averaging.^11,13,28^ Thus, the Redfield condition can be expected to be fulfilled for the FFC experiments in this study.^13,28,29^ Considering that no paramagnetic substances are present in the investigated samples, the so-called like spin case of the Redfield theory applies for dipolar coupling between ^1^H-spins and the longitudinal relaxation rate *R*_1_, the inverse of the spin–lattice relaxation time *T*_1_, is given by the following expression:^11−13,15,28,30^
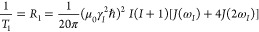
1Here, μ_0_ is the vacuum permeability, γ_*I*_ is the gyromagnetic ratio of spins of the type *I*, *ℏ* is the reduced Planck constant, *I* is the spin quantum number of the investigated spins, *J* is the spectral density, and ω_*I*_ is the Larmor frequency of the spins *I* at the relaxation field strength. The spectral density is a Larmor frequency-dependent function, which describes the relaxation governing dynamic behavior of the sample and is formally defined as the cosine Fourier transform of a correlation function *G*(*t*):^11−13,22^
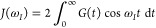
2It should be mentioned that the correlation function *G*(*t*) is not the same as the rotational correlation function *C*_rot_(*t*) which is used for the MD simulations and given by eq 14. Due to the complexity of molecular dynamics, it is usually not possible to define a correlation function which can sufficiently describe all relevant modes of motion in a sample, but it is possible to separate different types of motion from each other. This means the overall relaxation rate *R*_1,total_ can be written as a superposition of an intra- and an intermolecular part.
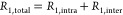
3where *R*_1,intra_ and *R*_1,inter_ are given by eq 1. Consequently, the next and last step for defining the relaxation model of the investigated ionic liquids, is to determine the spectral density function *J*_intra_ and *J*_inter_. In the intramolecular case, a Cole–Davidson function can be used as a phenomenological description of the relaxation contribution due to rotational movement of intramolecular spins in a single molecule. Hence, *J*_intra_ is given by the following expression:^12,15,30^
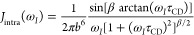
4with
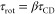
5here *b* is the average intramolecular spin–spin-distance given by eq 8, β is a phenomenological stretching parameter with 0 < β ≤ 1, and τ_rot_ is the rotational correlation time. Estimating *J*_inter_ is considered to be more difficult, because the intermolecular relaxation is originating from the translational as well as the rotational diffusion of dipolar coupled spins in different molecules. However, in a phenomenological model the full rotational contribution can be described by eq 4, and *J*_inter_ can be approximated through the so-called hard sphere model, which is defined through the following equation:^15,30,31^
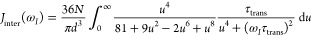
6with
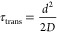
7where *N* is the spin-density, *d* is the average intermolecular spin–spin-distance given by eq 9, *u* is an arbitrary integration variable, τ_trans_ the translational correlation time, and *D* is the self-diffusion coefficient. Here, it should be mentioned that the intermolecular distance *d* is not equivalent to the hydrodynamic diameter.

The intramolecular distances *b* and intermolecular distances *d* between spins can be calculated using eqs 8 and 9, respectively. Here, *r*_*ij*_ is the distance between the pair of spins *i* and *j*. The sums run over all intramolecular (eq 8) or over all intermolecular (eq 9) pairs of spins. The averages defined by these equations can be calculated with ease from the atomistic information in an MD trajectory.
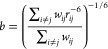
8
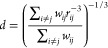
9

The weighting factors *w*_*ij*_ are commonly chosen as 1.^32^ However, with this choice, the calculated average distances diverge with increasing cutoff. Thus, for the sake of simplicity, we chose an exponential weighting function, .

### Classical Molecular Dynamics

An important aspect of MD simulations is the choice of the force field. A carefully designed MD simulation usually gives results which are “exact” for the force field. It is thus critical to avoid a biased force field or, at best, be aware and wary of the biases. Considerable progress has been made in recent years on force field development and MD simulations for ionic liquids. In particular, the CL&P force field, which we will use as a basis for our own simulations, has seen widespread use.^33−39^ The mathematical representation of the interactions is that of the OPLS potential.^40−42^ The potential energy is divided into nonbonded and bonded interactions:
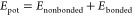
10

The nonbonded interactions are calculated for all pairs of atomic sites *i* and *j* separated by a distance *r*_*ij*_. The 12/6 Lennard-Jones potential defined by the parameters ϵ (well depth) and σ (contact distance) accounts for van der Waals interactions, whereas the Coulomb potential defined by the atomic charges *q* accounts for electrostatic interactions:
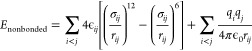
11

Bonded interactions are separated into bond, angle, and dihedral contributions. Bond and angle contributions are approximated with harmonic potentials with a spring constant *k* and the equilibrium bond distance *r*_0_ or angle ϕ_0_, respectively. The dihedral contributions (torsions) are obtained from a truncated Fourier series:
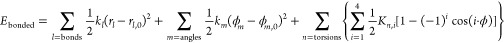
12

Nonbonded interactions are zeroed for pairs of atoms separated by one or two bonds, hence bond and angle contributions can be determined directly by fitting an *ab initio* potential energy scan with the empiric harmonic potential. In contrast, the contribution of nonbonded interactions between atomic sites separated by three and more bonds is important for the dihedral contributions. Thus, the nonbonded interactions separated by exactly three bonds are weighted with 0.5, and the dihedral contributions have to be obtained by fitting the truncated Fourier series to the difference between the *ab initio* potential energy surface and the force field energy with zeroed dihedral contributions. At this point, we will introduce a targeted modification. Apart from the force field where the P–C–C–X dihedral angles are fitted to their native potential energy surfaces, which produce a curled (X = O) or linear (X = CH_2_) conformation, we will also fit the P–C–C–X dihedral angles to the respective other potential energy surface. The curled conformation is the one with the oxygen atom of the ether side chain pointing toward the positively charged phosphonium core; see the Supporting Information. Thus, we can directly investigate the effect of the curling, without any changes to the rest of the force field.

Critically, this force field does not include explicit treatment of polarizability, which is important for accurate simulations of ionic liquids.^43−51^ However, polarizable simulations are computationally more expensive than nonpolarizable ones and come with their own pitfalls and potential biases. Thus, we carried out additional polarizable simulations for only a few selected systems which we wanted to study in detail. In these cases, we make use of the recently developed CL&Pol force field with temperature-grouped Nosé–Hoover thermostat.^43,52^

Translational and rotational dynamics for comparison with the fast-field cycling approach are readily available from a molecular dynamics trajectory. Diffusion coefficients can be calculated from the ensemble average of the mean squared displacement.^53^ The displacement Δ*r*(*t*) is the distance covered by the center of mass of a cation or anion over the time *t*:
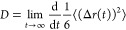
13

The rotational dynamics are obtained by tracking the orientational evolution of a characteristic vector in the molecular ion. In this work, we will use the vector from the phosphorus atom to the center of mass of the methyl group. The angle θ(*t*) swept by the vector in the time *t* allows the calculation of a rotational autocorrelation function *C*_rot_(*t*). The second Legendre polynomial is used here to allow us to compare τ_rot_ from the MD simulation (eq 14) with the experimental determination of τ_rot_ from FFC (eq 5).^16^ The integral of the autocorrelation function gives τ_rot_:
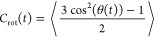
14

Here, *C*_rot_(*t*) is a simplified correlation function that probes the evolution of only one specific vector in the molecule, representative for the rotational dynamics of the whole molecule. However, in the case of anisotropic rotation this simplified model can substantially differ from the rotational contribution of the correlation function *G*(*t*), which is probed during the fast-field cycling experiment. The direct computation of *G*(*t*) from the MD simulation is challenging and will be the subject of future studies.^32,54^

## Materials and Methods

Details on the synthesis of the ionic liquids and corresponding analytical data are given in the Supporting Information. Halide residues in the ionic liquids could not be detected by ion chromatography. Details regarding the temperature-dependence of the macroscopic properties (experimental values of density, viscosity, and conductivity and fitting data) and the molecular dynamics simulations can also be found in the Supporting Information.

### Physicochemical Properties

The physicochemical properties were determined as reported in the literature.^24,55^ Prior to each measurement the samples were dried for at least 2 days in high vacuum and further stored and handled using Schlenk techniques or a Labmaster 130 glovebox (MBraun, Garching, Germany). The viscosity η was determined using a MCR 301 Rheometer (Anton Paar, Graz, Austria) equipped with an CP50–1 cone of 49.95 mm diameter and cone angle 1° using 0.101 mm gap size. For each temperature 30 shear rates ranging from 5 to 80 s^–1^ were recorded in linear steps (averaging data values over 15 s per shear rate) after sufficiently long temperature equilibration. The liquids were characterized by Newtonian behavior. The conductivity was measured using a commercial conductivity probe consisting of two rectangular platinized platinum electrodes fused in glass with a nominal cell constant of 0.5 cm^–1^ (WTW, Weilheim, Germany) and a SP-150 potentiostat (BioLogic, Seysinnet-Pariset, France). The actual cell constant was determined using commercial conductivity standards. Impedance measurements were conducted at each temperature controlled by a Proline RP 1845 thermostat using amplitudes of 5, 10, and 15 mV and 50 different frequencies ranging from 200 kHz to 1 Hz in logarithmic steps. The molar conductivity Λ_M_ was calculated from the density ρ, the specific conductivity κ, and the molar mass *M*:
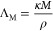
15

Temperature stability during the experiments with the thermostat was approximately ±0.01 and ±0.002 °C for the rheometer. The estimated error for the viscosity and conductivity values obtained are ±1.5 and ±2% respectively, as determined by repeated measurements of commercial standards.

### FFC Experiments

All ^1^H FFC experiments were conducted using a Stelar Spinmaster Duo relaxometer. Every NMRD profile spans the frequency range 10 kHz to 40 MHz, with measurements taken at 32 logarithmically spaced frequencies. For relaxation fields stronger than 12 MHz, a nonpolarized sequence was used, whereas for weaker magnetic fields a prepolarized sequence was applied. For a single *T*_1_ experiment, 32 logarithmically spaced time delays starting from 1 ms and increasing to 6 times the expected *T*_1_ were used. In all cases, two scans provided a sufficient signal-to-noise ratio. The magnetization decays obtained displayed a monoexponential behavior and *T*_1_ could be extracted from a single exponential fit. The temperature of all samples was controlled via the internal temperature controller of the FFC relaxometer. The temperature controller was calibrated against an external thermocouple, which gave a maximum error of ±1 °C. It was further noticed that the error is increasing with decreasing temperature. Below 22 °C, the temperature was maintained via a nitrogen gas stream generated from a liquid nitrogen boil-off, whereas for temperatures higher than or equal 22 °C a compressed air gas stream was used. To reach thermal equilibrium, the sample was left undisturbed for 30 min after a temperature change. The investigated temperature range was dictated by experimental limitations. The switching time of the relaxometer is 3 ms, which means that relaxation rates significantly higher than 300 Hz are difficult to observe. Furthermore, some ionic liquids started to solidify during the experiment if cooled to lower temperatures. In consequence, the lowest temperature investigated was selected in order to reach relaxation rates that do not exceed the experimental limit of the relaxometer and to prevent freezing of the sample. In contrast, the upper temperature limit was chosen so as to reduce instrumental stress on the relaxometer due to long polarization times. This means that only temperatures, which ensured relaxation rates considerably faster than 2 Hz were considered in this study.

### FFC Fitting

All NMRD profiles were fitted in *Matlab* with the *lsqcurvefit* function which utilizes a nonlinear least-squares solver in combination with a trust-region-reflective algorithm. The upper and lower bounds of the fitting parameters were chosen to cover the maximum range of physically sensible values. To test for numerical stability, the starting values of the fitting parameters were randomly changed in the interval defined by the lower and upper bounds. To ensure a high numerical stability, the number of fitting parameters was reduced to a minimum. This means that only the correlation times τ_trans_ and τ_rot_ were obtained via fits of the experimental FFC dispersion curves. The intra- and intermolecular spin distances *b* and *d* were obtained from the MD simulations (table S23) as outlined earlier and used as fixed parameters in the fitting of the FFC dispersion curves to ensure numerical stability. The spin-density *N* per unit volume is given by , where *n*_S_ is the number of spins per molecule, ρ is the density, and *M* is the molar mass and can be calculated from the experimentally obtained density values. Furthermore, due to its lack of physical insight and its limited range by definition, the stretching parameter β was not fitted and instead a sensitivity analysis was deployed. Hence, β was varied in increments of 10^–2^ in the range of 0–1 and the value of β, which minimized the least-squares residuals was chosen. The obtained β-values can be found in the Supporting Information.

## Results

### Physicochemical Properties

**Figure 2:**
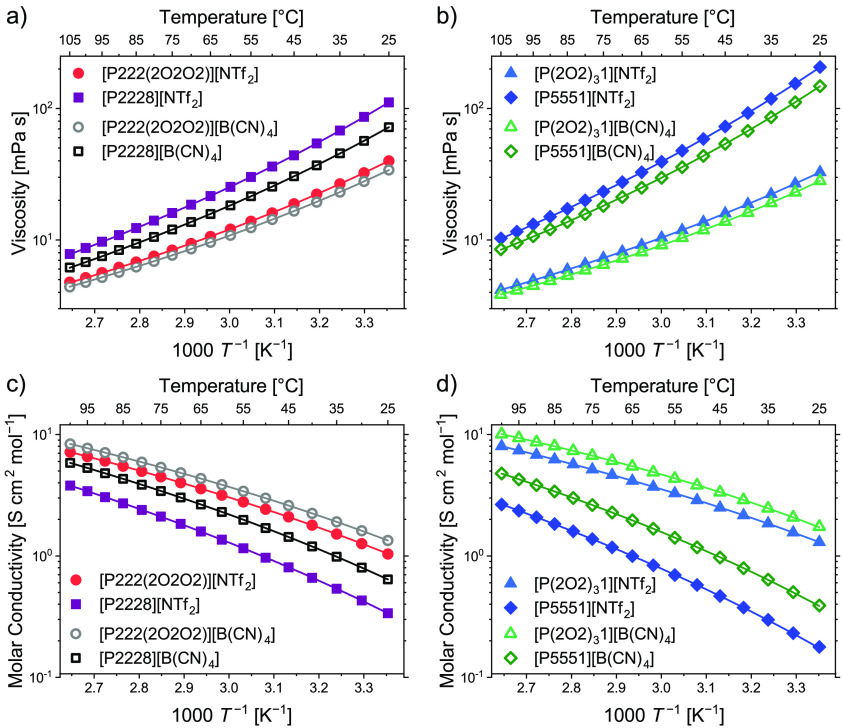
Temperature-dependence of the transport properties of the ionic liquids studied. Data for viscosity (a, b) and molar conductivity (c, d) are shown. Solid lines are the corresponding fits to the VFT equation (eq 16).

As shown in Figure 2, all viscosities of the ether substituted ionic liquids are significantly lower than for the alkylated samples. This lowering of the viscosity is more pronounced for the samples with the  anion and for the cations with the three pentyl groups. The  samples have lower viscosities than the [P222(2O2O2)]^+^ samples for both anions. For the 4-fold alkylated samples the situation is reversed, so higher viscosities are found for the ionic liquids with the [P5551]^+^ cations compared to those with the [P2228]^+^. The comparison of samples with the same cation reveals a lower viscosity for all samples with the  anion. At 25 °C, the ratio between the viscosities of [P2228]^+^ to [P222(2O2O2)]^+^ is only 2.1 for the  anion, whereas it reaches 2.8 for the [NTf_2_]^−^ anion. Correspondingly, for the ratio of [P5551]^+^ to , the values of 5.2 for the  and 5.5 for the  anion are found. The trends for the molar conductivity are similar to the ones observed for the viscosity. Accordingly, higher molar conductivities are found upon ether substitution for samples with the same anion as well as for samples with the  anion and a common cation. Again, the samples with the  cation have higher conductivities than the [P222(2O2O2)]^+^ samples with the long, 2-fold substituted ether chain.

Both transport quantities obey the Vogel–Fulcher–Tammann (VFT) equation as generally found for ionic liquids as fragile glass formers:^56^
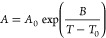
16with *A* being either the viscosity η or molar conductivity Λ_M_. *A*_0_, *B*, and *T*_0_ are material-dependent fitting parameters. The parameter *B* takes positive values for viscosity and negative values for conductivity, consistent with their reciprocal relationship.

To further achieve a more thorough physicochemical characterization, the glass transition temperatures and densities of the ionic liquids were measured. The results including a discussion of the findings and the experimental procedure can be found in the Supporting Information.

### FFC

**Figure 3:**
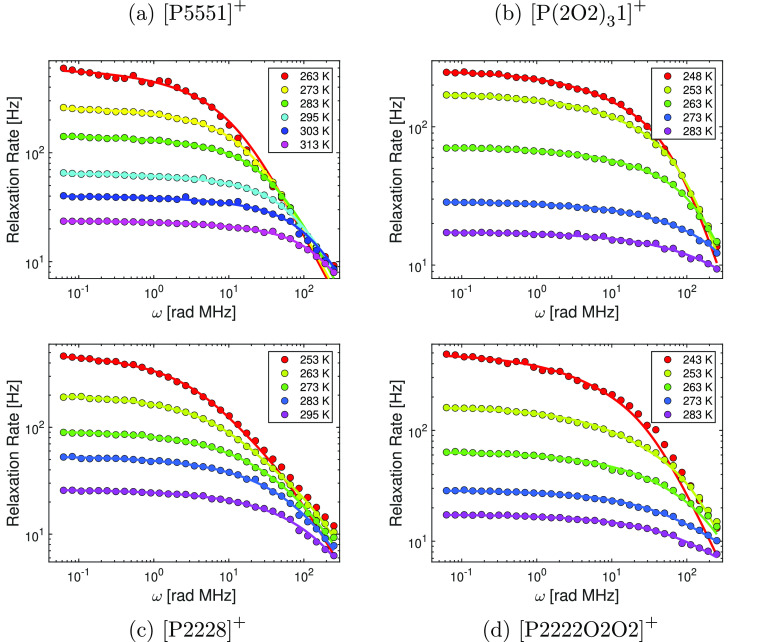
Temperature-dependence of the ^1^H NMRD profiles of the  ionic liquids. The colored circles correspond to the experimental values, whereas the model fit of eqs 1, 3, 4, and 6 is given by the solid lines.

**Figure 4:**
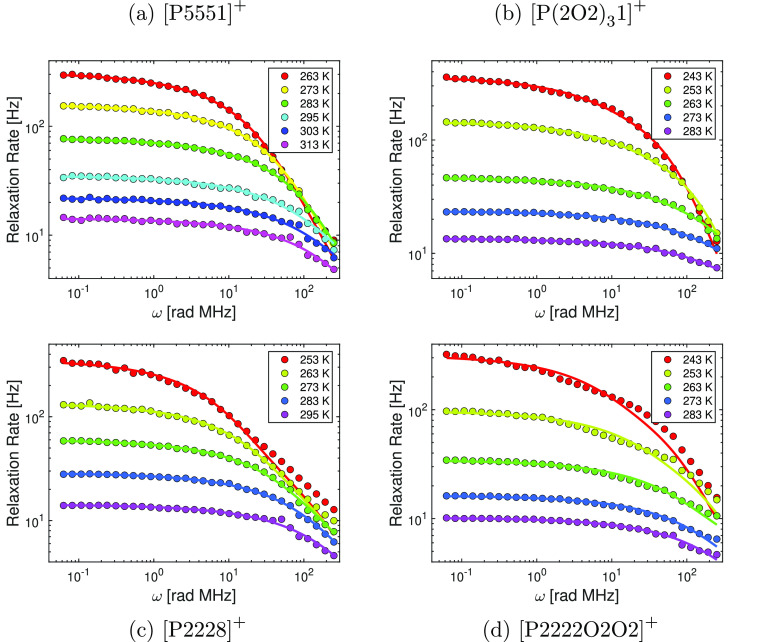
Temperature dependence of the ^1^H NMRD profiles of the  ionic liquids. The colored circles correspond to the experimental values, whereas the model fit of eqs 1, 3, 4, and 6 is given by the solid lines.

Figures 3 and 4 show the temperature-dependent NMR dispersion of the investigated ionic liquids as a function of the relaxation field strength. All NMRD profiles show a reduction in longitudinal relaxation with increasing Larmor frequency. In all cases, an increase in temperature results in slower longitudinal relaxation. Furthermore, the relaxation field dependency of the NMR dispersion is more pronounced at lower temperatures and for each sample only a single relaxation environment could be observed. This means that the magnetization decay of the FFC measurements could be fitted with a single exponential function, and consequently, only one relaxing component is expected to be probed. Comparing the NMRD profiles between the samples, the following trends were identified. Ether-functionalized ionic liquids showed a significant slower relaxation than their alkyl analogs. This effect is independent of the anion and is more significant for the cation pair  than for . Furthermore, a consideration of the effect of the anion shows that the differences in the relaxation behavior between alkyl- and ether-functionalized ionic liquids are more dominant in  ionic liquids. Comparing samples with the same cation but different anions reveals a generally slower relaxation in the presence of the  anion. Another trend can be identified between ionic liquids with the same anion and the identical type of functionalization, but with different cation structures such as . From Figures 3 and 4 it becomes evident that ionic liquids with  or  cations relax considerably faster than their  and  counterparts. This is observed independently of the anion and the side chain functionalization. To provide a more quantitative analysis, the experimental results were fitted according to the eqs 1, 3, 4, and 6. Overall, a very good agreement between the FFC experiment and the relaxation theory was found. However, for the  and  cation a deviation in the high-frequency regime between fit and experiment at the lowest temperature was identified. A possible explanation for this could be that the dynamics of the octyl-side chain of the supercooled ionic liquids cannot be sufficiently described through the relaxation models used. A more thorough analysis of this behavior is currently not possible and will be the scope of future work. For completeness, it is noted that all fits converged to a stable numerical solution, regardless of the initial values assigned to the fitting parameters. The influence of dipolar coupling between ^1^H and ^19^F spins on the overall relaxation was also investigated. The detailed analysis of this can be found in the Supporting Information, but it was concluded that the relaxation contribution due to dipolar coupling between ^1^H and ^19^F is small compared to the ^1^H–^1^H relaxation term. It is also noted that since the densities and gyromagnetic ratios of ^11^B and ^31^P are even smaller than for ^19^F, the relaxation contributions of ^11^B and ^31^P are also expected to be negligible. Those findings agree with the results reported by Kruk et al.,^15^ and consequently, for the following analysis presented in this paper, all non-^1^H–^1^H relaxation contributions were ignored and the whole relaxation process was solely attributed to dipolar coupling between ^1^H-spins. The resulting rotational and translational correlation times as well as the values of *b* and *d* employed in the fitting can be found in the Supporting Information. To identify the effects of the ether functionalization, the cation backbone structure and the choice of anion on the molecular dynamics of the ionic liquids, ratios of the translational and rotational correlation times were obtained from the data; i.e. the ratios τ_trans_/τ_trans_ and τ_rot_/τ_rot_ between different ionic liquid pairs were calculated. The results are given in Tables 1 and 2. For the sake of clarity, only the values at 283 K are presented, and the full set of ratios as a function of temperature are found in the Supporting Information. With reference to Tables 1 and 2, the following comparisons were made: (i) ether- and non-ether-functionalized ionic liquids with the same cation backbone structure and the same anion; (ii) ionic liquids with identical cations but different anions; and (iii) ionic liquids with the same functionalization and the same anion but with differing cation backbone structure. To facilitate the following discussion, we will refer to the first type as an alkyl/ether ratio, and the second will be an / ratio. The last one will be cited as a short/long ratio. From the alkyl/ether ratios it becomes evident, that ether functionalization results in certainly faster molecular dynamics for both rotational as well as translational movement. A comparison of the different alkyl/ether ratio reveals that the observed acceleration of rotation and translation is clearly the most significant for ionic liquids with a [P5551]^+^/ cation backbone structure. In addition to the backbone influence, the alkyl/ether ratios show a dependency in regards to the choice of the anion as well. For the [P5551]^+^/ cation backbone structure, the  anion results in a considerably stronger acceleration effect independent of the type of motion. In contrast, the trend is less clear for the [P2228]^+^/[P222(2O2O2)]^+^ pairs. In this case, no clear trend for the choice of anion can be identified. This means, that for rotational motion the  anion provides the more significant acceleration effect, whereas in the translational case the opposite holds true. Comparing the / pairs reveals a substantial deceleration of molecular dynamics, if the  anion is chosen. This effect is generally valid independent of the type of motion but more pronounced in the case of rotational movement. Between individual / pairs, it is not possible to identify a clear trend, which holds true for both modes of motion, but the  pair clearly shows the least significant deceleration for rotation as well as translation. Moving to the short/long ratios, it becomes evident that the alkyl-functionalized pairs show an acceleration of their overall dynamic behavior, if the [P2228]^+^ cation is chosen. This means that rotational as well as translational diffusion are increased for the [P2228]^+^ cation. In contrast, a comparison between the ether-functionalized short/long ratios reveals different effects for rotational and translational movement. In the case of rotation, an acceleration of the dynamics is observed, if the [P2228]^+^/[P222(2O2O2)]^+^ cation backbone structure is employed, which is consistent with the findings for the alkyl-functionalized short/long ratios. However, the opposite effect holds true for translational motion. This means that translational diffusion becomes reduced, if the [P222(2O2O2)]^+^ ion is chosen. The trends observed for the short/long ratios are valid for both anions.

**Table 1:** Rotational Correlation Time Ratios between Different Ionic Liquid Pairs

pair	τ_rot_/τ_rot_	pair	τ_rot_/τ_rot_	pair	τ_rot_/τ_rot_
[P5551][NTf_2_]/[P(2O2)_3_1][NTf_2_]	8.52	[P5551][NTf_2_]/[P5551][B(CN)_4_]	2.98	[P5551][NTf_2_]/[P2228][NTf_2_]	6.01
[P5551][B(CN)_4_]/[P(2O2)_3_1][B(CN)_4_]	4.65	[P2228][NTf_2_]/[P2228][B(CN)_4_]	2.61	[P5551][B(CN)_4_]/[P2228][B(CN)_4_]	5.27
[P2228][NTf_2_]/[P222(2O2O2)][NTf_2_]	2.26	[P(2O2)_3_1][NTf_2_]/[P(2O2)_3_1][B(CN)_4_]	1.63	[P(2O2)_3_1][NTf_2_]/[P222(2O2O2)][NTf_2_]	1.59
[P2228][B(CN)_4_]/[P222(2O2O2)][B(CN)_4_]	2.90	[P222(2O2O2)][NTf_2_]/[P222(2O2O2)][B(CN)_4_]	3.35	[P(2O2)_3_1][B(CN)_4_]/[P222(2O2O2)][B(CN)_4_]	3.29

**Table 2:** Translational Correlation Time Ratios between Different Ionic Liquid Pairs

pair	τ_trans_/τ_trans_	pair	τ_trans_/τ_trans_	pair	τ_trans_/τ_trans_
[P5551][NTf_2_]/[P(2O2)_3_1][NTf_2_]	8.10	[P5551][NTf_2_]/[P5551][B(CN)_4_]	1.69	[P5551][NTf_2_]/[P2228][NTf_2_]	1.83
[P5551][B(CN)_4_]/[P(2O2)_3_1][B(CN)_4_]	6.17	[P2228][NTf_2_]/[P2228][B(CN)_4_]	1.94	[P5551][B(CN)_4_]/[P2228][B(CN)_4_]	2.11
[P2228][NTf_2_]/[P222(2O2O2)][NTf_2_]	3.21	[P(2O2)_3_1][NTf_2_]/[P(2O2)_3_1][B(CN)_4_]	1.29	[P(2O2)_3_1][NTf_2_]/[P222(2O2O2)][NTf_2_]	0.73
[P2228][B(CN)_4_]/[P222(2O2O2)][B(CN)_4_]	2.72	[P222(2O2O2)][NTf_2_]/[P222(2O2O2)][B(CN)_4_]	1.65	[P(2O2)_3_1][B(CN)_4_]/[P222(2O2O2)][B(CN)_4_]	0.93

### Molecular Dynamics Simulations

The diffusion coefficients and diffusion ratios from the nonpolarizable simulations are given in the Supporting Information (Tables S13, S14, and S18) together with rotational correlation data (Tables S15, S16, and S19). Polarizable simulations were run at 298 K. The nonpolarizable simulations were run at 400 K to overcome the inherently slow dynamics. Thus, the resulting diffusion coefficients can only be interpreted in terms of relative trends.

The key aspect of the molecular dynamics simulations in this work are the targeted modifications of the dihedral angle parametrization. Here, the “linear”  cations were obtained by parametrizing the P–C–C–O dihedral angle with the *ab initio* potential energy scan of the P–C–C–C dihedral angle in the [P1115]^+^ model cation. Correspondingly, the “curled” [P5551]^+^ cations were obtained by parametrizing the P–C–C–C dihedral angle with the *ab initio* potential energy scan of the P–C–C–O dihedral angle in the [P111(2O2)]^+^ model cation. All other force field parameters are kept the same, thus any differences are solely due to the cation curling.

The diffusion coefficients from the nonpolarizable simulations indicate much faster diffusion of cations and anions in the ionic liquids with a native (curled)  cation compared to the ionic liquids where cations are restricted to the artificial, linear conformation. For the [P5551][NTf_2_] ionic liquid with the artificial curled cation, diffusion is slightly slower than for the linear cation.

Diffusion coefficients tend to increase with decreasing size of the cation (i.e., in the order [P5551][NTf_2_] < [P4441][NTf_2_] < [P3331][NTf_2_] < [P2221][NTf_2_]). However, even with the smallest cation, the diffusion is considerably slower than that for the ionic liquid [P(2O2)_3_1][NTf_2_] with the ether-functionalized cation. Furthermore, in the series with different alkyl side chain lengths, the change in size is coupled to a change in mass. For a better comparison, we considered a targeted modification of [P5551][NTf_2_] in the sense that the masses of the smaller cations were artificially scaled to that of [P5551]^+^ (“heavy” in Tables S18 and S20). The diffusion coefficients of cation and anion in [P(2O2)_3_1][NTf_2_] are more than twice those in [P2221][NTf_2_] with the heavy cation.

Polarizable simulations are desirable due to the higher accuracy, however they come at considerable additional computational effort. In our setup, about 250 to 500 CPU hours were required to run 1 ns of nonpolarizable MD simulation. In contrast, polarizable simulations required about 1300–4000 CPU hours per 1 ns of MD trajectory. For these reasons, polarizable simulations were only run for the ionic liquids with the  and [P5551]^+^ cations. The resulting diffusion coefficients and ratios thereof are shown in Table 3.

**Table 3:** Polarizable Simulations–Diffusion Coefficients at 298 K^a^

ionic liquid		10^12^*D*_self_^+^/	10^12^*D*_self_^–^/	*D*_self_^+^/*D*_self_^–^
		m^2^ s^–1^	m^2^ s^–1^	
[P(2O2)_3_1][NTf_2_]		27.1(3)	32.8(5)	0.83(2)
[P(2O2)_3_1][NTf_2_]	linear	3.8(2)	4.7(2)	0.79(5)
[P5551][NTf_2_]		8.0(4)	10.7(5)	0.74(5)
[P5551][NTf_2_]	curled	3.1(4)	3.8(1)	0.82(10)
[P(2O2)_3_1][B(CN)_4_]		4.0(1)	4.4(3)	0.89(6)
[P(2O2)_3_1][B(CN)_4_]	linear	0.49(4)	0.8(1)	0.62(10)
[P5551][B(CN)_4_]		2.1(1)	3.0(3)	0.70(8)

a Error estimates from independent MSD fits in the x-, y-, z-directions are given in parentheses.

Critically, the key result is the same as observed for the nonpolarizable simulations (i.e., the native (curled)  cations diffuse significantly faster than the linear  cations). The effect is also present in the anion diffusion. Furthermore, for the [P5551][NTf_2_] ionic liquid, introducing artificial curling into the cation slows down dynamics.

**Figure 5:**
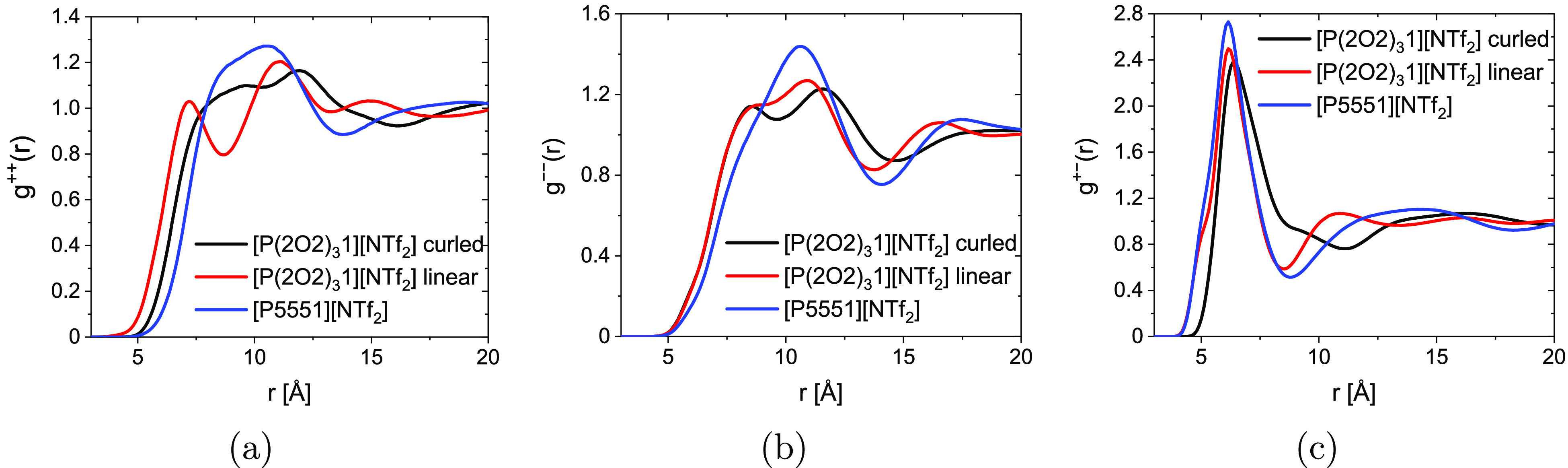
Radial distribution functions for (a) cation–cation, (b) anion–anion, and (c) cation–anion.

The experimentally observed differences between the dynamic properties of ether- and alkyl-functionalized ionic liquids are clearly linked to the curling of the cation. The radial distribution functions, Figure 5, allow further evaluation of the changes in structure caused by the curling. Only results for the best performing parametrization will be considered (i.e., the polarizable simulations with the  anion). Here we only present the radial distribution functions; more insight into structure–property relations can be gained from the spatial distribution functions, see the “Discussion” section.

The cation–cation radial distribution function (Figure 5a) shows a continuous shift to shorter distances for close contacts as well as a splitting of the first peak in the series [P(2O2)_3_1][NTf_2_] linear < [P(2O2)_3_1][NTf_2_] curled < [P5551][NTf_2_]. For the anion–anion radial distribution function (Figure 5c), [P(2O2)_3_1][NTf_2_] linear and [P(2O2)_3_1][NTf_2_] show very similar behavior (i.e., a split first peak in comparison with [P5551][NTf_2_]). Finally, the cation–anion radial distribution function (Figure 5c), reveals that [P(2O2)_3_1][NTf_2_] linear and [P5551][NTf_2_] show similar behavior, whereas the first peak for [P(2O2)_3_1][NTf_2_] curled is shifted to longer distances.

The polarizable simulations are expected to give relatively good agreement with experimental data due to the explicit representation of polarization effects and hence the response to electric fields, which is central for ionic liquids. The data together with comparison of experimental and simulated densities as well as diffusion coefficients are given in the Supporting Information. It is noticeable that the agreement is acceptable for ionic liquids with the  anion but not for those with the  anion. Furthermore, the diffusion of ions (with native cation conformation) in  ionic liquids is by a factor of 3–7 slower than that in  ionic liquids (Table 3). This is a qualitative difference to the case of the nonpolarizable force fields. We observed that upon introduction of polarizability, aggregates of  tend to form. This hints toward subtleties in the parametrization of the  anion that need to be explored. Hence we carried out two additional simulations for both [P(2O2)_3_1][B(CN)_4_] and [P5551][B(CN)_4_]. First, we used ADCH charges instead of CHELPG charges. Second, in addition to using ADCH charges, we introduced global Lennard-Jones scaling, not just for cation–anion interactions. The resulting diffusion coefficients are shown in Table 4.

**Table 4:** Diffusion Coefficients–Polarizable Simulations at 298 K where the Anion Is Parameterized with ADCH Charges

ionic liquid		10^12^*D*_self_^+^/	10^12^*D*_self_^–^/	*D*_self_^+^/*D*_self_^–^
		m^2^ s^–1^	m^2^ s^–1^	
[P(2O2)_3_1][B(CN)_4_]	ADCH	25(2)	33(3)	0.76(8)
[P(2O2)_3_1][B(CN)_4_]	ADCH *k*_++/––_	285(17)	297(25)	0.96(10)
[P5551][B(CN)_4_]	ADCH	8.2(4)	11.0(2)	0.74(4)
[P5551][B(CN)_4_]	ADCH *k*_++/––_	159(28)	166(13)	0.96(19)

Global Lennard-Jones scaling results in a strong overestimation of dynamics and underestimation of density, with diffusion coefficients that are an order of magnitude too large compared with the experiment. In contrast, the agreement between simulation and experiment is excellent for the simulation with ADCH charges but without global Lennard-Jones scaling, especially for the density. The full comparison is shown in the Supporting Information. Furthermore, a comparison of the anion–anion aggregation by means of anion–anion radial distribution functions is given in the Supporting Information.

## Discussion

Prior to discussion, it should be kept in mind, that in contrast to the physicochemical measurements, both the FFC analyses and the classical force fields innately contain assumptions which bias the results to some degree. However, this is not usually problematic if relative comparisons are pursued. For example, the absolute diffusion coefficients from an MD simulation change approximately by an order of magnitude between nonpolarizable and polarizable simulations; however, relative trends are conserved. Thus, in line with our general approach, we focus on relative results, comparing the changes between ionic liquids with systematically altered ion structures.

As previously reported, ionic liquids with ether chains form more compact cations as a result of the curling of the ether chains toward the positively charged cation center.^24,26^ The obtained trends for viscosity as well as conductivity are in agreement with the latter finding and show that the transport properties are distinctly affected by the side groups incorporated in the cation, as well as by the choice of the anion. In more detail, the remarkable acceleration of dynamics for the ether substituted ionic liquids compared to their alkyl counterparts can be clearly explained by the altered cation conformations. The curling of the ether chains thereby affects the geometry of the cation as well as the shielding of cation charge and occurs for the cations independent of the anions. The higher influence of the oxygen groups in the cations on the overall dynamics and ratios of substituted to nonsubstituted cation found for the / [P5551]^+^ pair compared to the substituted triethylphosphonium cations can be simply understood as being the result of a different number of ether groups. Therefore, the samples with 3-fold ether functionalization experience a higher boost of their dynamics compared to the alkylated counterparts, as is the case for the 2-fold ether substitution ([P222(2O2O2)]^+^ vs [P2228]^+^). A direct result of this are the higher ratios in transport properties for / [P5551]^+^ than for the [P222O2O2]^+^/ [P2228]^+^ samples. The similar trends for the two transport properties are the result of the inverse proportionality between the two, known as the Walden relation:
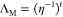
17with *t* being a fractional exponent close to unity.^57,58^

**Figure 6:**
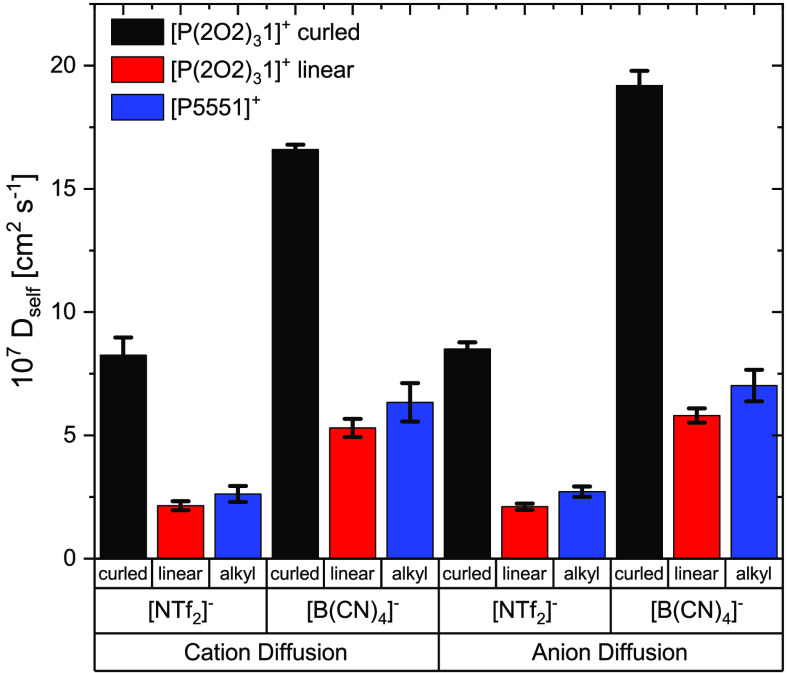
Diffusion coefficients obtained from nonpolarizable MD simulations.

**Figure 7:**
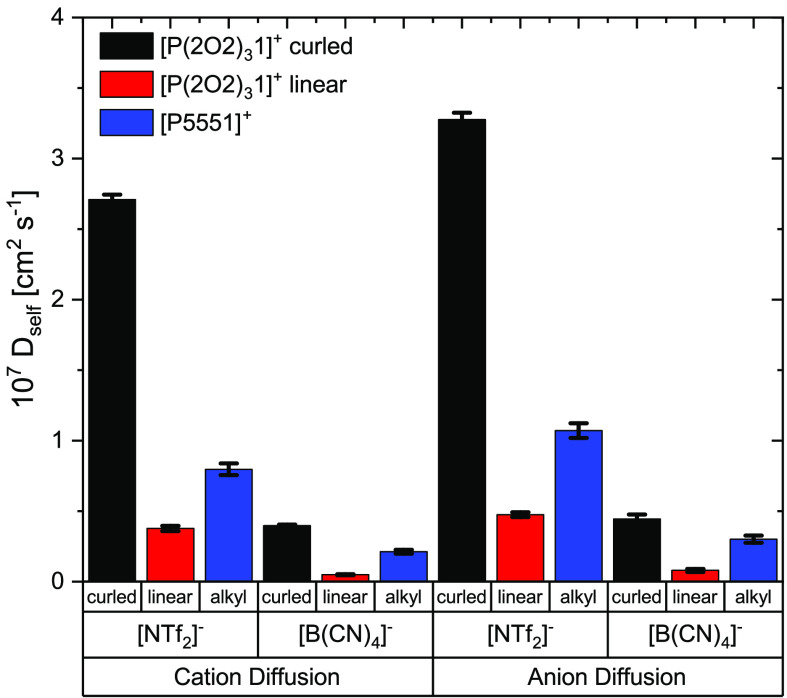
Diffusion coefficients obtained from polarizable MD simulations.

The diffusion coefficients from the MD simulations clearly show that it is indeed the curling of the cation which produces the desired acceleration of the dynamics in ether-functionalized ionic liquids (Figures 6 and 7). Critically, the effect is observed across all simulations, including nonpolarizable simulations as well as those which use the knowingly biased force field for the  anion.

**Figure 8:**
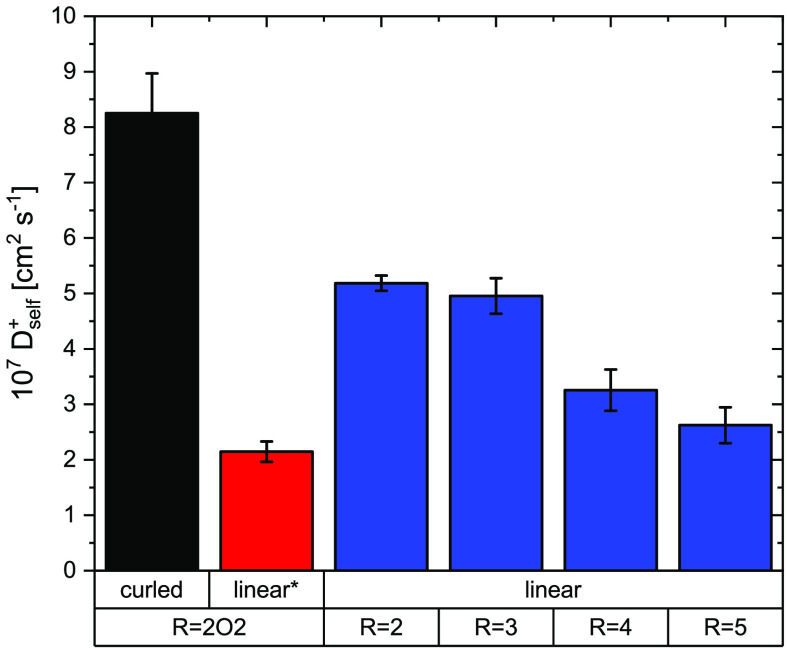
Size effect on diffusion coefficients. Here, the cation diffusion coefficients from the MD simulation are shown.

From the simulation alone, it is not clear why the curling of the ether-functionalized cations leads to such significant changes in the transport properties. The MD simulations show that the effect of the cation curling by far exceeds what would be expected for a mere size effect (see Figure 8 and the Supporting Information).

**Figure 9:**
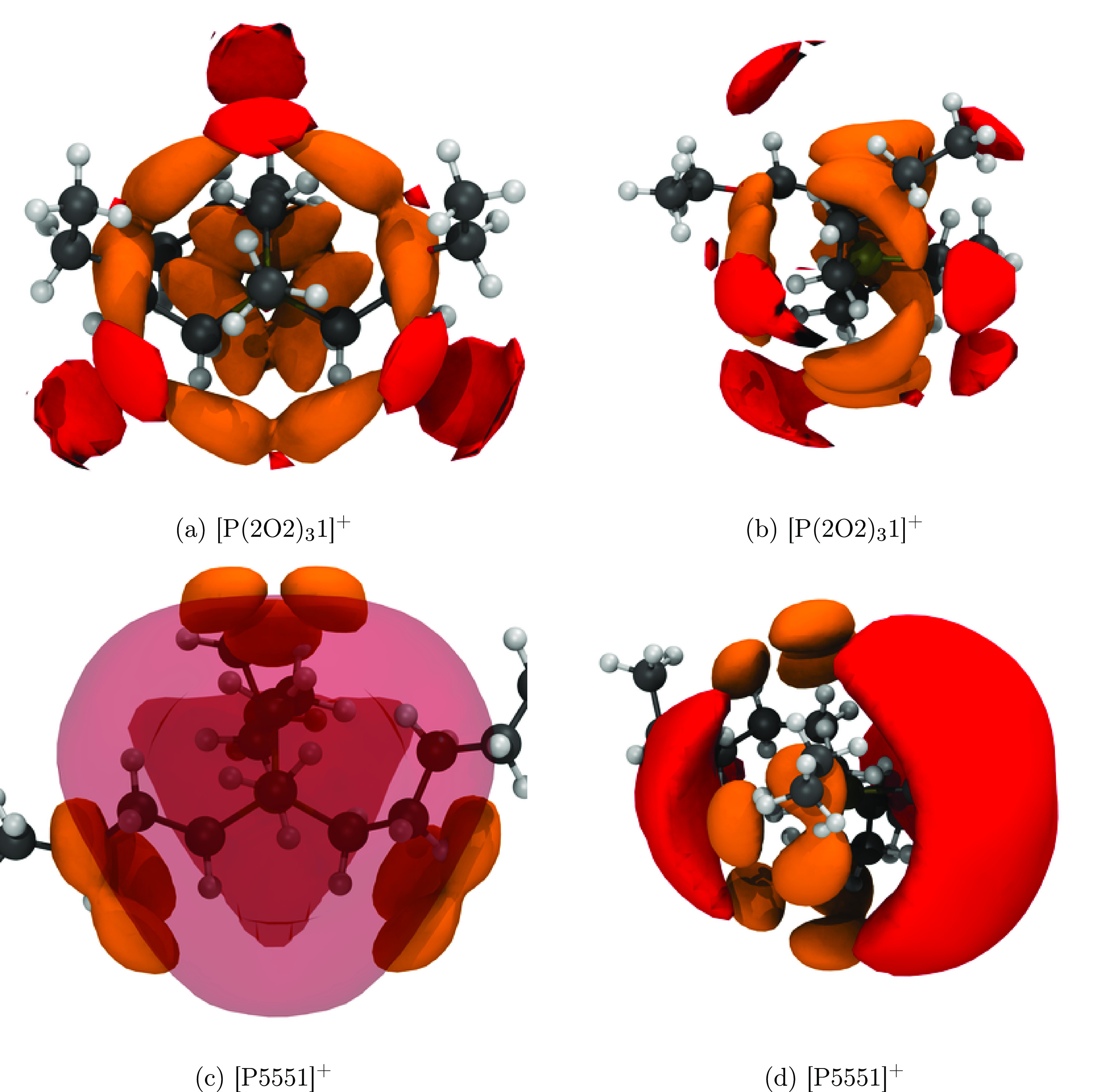
Spatial distribution function showing the shielding of the  and [P5551]^+^ cations. Red isosurfaces are drawn at an isovalue of 3 nm^–3^ and correspond to the intermolecular coordination via the oxygen atoms of the anions. Orange isosurfaces are drawn at an isovalue of 10 nm^–3^ and correspond to the intramolecular position of the ether or γ-CH_2_ groups. (a, b) [P(2O2)_3_1][NTf_2_] and (c, d) [P5551][NTf_2_]. For better comparison, the cations are oriented the same: (a, c) view on top of the CH_3_ group, in (b, d) the P–CH_3_ bond is in the paper plane, pointing to the right.

The trends of the cation–cation radial distribution functions (Figure 5a) cannot explain the observed effect of curling. The reason for this is that the observed trend, [P(2O2)_3_1][NTf_2_] linear < [P(2O2)_3_1][NTf_2_] curled < [P5551][NTf_2_], violates the trends observed in the (diffusive) dynamics. Similarly, the anion–anion radial distribution functions (Figure 5b) did not show a significant difference between the curled and linear [P(2O2)_3_1][NTf_2_]. Thus, the cation–anion radial distribution function (Figure 5c) is the most promising starting point to gain knowledge about how the curling affects the structure and thus the dynamics. This is not surprising given that the cation–anion interactions account for a major part of the Coulombic interaction energy. Here, spatial distribution functions allow for additional insight due to the angular resolution. Figure 9a,b show that the curled side chains in  occupy regions of space which would otherwise be preferable for anion coordination. In contrast, the [P5551]^+^ cation shows very well-defined cation–anion interactions (Figure 9c,d), leading to the close contacts in the radial distribution function.

**Figure 10:**
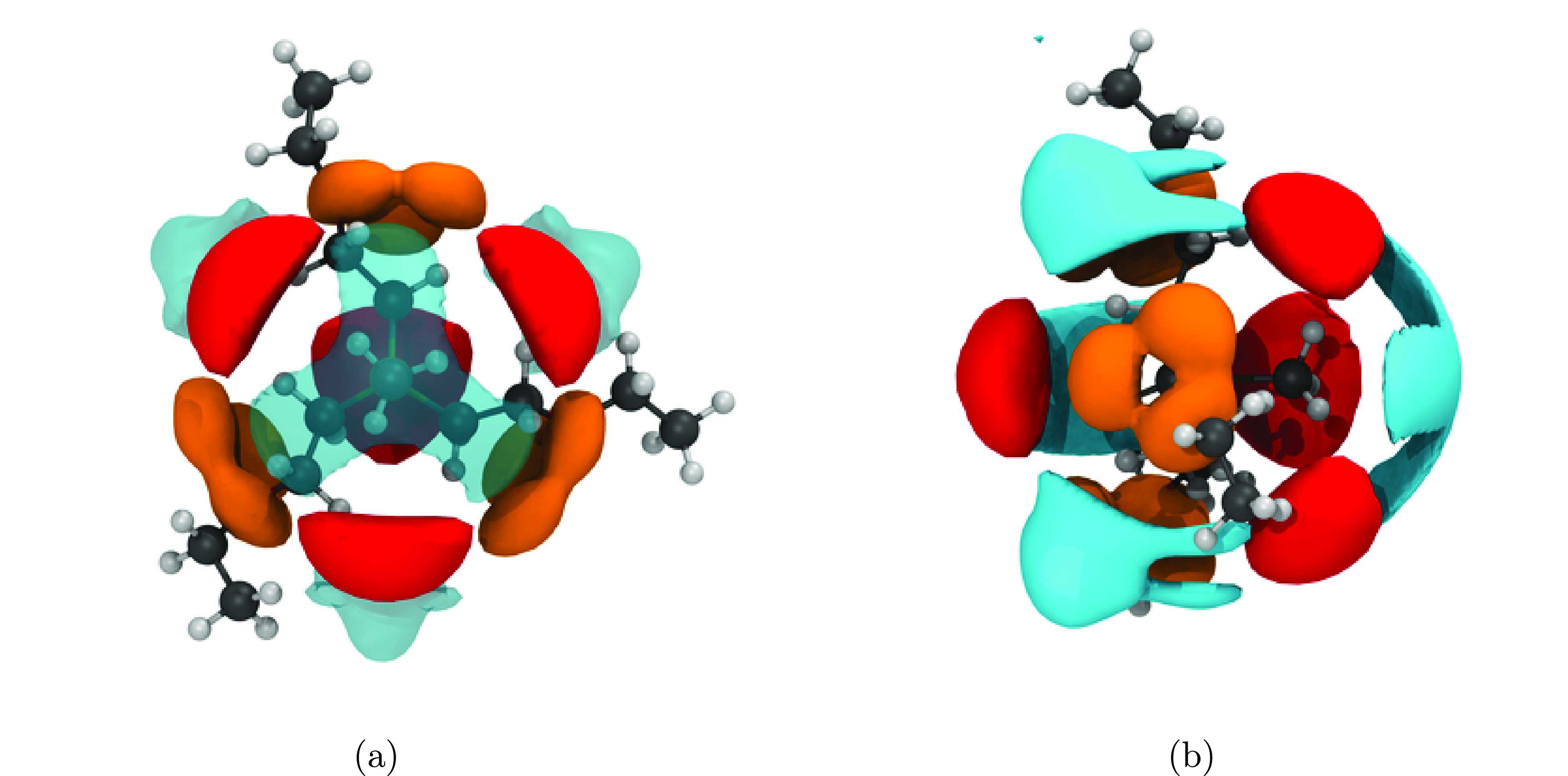
Spatial distribution functions around the cation in the linear [P(2O2)_3_1][NTf_2_]. The red isosurface is drawn at an isovalue of 5 nm^–3^ and corresponds to the intermolecular coordination via the oxygen atoms of the anions. The orange isosurface is drawn at an isovalue of 10 nm^–3^ and correspond to the in intramolecular position of the ether oxygens. The blue isosurface is drawn at 2 nm^–3^ and shows the intermolecular position of ether oxygens in other cations. (a) view on top of the CH_3_ group, in (b) the P–CH_3_ bond is in the paper plane, pointing to the right.

The MD simulations of non-native conformations allow for profound insight beyond what is accessible to the other methods. It has been observed that the effect of curling goes beyond that of a pure size effect and that the cation–anion interactions play a crucial role. The spatial distribution function based on the non-native linear  cation (Figure 10) explains why the dynamics in this ionic liquid are lower than that in the (native) [P5551][NTf_2_]. The artificially extended, linear ether side chains are no longer capable of shielding the cation. Thus, the anion oxygen atoms occupy well-defined, energetically favorable locations (cf., Figure 9c,d; the red isosurface is drawn at a higher isovalue compared to the other Figures to avoid occlusion). However, the polar ether oxygen atoms also induce additional cation–cation interactions, which explain the lower dynamics as well as the closer cation–cation contacts in Figure 5a.

**Figure 11:**
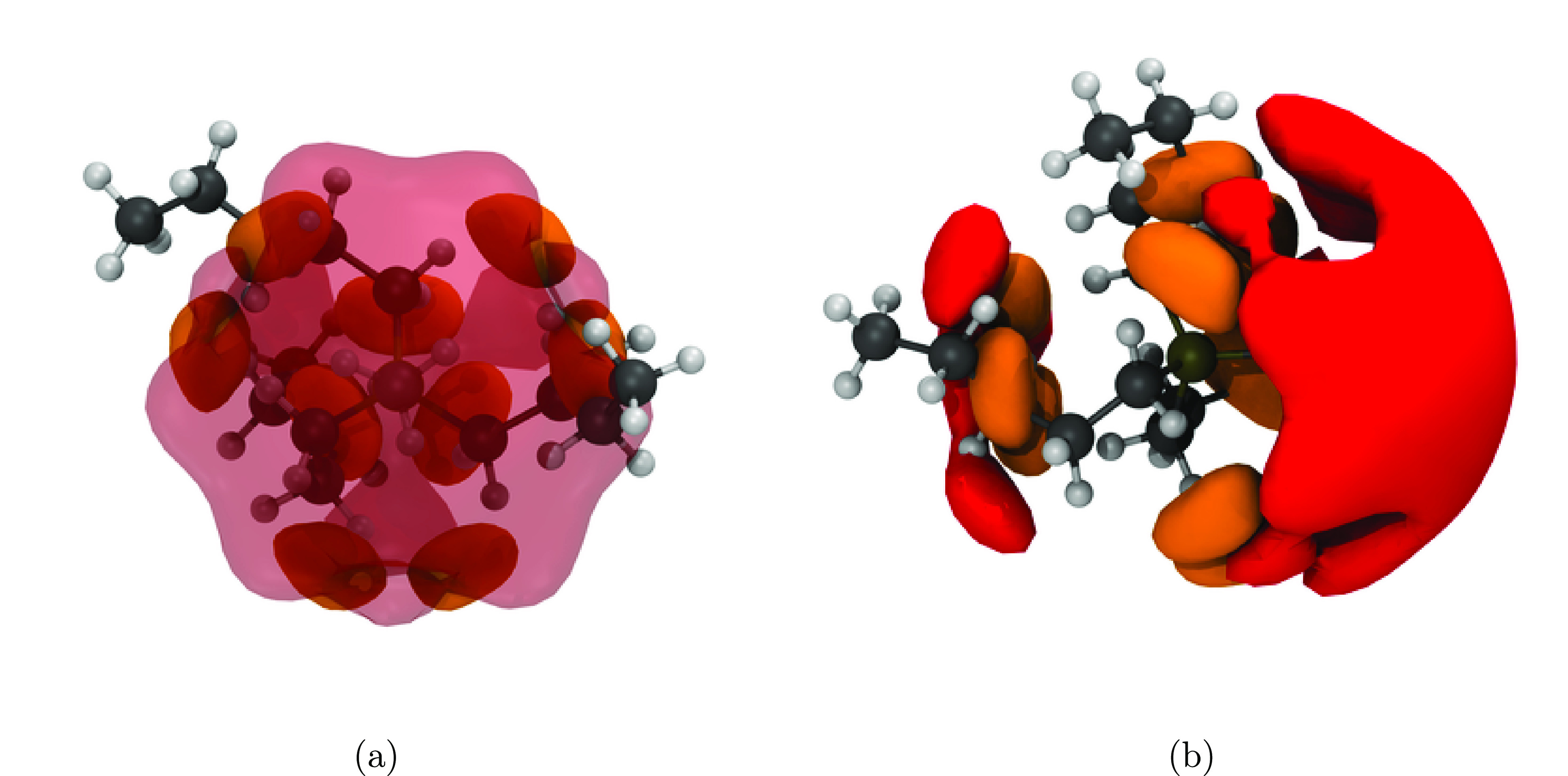
Spatial distribution functions around the cation in the curled [P5551][NTf_2_]. The red isosurface is drawn at an isovalue of 3 nm^–3^ and corresponds to the intermolecular coordination via the oxygen atoms of the anions. The orange isosurface is drawn at an isovalue of 10 nm^–3^ and correspond to the in intramolecular position of the ether oxygens. (a) View on top of the CH_3_ group; in (b) the P–CH_3_ bond is in the paper plane, pointing to the right.

The slow dynamics for the [P5551][NTf_2_] ionic liquid with the artificially curled cation furthermore show that the curled geometry alone is not sufficient to explain the fast diffusion of its ether analogue. In addition, as discussed previously, size is not sufficient to explain experimental observations. Indeed, the red isosurfaces in Figure 11 closely resemble those obtained for the native linear cation geometry, even though the orange isosurfaces confirm that the cation assumes the curled geometry. Thus, we conclude that the increased diffusion of the ether-functionalized cations originates from the shielding of the cation core and goes beyond a mere size effect.

Considering the FFC analysis, the observed temperature and frequency dependencies of the longitudinal relaxation rate are in agreement with the predictions made by relaxation theory, if the Redfield limit is fulfilled. As discussed earlier, the Redfield limit is considered to be clearly valid for the ionic liquids considered here, which means that an increase in temperature leads to an overall acceleration of molecular dynamics, resulting in a more efficient averaging process of the nonsecular parts of the dipolar Hamiltonian. As a result of this the relaxation rate decreases, which is exactly the temperature-dependent behavior that was observed in this study.^13,28^ The differences in the relaxation behavior between the samples can be explained as follows. The slower relaxation rate for ether-functionalized ionic liquids in comparison to their alkyl-counterparts provides evidence that ether functionalization has to result in overall faster molecular dynamics. Analogous to the explanation of the temperature dependence of the relaxation rate, it can be inferred that the acceleration of the dynamic behavior of the cation results in a more effective averaging process of the nonsecular parts of the dipolar Hamiltonian, which eventually explains the slower relaxation for ether-functionalized ionic liquids. This trend also agrees with the findings from physicochemical measurements and MD simulations. The viscosity measurements in this study reveal a drop in viscosity due to ether functionalization. Consequently, accelerated motion on a microscopic level is supported by the viscosity data. The same holds true for the MD simulations. The predicted curling effect and the accompanied shielding of the cation charge result in an acceleration of rotational as well as translation motion due to the smaller size of the curled cation and the reduced electrostatic interaction between the shielded cation and anion.

The differences in the relaxation behavior of ionic liquids, which only differ in the choice of the anion, can also be explained using the same arguments from relaxation theory as used to understand the effect of ether functionalization. Thus, the  anion is expected to accelerate the overall dynamic behavior of the sample in comparison to the  anion. This finding is an agreement with the viscosity data, which reveal lower viscosities for  ionic liquids. However, a more thorough analysis of the molecular origins of this effect is behind the scope of this study.

The different relaxation behavior of ionic liquids with differing cation backbone structure but with identical anions and the same type of functionalization can be explained by the different sizes of the cations. Cations with a [P5551]^+^/ backbone structure contain 16 carbon atoms or 13 carbon atoms and 3 oxygens in the side chains, whereas the [P2228]^+^/[P222(2O2O2)]^+^ cations only consist of 14 carbon atoms or 12 carbon atoms and 2 oxygens. This results in a smaller size of the cations with the [P2228]^+^/[P222(2O2O2)]^+^ backbone structure that facilitates diffusion and consequently, resulting in a decrease of the relaxation rate due to more effective motional averaging of the dipolar perturbations.

As already inferred from the NMRD profiles, the alkyl/ether ratios provide further clear evidence for the acceleration effect due to ether functionalization. They also show that the acceleration effect is significantly stronger for ionic liquids with a [P5551]^+^/[P(2O2)_3_1]^+^ backbone structure. This finding can be rationalized in combination with the results of the MD simulations and the viscosity measurements. On the current level of analysis, it is considered that the curling and shielding effect is more pronounced for the cation than for the [P222(2O2O2)]^+^ cation. Consequently, the magnitude of acceleration is anticipated to be smaller for the [P222(2O2O2)]^+^ cation, and subsequently, it is expected to result in less accelerated molecular dynamics, which agrees with the obtained viscosity trends. However, due to the lack of polarizable MD simulations for ionic liquids with the [P222(2O2O2)]^+^ cation, it is not possible to provide a more thorough explanation for the observed differences at the moment.

Furthermore, the / correlation time ratios show a general deceleration of the dynamic behavior; the  is chosen as an anion. This is in agreement with the viscosity measurements and the analysis of relaxation data, but due to the significant chemical differences of the  and  anions, a more thorough comparison of the anions is beyond the scope of this study.

The acceleration effect, which is observed for the alkyl-functionalized short/long ratios, is considered to be linked to the different size of the cations. As discussed earlier, the [P2228]^+^ cation has two methylene groups less than the [P5551]^+^ cation, which is expected to result in a smaller cation size and, therefore, anticipated to facilitate both types of diffusion. The opposed trends for rotational and translational motion, which can be observed for the ether-functionalized short/long ratios can be explained as follows. The shielding of the cation charge due to the curling process is considered to reduce the electrostatic attraction between the cation and anion, which is anticipated to accelerate translational diffusion. As discussed earlier, the magnitude of the curling effect is higher for the  cation in comparison to that for the [P222(2O2O2)]^+^ cation, which explains the slower translational dynamics of the [P222(2O2O2)]^+^ ionic liquids. In contrast, under the assumption of a sufficiently symmetric charge distribution around the phosphorus in the cation, the shielding is expected to be of less importance for rotational diffusion, and consequently, the cation-size effect, which was already discussed for the alkyl-functionalized short/long ratios, eventually prevails, explaining the opposed trends between rotational and translational motion. At this point, it should also be mentioned that evidence for the latter assumption of a symmetric charge distribution can be found in form of electrostatic potential plots in the Supporting Information.

## Conclusions

The dynamics in ionic liquids are still poorly understood, despite the many emerging practical applications. This is particularly true for rotational dynamics, for which experimental data are scarce. Here we report FFC measurements that allow for the simultaneous quantification of both translational and rotational dynamics. The combination with MD simulations allows us to identify molecular mechanisms determining the macroscopic properties. Focus was put on the understanding and rationalization of relative trends, so the structure–property relationships identified in this work may be deployed as design elements in the future.

We chose to investigate ionic liquids with two anions, the  anion and the  anion.  is a convenient choice for FFC measurements as it does not contain ^1^H or ^19^F nuclei. In contrast, the  anion commonly used in ionic liquids contains ^19^F atoms that could complicate the modeling of relaxation. However, modeling of the FFC data showed that ^19^F relaxation contribution is negligible. The FFC data for all ionic liquids, with both anions, could be well-fit with the models used.

The cations in this work were chosen to have similar sizes and masses, and the variations were alkyl/ether and long/short side chains. All ionic liquids were subject to comprehensive physicochemical characterization. Critically, ether functionalization was found to significantly and consistently lower viscosities, which is a key property of ionic liquids.

The FFC measurements and physicochemical characterization was complemented with systematical molecular dynamics simulations. We carried out both nonpolarizable and polarizable simulations allowing for relative and absolute predictions, respectively. Good agreement between simulation and experiments was observed, although there were crucial subtleties in the parametrization of atomic charges which had to be addressed. In particular, we observed evidence for the aggregation of  anions within the ionic liquid, which will be the subject of future work.

Importantly, the FFC relaxation behavior agrees with the findings from the simulations and physicochemical measurements. On the basis of this successful and consistent combination of different methods and building on previous work, we were able to demonstrate that cation curling is the underlying cause for the fast dynamics in ionic liquids with ether-functionalized cations. Furthermore, the effects of cation curling significantly exceed what would be expected from a pure size effect.

## Notes

Force field input files for use with fftool are available at https://github.com/paduagroup/fftool.

## Supporting Information Available

Key results from molecular dynamics simulations analyses (radial distribution functions, rotational autocorrelation functions for charge arm and the P–CH_3_ vector both using the first and second Legendre polynomial, α_2_(*t*), charge arm distribution, charge lever moment distribution, mean squared displacements, FFC distances), exemplary input files for MD simulations, results from *ab initio* scans for parametrization and input *z*-matrices, atomic charges and corresponding Gaussian log files/Multiwfn.chg files, comparison of ADCH and CHELPG charges, preparation and equilibration procedure, and bond and angle fit curves (PDF)
