# Supplementary material for: Molecular Dynamics of Ionic Liquids from Fast-Field Cycling NMR and Molecular Dynamics Simulations
Source: J Phys Chem B. 2022 Sep 12;126(37):7143–58. doi: 10.1021/acs.jpcb.2c01372 (PMC9511496; doi:10.1021/acs.jpcb.2c01372)
Supplement: Supplementary file 1 — jp2c01372_si_001.pdf [file jp2c01372_si_001.pdf]

Molecular Dynamics of Ionic Liquids from Fast Field Cycling NMR and Molecular Dynamics Simulations - Supporting Information

Julian B. B. Beckmann,[†] Daniel Rauber,[‡] Frederik Philippi,[¶] Kateryna Goloviznina,[§] Jordan A. Ward-Williams,[†] Andy J. Sederman,[†] Mick D. Mantle,[†] Agílio Pádua,[§] Christopher W. M. Kay,[‡,‖] Tom Welton,[¶] and Lynn F. Gladden[*,†]

†*Department of Chemical Engineering and Biotechnology, University of Cambridge, Philippa Fawcett Drive, Cambridge CB3 0AS, United Kingdom*

‡*Department of Chemistry, Saarland University, Campus B2.2, 66123 Saarbrücken, Germany*

¶*Department of Chemistry, Molecular Sciences Research Hub, Imperial College London, White City Campus, London W12 0BZ, UK*

§*Laboratoire de Chimie, École Normale Supérieure de Lyon & CNRS, 69364 Lyon, France*

‖*London Centre for Nanotechnology, University College London, 17-19 Gordon Street, London WC1H 0AH, United Kingdom*

E-mail: lfg1@cam.ac.uk

Syntheses

The general procedure for ionic liquid metathesis is the same as described in the supporting information elsewhere.[1] Furthermore, $[P(2O2)_3 1]I$, [P5551]I and $K[B(CN)_4]$ were prepared as described in the literature.[1,2]

2-(2-Ethoxyethoxy)ethyl bromide was prepared *via* Appel reaction. First, 75.81 g (565 mmol / 1.00 eq) of 2-(2-ethoxyethoxy)ethanol were dissolved in 800 mL THF in a 2 L round bottom flask and cooled to 0°C. To this solution, 204.37 g (616 mmol, 1.09 eq) carbon tetrabromide and 160.92 g (614 mmol, 1.09 eq) triphenylphosphine were added together in 7 portions over the course of 2 h. After addition was complete, the reaction mixture was stirred for another 2 h and then stirred at room temperature for 13 h. Then, 900 mL of hexane were added and the precipitated solids were filtered off, washing the filter cake with 100 mL of hexane. The organic phase was washed with 250 ml and then with 100 mL water, dried over $MgSO_4$ and filtered again. After removal of the bulk solvent by means of rotary evaporation, the residue was stirred in high vacuum overnight. The raw material was then distilled over a fractionating column three times to remove bromoform and other impurities, giving 86.5 g of 2-(2-ethoxyethoxy)ethyl bromide (439 mmol / 78% yield). ^{1}H NMR ($CDCl_3$, 400 MHz, δ in ppm): 3.80 (t, $^3J_{H/H}$ = 6.4 Hz, 2H, Br−$\mathbf{CH_2}$−CH_2−O), 3.68-3.55 (m, 4H, −O−$\mathbf{CH_2}$−$\mathbf{CH_2}$−O−), 3.52 (q, $^3J_{H/H}$ = 7.0 Hz, 2H, O−$\mathbf{CH_2}$−CH_3), 3.46 (t, $^3J_{H/H}$ = 6.4 Hz, 2H, Br−CH_2−$\mathbf{CH_2}$−O), 1.20 (t, $^3J_{H/H}$ = 7.0 Hz, 3H, O−CH_2−$\mathbf{CH_3}$); $^{13}C\{^1H\}$ NMR ($CDCl_3$, 101 MHz, δ in ppm): 71.34 (s, Br−$\mathbf{CH_2}$−CH_2−O), 70.71 (s, −O−$\mathbf{CH_2}$−$\mathbf{CH_2}$−O−), 69.91 (s, −O−$\mathbf{CH_2}$−$\mathbf{CH_2}$−O−), 66.82 (s, O−$\mathbf{CH_2}$−CH_3), 30.34 (s, Br−CH_2−$\mathbf{CH_2}$−O), 15.27 (s, O−CH_2−$\mathbf{CH_3}$).

[P2228][Br] was prepared by reaction of triethylphosphine with 1-bromooctane. 6.38 g of the phosphine (5.11 g / 43.2 mmo l/ 1.00 eq) were dissolved in 120 mL of dry, degassed acetonitrile under argon and 8.27 mL of the 1-alkyl bromide (9.18 g / 47.5 mmol / 1.20 eq.) were added. The homogeneous reaction mixture was stirred for 3 days at 35°C with intense stirring. The solvent was removed by rotary evaporation and the residual solid was dried in

high vacuum for two days to obtain 13.2 g of the title compound [P2228][Br] (42.4 mmol / 98%) as a white solid. ^{1}H NMR ($CDCl_3$, 400 MHz, δ in ppm): 2.51 (dq, $^2J_{H/P}$ = 13.0 Hz, $^3J_{H/H}$ = 7.7 Hz, 6H, P−$\mathbf{CH_2}$−CH_3), 2.44-2.38 (m, 2H), 1.61-1.39 (m, 4H), 1.34-1.16 (m, 17H), 0.83 (t, 3H); ^{13}C{^{1}H} NMR (DMSO−d_6, 101 MHz, δ in ppm): 31.63 (s), 30.82 (d, $^3J_{C/P}$ = 14.4 Hz, P−CH_2−CH_2−$\mathbf{CH_2}$), 28.93 (s), 28.91 (s), 22.53 (s), 21.73 (d, $^2J_{C/P}$ = 4.7 Hz, P−CH_2−$\mathbf{CH_2}$−CH_2), 18.21 (d, $^1J_{C/P}$ = 47.1 Hz, P−$\mathbf{CH_2}$−CH_2−CH_2), 14.02 (s, −CH_2−CH_2−$\mathbf{CH_3}$), 12.42 (d, $^1J_{C/P}$ = 48.8 Hz, P−$\mathbf{CH_2}$−CH_3), 6.13 (d, $^2J_{C/P}$ = 5.23 Hz, P−CH_2−$\mathbf{CH_3}$); ^{31}P{^{1}H} NMR (DMSO−d_6, 162 MHz, δ in ppm): 38.04 (s).

[P222(2O2O2)][Br] was prepared by nucleophilic substitution reaction between triethylphosphine and 2-(2-ethoxyethoxy)ethyl bromide similar to the preparation of [P2228]Br. 12.6 mL of the phosphine (10.11 g / 85.6 mmol / 1.10 eq) were added to approximately 150 mL of dry acetonitrile under argon. To the solution 11.8 mL (15.3 g/ 77.8 mmol / 1.00 eq) of the ether-bromide were added and the mixture was stirred for 7 days at ambient temperature. The solvent and excess phosphine were removed using a rotary evaporator and the residue dried on a Schlenk line for two days giving 23.3 g of [P222(2O2O2)]Br (73.9 mmol / 95%) as a white solid.

^{1}H NMR ($CDCl_3$, 400 MHz, δ in ppm): 3.88 (dt, $^3J_{H/P}$ = 19.3 Hz, $^3J_{H/H}$ = 6.1 Hz, 2H, P−CH_2−$\mathbf{CH_2}$−O), 3.66-3.48 (m, 4H, O−$\mathbf{CH_2}$−$\mathbf{CH_2}$−O), 3.45 (q, $^3J_{H/H}$ = 7.0 Hz, 2H, O−$\mathbf{CH_2}$−CH_3), 2.95 (dt, $^2J_{H/P}$ = 12.1 Hz, $^3J_{H/H}$ = 5.9 Hz, P−$\mathbf{CH_2}$−CH_2−O), 2.51 (dq, $^2J_{H/P}$ = 13.4 Hz, $^3J_{H/H}$ = 7.7 Hz, 6H, P−$\mathbf{CH_2}$−CH_3), 1.28 (dt, $3\,J_{H/P}$ = 18.4 Hz, $^3J_{H/H}$ = 7.7 Hz, 9H, P−CH_2−$\mathbf{CH_3}$), 1.15 (t, $^3J_{H/H}$ = 7.0 Hz, 3H, O−CH_2−$\mathbf{CH_3}$); ^{13}C{^{1}H} NMR ($CDCl_3$, 101 MHz, δ in ppm): 69.63 (s), 69.24 (s), 66.48 (s), 64.06 (d, $^2J_{C/P}$ = 7.9 Hz, P−CH_2−$\mathbf{CH_2}$−O), 20.35 (d, $^1J_{C/P}$ = 49.9 Hz, P−$\mathbf{CH_2}$−CH_2−O), 15.22 (s, −O−CH_2−$\mathbf{CH_3}$), 13.10 (d, $^1J_{C/P}$ = 48.7 Hz, P−$\mathbf{CH_2}$−CH_3), 6.13 (d, $^2J_{C/P}$ = 5.5 Hz, P−CH_2−$\mathbf{CH_3}$); ^{31}P{^{1}H} NMR ($CDCl_3$, 162 MHz, δ in ppm): 38.78 (s).

[P2228][B(CN)$_4$] was prepared by metathesis from 10.19 g [P2228]Br (32.7 mmol / 1.00 eq) and 5.90 g K[B(CN)$_4$] (38.3 mmol / 1.17 eq) in 200 mL CH_2Cl_2 and 30 mL H_2O. The

organic phase was washed three times with 30 mL H_2O each, removal of the solvent by means of rotary evaporation followed by drying in high vacuum gave 11.03 g of [P2228][B(CN)$_4$] (31.9 mmol / 97%). ^{1}H NMR ($CDCl_3$, 400 MHz, δ in ppm): 2.18 (dq, $^2J_{H/P}$ = 12.5 Hz, $^3J_{H/H}$ = 7.7 Hz, 6H, P−**CH**$_2$−CH$_3$), 2.13-2.04 (m, 2H), 1.62-1.45 (m, 4H), 1.38-1.21 (m, 8H), 1.32 (dt, 3 $J_{H/P}$ = 18.2 Hz, $^3J_{H/H}$ = 7.7 Hz, 9H, P−CH$_2$−**CH**$_3$), 0.91-0.85 (m, 3H); ^{13}C{^{1}H} NMR (DMSO−d$_6$, 101 MHz, δ in ppm): 121.76 (q, $^1J_{C/B}$ = 70.8 Hz, ^{11}B−**CN**), 121.76 (sep, $^1J_{C/B}$ = 23.6 Hz, ^{10}B−**CN**), 31.21 (s), 30.15 (d, $^3J_{C/P}$ = 15.0 Hz, P−CH$_2$−CH$_2$−**CH**$_2$), 28.42 (s), 28.22 (s), 22.06 (s), 20.48 (d, $^2J_{C/P}$ = 4.2 Hz, P−CH$_2$−**CH**$_2$−CH$_2$), 16.40 (d, $^1J_{C/P}$ = 47.5 Hz, P−**CH**$_2$−CH$_2$−CH$_2$), 13.92 (s, −CH$_2$−CH$_2$−**CH**$_3$), 10.52 (d, $^1J_{C/P}$ = 48.8 Hz, P−**CH**$_2$−CH$_3$), 5.15 (d, $^2J_{C/P}$ = 5.23 Hz, P−CH$_2$−**CH**$_3$); ^{31}P{^{1}H} NMR (DMSO−d$_6$, 162 MHz, δ in ppm): 38.97 (s); ^{11}B NMR ($CDCl_3$, 128 MHz, δ in ppm): −38.21 (s). HRMS, ESI$^+$: m/z found 231.2238, calc. 231.2242 ($PC_{14}H_{32}^+$); ESI$^-$: m/z found 115.0214, calc. 115.0216 ($^{11}BC_4N_4^-$). Elemental analysis: calculated for $C_{18}H_{32}BN_4P$: C, 62.44; H, 9.32; N, 16.18. Found C, 61.79; H, 9.79; N, 15.84.

[P222(2O2O2)][B(CN)$_4$] was prepared by metathesis from 10.31 g [P222(2O2O2)]Br (32.7 mmol / 1.00 eq) and 5.87 g K[B(CN)$_4$] (38.1 mmol / 1.17 eq) in 200 mL CH_2Cl_2 and 30 mL H_2O. The organic phase was washed three times with 20 mL H_2O each, removal of the solvent by means of rotary evaporation followed by drying in high vacuum gave 10.94 g of [P222(2O2O2)][B(CN)$_4$] (31.2 mmol / 95%). ^{1}H NMR (DMSO−d$_6$, 400 MHz, δ in ppm): 3.74 (dt, $^3J_{H/P}$ = 19.0 Hz, $^3J_{H/H}$ = 6.1 Hz, 2H, P−CH$_2$−**CH**$_2$−O), 3.59-3.47 (m, 4H, O−**CH**$_2$−**CH**$_2$−O), 3.44 (q, $^3J_{H/H}$ = 7.0 Hz, 2H, O−**CH**$_2$−CH$_3$), 2.56 (dt, $^2J_{H/P}$ = 12.4 Hz, $^3J_{H/H}$ = 6.0 Hz, P−**CH**$_2$−CH$_2$−O), 2.24 (dq, $^2J_{H/P}$ = 13.5 Hz, $^3J_{H/H}$ = 7.6 Hz, 6H, P−**CH**$_2$−CH$_3$), 1.15 (dt, 3 $J_{H/P}$ = 18.3 Hz, $^3J_{H/H}$ = 7.7 Hz, 9H, P−CH$_2$−**CH**$_3$), 1.11 (t, $^3J_{H/H}$ = 7.0 Hz, 3H, O−CH$_2$−**CH**$_3$); ^{13}C{^{1}H} NMR (DMSO−d$_6$, 101 MHz, δ in ppm): 121.78 (q, $^1J_{C/B}$ = 70.8 Hz, ^{11}B−**CN**), 121.78 (sep, $^1J_{C/B}$ = 23.7 Hz, ^{10}B−**CN**), 69.63 (s), 68.87 (s), 65.51 (s), 63.10 (d, $^2J_{C/P}$ = 6.7 Hz, P−CH$_2$−**CH**$_2$−O), 18.30 (d, $^1J_{C/P}$ = 49.0 Hz, P−**CH**$_2$−CH$_2$−O), 15.07 (s, −CH$_2$−CH$_2$−**CH**$_3$), 11.40 (d, $^1J_{C/P}$ = 48.6 Hz, P−**CH**$_2$−CH$_3$),

5.21 (d, $^2J_{C/P}$ = 5.29 Hz, P−CH_2−**CH_3**); $^{31}P\{^1H\}$ NMR (DMSO−d_6, 162 MHz, δ in ppm): 39.28 (s); ^{11}B NMR (DMSO−d_6, 128 MHz, δ in ppm): −38.53 (s). HRMS, ESI^+: m/z found 235.1825, calc. 235.1827 ($PC_{12}H_{28}O_2{}^+$); ESI^-: m/z found 115.0211, calc. 115.0216 ($^{11}BC_4N_4{}^-$). Elemental analysis: calculated for $C_{16}H_{28}BN_4O_2P$: C, 54.87; H, 8.06; N, 16.00. Found C, 54.80; H, 8.35; N, 16.01.

[P5551][B(CN)$_4$] was prepared by metathesis from 5.88 g [P5551]I (15.2 mmol / 1.00 eq) and 2.84 g K[B(CN)$_4$] (18.4 mmol / 1.21 eq) in 200 mL CH_2Cl_2 and 20 mL H_2O. The organic phase was washed three times with 20 mL H_2O each, removal of the solvent by means of rotary evaporation followed by drying in high vacuum gave 5.42 g of [P5551][B(CN)$_4$] (14.5 mmol / 95%). ^1H NMR (DMSO−d_6, 400 MHz, δ in ppm): 2.22-2.07 (m, 6H, P−**CH_2**−CH_2), 1.78 (d, $^2J_{H/P}$ = 14.0 Hz, 3H, P−**CH_3**), 1.56-1.43 (m, 6H, P−CH_2−**CH_2**), 1.43-1.27 (m, 12H, P−$(CH_2)_2$−(**CH_2**)$_2$), 0.91 (t, $^3J_{H/H}$ = 6.9 Hz, 9H, P−$(CH_2)_4$−**CH_3**); $^{13}C\{^1H\}$ NMR (DMSO−d_6, 101 MHz, δ in ppm): 121.73 (q, $^1J_{C/B}$ = 70.8 Hz, ^{11}B−**CN**), 121.73 (sep, $^1J_{C/B}$ = 23.8 Hz, ^{10}B−**CN**), 32.13 (d, $^3J_{C/P}$ = 15.5 Hz, P−CH_2−CH_2−**CH_2**), 21.33 (s, P−$(CH_2)_3$−**CH_2**), 20.19 (d, $^2J_{C/P}$ = 4.4 Hz, P−CH_2−**CH_2**), 19.09 (d, $^1J_{C/P}$ = 49.1 Hz, P−**CH_2**−CH_2), 13.49 (s, P−$(CH_2)_4$−**CH_3**, 3.08 (d, $^1J_{C/P}$ = 51.7 Hz, P−**CH_3**); $^{31}P\{^1H\}$ NMR (DMSO−d_6, 162 MHz, δ in ppm): 32.24 (s); ^{11}B NMR (DMSO−d_6, 128 MHz, δ in ppm): −38.53 (s). HRMS, ESI^+: m/z found 259.2547, calc. 259.2555 ($PC_{16}H_{36}{}^+$); ESI^-: m/z found 115.0220, calc. 115.0216 ($^{11}BC_4N_4{}^-$). Elemental analysis: calculated for $C_{20}H_{36}BN_4P$: C, 64.18; H, 9.69; N, 14.97. Found C, 64.58; H, 10.17; N, 14.88.

[P(2O2)$_3$1][B(CN)$_4$] was prepared by metathesis from 5.68 g [P(2O2)$_3$1]I (14.5 mmol / 1.00 eq) and 2.73 g K[B(CN)$_4$] (17.7 mmol / 1.22 eq) in 200 mL CH_2Cl_2 and 20 mL H_2O. The organic phase was washed five times with 20 mL H_2O each, removal of the solvent by means of rotary evaporation followed by drying in high vacuum gave 5.13 g of [P(2O2)$_3$1][B(CN)$_4$] (13.5 mmol / 93%). ^1H NMR (DMSO−d_6, 400 MHz, δ in ppm): 3.74 (dt, $^3J_{H/P}$ = 19.5 Hz, $^3J_{H/H}$ = 6.1 Hz, 6H, P−CH_2−**CH_2**−O), 3.49 (q, $^3J_{H/H}$ = 7.0 Hz, 6H, P−$(CH_2)_2$−O−**CH_2**), 2.56 (dt, $^2J_{H/P}$ = 13.4 Hz, $^3J_{H/H}$ = 6.1 Hz, 6H, P−**CH_2**−CH_2−O), 1.87 (d, $^2J_{H/P}$ = 14.6 Hz,

3H, P−$\mathbf{CH_3}$), 1.15 (t, $^3J_{H/H}$ = 7.1 Hz, 9H, P−$(CH_2)_2$−O−CH_2−$\mathbf{CH_3}$); $^{13}C\{^1H\}$ NMR (DMSO−d_6, 101 MHz, δ in ppm): 121.78 (q, $^1J_{C/B}$ = 70.8 Hz, ^{11}B−**CN**), 121.78 (sep, $^1J_{C/B}$ = 23.7 Hz, ^{10}B−**CN**), 65.75 (s, P−$(CH_2)_2$−O−$\mathbf{CH_2}$), 62.69 (d, $^2J_{C/P}$ = 6.3 Hz, P−CH_2−$\mathbf{CH_2}$−O), 22.48 (d, $^1J_{C/P}$ = 50.8 Hz, P−$\mathbf{CH_2}$−CH_2−O), 14.69 (s, O−CH_2−$\mathbf{CH_3}$), 5.80 (d, $^1J_{C/P}$ = 51.3 Hz, P−$\mathbf{CH_3}$); $^{31}P\{^1H\}$ NMR (DMSO−d_6, 162 MHz, δ in ppm): 31.70 (s); ^{11}B NMR (DMSO−d_6, 128 MHz, δ in ppm): −38.52 (s). HRMS, ESI$^+$: m/z found 265.1937, calc. 265.1933 ($PC_{13}H_{30}O_3{}^+$); ESI$^-$: m/z found 115.0211, calc. 115.0216 ($^{11}BC_4N_4{}^-$). Elemental analysis: calculated for $C_{17}H_{30}BN_4O_3P$: C, 53.70; H, 7.95; N, 14.74. Found C, 54.13; H, 8.22; N, 14.58.

[P2228][NTf$_2$] was prepared by metathesis from 11.57 g [P2228]Br (37.2 mmol / 1.00 eq) and 12.16 g Li[NTf$_2$] (42.4 mmol / 1.14 eq) in 200 mL CH_2Cl_2 and 20 mL H_2O. The organic phase was washed two times with 20 mL H_2O each, removal of the solvent by means of rotary evaporation followed by drying in high vacuum gave 18.52 g of [P2228][NTf$_2$] (36.2 mmol / 97%). ^{1}H NMR (DMSO−d_6, 400 MHz, δ in ppm): 2.21 (dq, $^2J_{H/P}$ = 13.2 Hz, $^3J_{H/H}$ = 7.6 Hz, 6H, P−$\mathbf{CH_2}$−CH_3), 2.23-2.13 (m, 2H), 1.55-1.44 (m, 2H), 1.44-1.35 (m, 2H), 1.35-1.19 (m, 8H), 1.13 (dt, $3\,J_{H/P}$ = 18.1 Hz, $^3J_{H/H}$ = 7.6 Hz, 9H, P−CH_2−$\mathbf{CH_3}$), 0.90-0.83 (m, 3H); $^{13}C\{^1H\}$ NMR (DMSO−d_6, 101 MHz, δ in ppm): 119.51 (q, $1\,J_{C/F}$ = 321.7 Hz, $\mathbf{CF_3}$) 31.21 (s), 30.13 (d, $^3J_{C/P}$ = 14.9 Hz, P−CH_2−CH_2−$\mathbf{CH_2}$), 28.40 (s), 28.20 (s), 22.03 (s), 20.48 (d, $^2J_{C/P}$ = 4.4 Hz, P−CH_2−$\mathbf{CH_2}$−CH_2), 16.42 (d, $^1J_{C/P}$ = 47.6 Hz, P−$\mathbf{CH_2}$−CH_2−CH_2), 13.82 (s, −CH_2−CH_2−$\mathbf{CH_3}$), 10.53 (d, $^1J_{C/P}$ = 49.3 Hz, P−$\mathbf{CH_2}$−CH_3), 5.06 (d, $^2J_{C/P}$ = 5.32 Hz, P−CH_2−$\mathbf{CH_3}$); $^{31}P\{^1H\}$ NMR (DMSO−d_6, 162 MHz, δ in ppm): 38.90 (s); ^{19}F NMR (DMSO−d_6, 377 MHz, δ in ppm): −78.88 (s). HRMS, ESI$^+$: m/z found 231.2240, calc. 231.2242 ($PC_{14}H_{32}{}^+$); ESI$^-$: m/z found 279.9180, calc. 279.9173 ($C_2NO_4S_2F_6{}^-$).

[P222(2O2O2)][NTf$_2$] was prepared by metathesis from 8.21 g of [P222(2O2O2)]Br (26.1 mmol / 1.00 eq) and 9.34 g of lithium bis(trifluoromethanesulfonyl)imide (32.6 mmol / 1.25 eq) in a biphasic mixture of approximately 250 mL dichloromethane and 15 mL of H_2O. After 16 h of intense stirring, the organic phase was removed, the aqueous phase

extracted with approximately 100 mL CH_2Cl_2 and the combined organic phases extracted with approximately 20 mL water for three times. After drying over $MgSO_4$ and filtering, the solvent was removed by rotary evaporation and the residue dried in high vacuum for two days at 40°C with stirring. 13.0 g of the title compound (25.3 mmol / 93%) were obtained as colorless liquid. ^{1}H NMR ($CDCl_3$, 400 MHz, δ in ppm): 3.80 (dt, $^3J_{H/P}$ = 19.3 Hz, $^3J_{H/H}$ = 621 Hz, 2H, P−CH_2−$\mathbf{CH_2}$−O), 3.61-3.50 (m, 4H, O−$\mathbf{CH_2}$−$\mathbf{CH_2}$−O), 3.47 (q, $^3J_{H/H}$ = 7.0 Hz, 2H, O−$\mathbf{CH_2}$−CH_3), 2.46 (dt, $^2J_{H/P}$ = 11.9 Hz, $^3J_{H/H}$ = 5.9 Hz, P−$\mathbf{CH_2}$−CH_2−O), 2.23 (dq, $^2J_{H/P}$ = 13.1 Hz, $^3J_{H/H}$ = 7.7 Hz, 6H, P−$\mathbf{CH_2}$−CH_3), 1.15 (dt, 3 $J_{H/P}$ = 18.3 Hz, $^3J_{H/H}$ = 7.8 Hz, 9H, P−CH_2−$\mathbf{CH_3}$), 1.16 (t, $^3J_{H/H}$ = 7.0 Hz, 3H, O−CH_2−$\mathbf{CH_3}$); ^{13}C{^{1}H} NMR ($CDCl_3$, 101 MHz, δ in ppm): 119.96 (q, 1 $J_{C/F}$ = 321.5 Hz, 70.63 (s), 69.82 (s), 66.55 (s), 63.60 (d, $^2J_{C/P}$ = 7.8 Hz, P−CH_2−$\mathbf{CH_2}$−O), 19.25 (d, $^1J_{C/P}$ = 50.2 Hz, P−$\mathbf{CH_2}$−CH_2−O), 15.23 (s, −CH_2−CH_2−$\mathbf{CH_3}$), 12.55 (d, $^1J_{C/P}$ = 48.1 Hz, P−$\mathbf{CH_2}$−CH_3), 5.56 (d, $^2J_{C/P}$ = 5.57 Hz, P−CH_2−$\mathbf{CH_3}$); ^{19}F NMR ($CDCl_3$, 377 MHz, δ in ppm): −78.82 (s); ^{31}P{^{1}H} NMR ($CDCl_3$, 162 MHz, δ in ppm): 39.13 (s). HRMS, ESI$^+$: m/z found 235.1818, calc. 235.1827 ($PC_{12}H_{28}O_2{}^+$); ESI$^-$: m/z found 279.9174, calc. 279.9173 ($C_2NO_4S_2F_6{}^-$).

[P5551][NTf$_2$] was prepared by metathesis from 5.77 g [P5551]I (14.9 mmol / 1.00 eq) and 5.48 g Li[NTf$_2$] (19.1 mmol / 1.28 eq) in 200 mL CH_2Cl_2 and 20 mL H_2O. The organic phase was washed two times with 20 mL H_2O each, removal of the solvent by means of rotary evaporation followed by drying in high vacuum gave 7.76 g of [P5551][NTf$_2$] (14.4 mmol / 96%). ^{1}H NMR (DMSO−d_6, 400 MHz, δ in ppm): 2.22-2.06 (m, 6H, P−$\mathbf{CH_2}$−CH_2), 1.78 (d, $^2J_{H/P}$ = 14.0 Hz, 3H, P−$\mathbf{CH_3}$), 1.57-1.43 (m, 6H, P−CH_2−$\mathbf{CH_2}$), 1.43-1.25 (m, 12H, P−$(CH_2)_2$−$(\mathbf{CH_2})_2$), 0.90 (t, $^3J_{H/H}$ = 7.0 Hz, 9H, P−$(CH_2)_4$−$\mathbf{CH_3}$); ^{13}C{^{1}H} NMR (DMSO−d_6, 101 MHz, δ in ppm): 119.50 (q, 1 $J_{C/F}$ = 321.8 Hz, $\mathbf{CF_3}$), 32.13 (d, $^3J_{C/P}$ = 15.4 Hz, P−$(CH_2)_2$−$\mathbf{CH_2}$), 21.29 (s, P−CH_2−CH_2−CH_2−$\mathbf{CH_2}$), 20.19 (d, $^2J_{C/P}$ = 4.3 Hz, P−CH_2−$\mathbf{CH_2}$), 19.08 (d, $^1J_{C/P}$ = 49.0 Hz, P−$\mathbf{CH_2}$−CH_2), 13.41 (s, P−$(CH_2)_4$−$\mathbf{CH_3}$, 3.00 (d, $^1J_{C/P}$ = 51.7 Hz, P−$\mathbf{CH_3}$); ^{31}P{^{1}H} NMR (DMSO−d_6, 162 MHz, δ in ppm): 32.19 (s);

^{19}F NMR (DMSO−d$_6$, 377 MHz, δ in ppm): −78.95 (s). HRMS, ESI$^+$: m/z found 259.2551, calc. 259.2555 ($PC_{16}H_{36}{}^+$); ESI$^-$: m/z found 279.9173, calc. 279.9173 ($C_2NO_4S_2F_6{}^-$).

[P(2O2)$_3$1][NTf$_2$] was prepared by metathesis from 4.94 g [P(2O2)$_3$1]I (12.6 mmol / 1.00 eq) and 4.94 g Li[NTf$_2$] (17.2 mmol / 1.37 eq) in 200 mL CH_2Cl_2 and 20 mL H_2O. The organic phase was washed three times with 20 mL H_2O each, removal of the solvent by means of rotary evaporation followed by drying in high vacuum gave 6.71 g of [P(2O2)$_3$1][NTf$_2$] (12.3 mmol / 98%). ^{1}H NMR (DMSO−d$_6$, 400 MHz, δ in ppm): 3.73 (dt, $^3J_{H/P}$ = 19.3 Hz, $^3J_{H/H}$ = 6.2 Hz, 6H, P−CH$_2$−**CH$_2$**−O), 3.47 (q, $^3J_{H/H}$ = 7.0 Hz, 6H, P−(CH$_2$)$_2$−O−**CH$_2$**), 2.55 (dt, $^2J_{H/P}$ = 13.4 Hz, $^3J_{H/H}$ = 6.2 Hz, 6H, P−**CH$_2$**−CH$_2$−O), 1.87 (d, $^2J_{H/P}$ = 14.6 Hz, 3H, P−**CH$_3$**), 1.13 (t, $^3J_{H/H}$ = 7.0 Hz, 9H, P−(CH$_2$)$_2$−O−CH$_2$−**CH$_3$**); ^{13}C{^{1}H} NMR (DMSO−d$_6$, 101 MHz, δ in ppm): 119.52 (q, 1 $J_{C/F}$ = 321.9 Hz, **CF$_3$**), 65.67 (s, P−(CH$_2$)$_2$−O−**CH$_2$**), 62.67 (d, $^2J_{C/P}$ = 6.1 Hz, P−CH$_2$−**CH$_2$**−O), 22.40 (d, $^1J_{C/P}$ = 50.5 Hz, P−**CH$_2$**−CH$_2$−O), 14.72 (s, P−(CH$_2$)$_2$−O−CH$_2$−**CH$_3$**), 5.72 (d, $^1J_{C/P}$ = 51.2 Hz, P−**CH$_3$**); ^{31}P{^{1}H} NMR (DMSO−d$_6$, 162 MHz, δ in ppm): 31.44 (s); ^{19}F NMR (DMSO−d$_6$, 377 MHz, δ in ppm): −78.85 (s). HRMS, ESI$^+$: m/z found 265.1927, calc. 265.1933 ($PC_{13}H_{30}O_3{}^+$); ESI$^-$: m/z found 279.9177, calc. 279.9173 ($C_2NO_4S_2F_6{}^-$).

Experimental data

Glass Transitions and Density

DSC measurements were made using a DSC 1 STARe system (Mettler Toledo, Gießen, Germany) with liquid nitrogen cooling system. Approximately 10 mg of the samples were hermetically sealed in aluminum crucibles inside the glovebox to prevent uptake of ambient moisture. Slow scan rates of ±1 °C min^{-1} were used to avoid a quenching of the samples and a more accurate determination of the glass transition temperature T_g upon cooling to −120 °C. Glass transition temperatures T_g were obtained using the midpoint method.[3] The collected macroscopic properties of the ionic liquids are given in table S1. The general

physical chemical properties of the samples with the $[P(2O2)_31]^+$ and $[P5551]^+$ cations were already reported[1] and are given here for the sake of comparison with the other cations, the FFC and MD results.

Table S1: Overview of the physicochemical properties of the investigated ionic liquids.

Ionic liquid	T_g / °C	ρ (25 °C) / g mL^{-1}	η (25 °C) / mPa · s	Λ_M (25 °C) / S · cm^2 · mol^{-1}
$[P222(2O2O2)][NTf_2]$	−92	1.3201	39.92	1.037
$[P2228][NTf_2]$	−84	1.2413	111.4	0.337
$[P222(2O2O2)][B(CN)_4]$	−88	0.9951	34.06	1.338
$[P2228][B(CN)_4]$	−87	0.9314	72.08	0.640
$[P(2O2)_31][NTf_2]$	−91[a)]	1.2925	37.75	1.300
$[P5551][NTf_2]$	−81[b)]	1.2018	206.3	0.178
$[P(2O2)_31][B(CN)_4]$	−83	0.9948	28.19	1.739
$[P5551][B(CN)_4]$	−84	0.9147	147.7	0.390

[a)] Additional cold crystallization temperature T_{cc} = −52 °C and melting temperature T_m = −30 °C observed upon heating the sample in the DSC.
[b)] T_{cc} = −47 °C and T_m = 20 °C observed upon heating.

For all the investigated samples only glass transitions are observed in the range from 181 to 192 K. The formation of glasses at temperatures only below 192 K ensures that in the temperature range of the FFC experiments, the relaxation pathways outlined in the theory section are valid for the ionic liquids investigated in this study. This means that the Redfield limit can be expected to be fulfilled for the relaxation experiments conducted in this study. The lowest values of T_g for each cation set with similar side chain length are found for the ether ionic liquids with $[NTf_2]^-$ anion, the highest for the alkylated samples with this anion. While the $[NTf_2]^-$ samples have differences of 8 and 10 K the glass transition temperature of the $[B(CN)_4]^-$ are only 1 K apart. The two $[PRRR1][NTf_2]$ ionic liquids show additional crystallization (cold crystallization T_{cc}) and melting T_m events in the heating traces.

For the density measurements a pycnometer of 5 mL nominal volume (Neubert Glas, Geschwenda, Germany) was placed in the bath of a Proline RP 1845 thermostat (LAUDA, Lauda-Königshofen, Germany). The volume of the pycnometer at different temperatures

was calibrated with n-octane and pure water. Experimental densities are given in table S2. The densities were fitted using the linear equation S1. The obtained fitting results are given in table S3.

$$\rho = a \cdot \frac{T}{\mathrm{K}} + b \tag{S1}$$

Table S2: Experimental density values for the ionic liquids at stated temperatures given in g/mL.

	Temperature / ° C							
Ionic liquid	25	35	45	55	65	75	85	95
[P222(2O2O2)][NTf_2]	1.3201	1.3105	1.3018	1.2924	1.2837	1.2744	1.2656	1.2570
[P2228][NTf_2]	1.2413	1.2331	1.2247	1.2155	1.2081	1.1990	1.1910	1.1823
[P222(2O2O2)][$B(CN)_4$]	0.9951	0.9879	0.9808	0.9736	0.9663	0.9592	0.9523	0.9455
[P2228][$B(CN)_4$]	0.9314	0.9249	0.9183	0.9116	0.9051	0.8984	0.8921	0.8856
[$P(2O2)_3 1$][NTf_2]	1.2925	1.2840	1.2751	1.2661	1.2571	1.2481	1.2401	1.2322
[P(5551)][NTf_2]	1.2018	1.1935	1.1855	1.1774	1.1695	1.1614	1.1541	1.1456
[$P(2O2)_3 1$][$B(CN)_4$]	0.9948	0.9876	0.9806	0.9732	0.9664	0.9587	0.9523	0.9448
[P(5551)][$B(CN)_4$]	0.9147	0.9095	0.9030	0.8967	0.8907	0.8853	0.8779	0.8721

Table S3: Fitting parameters for the experimental density data following equation S1.

Ionic liquid	a /$\mathrm{g \cdot mL^{-1} \cdot K^{-1}}$	Δa / 10^{-3}	b / $10^{-4}\mathrm{g \cdot mL^{-1}}$	Δb /10^{-6}	R^2
[P222(2O2O2)][NTf_2]	−9.019	4.13	1.589	1.38	0.9999
[P2228][NTf_2]	−8.433	5.35	1.493	1.79	0.9998
[P222(2O2O2)][$B(CN)_4$]	−7.112	2.90	1.207	0.97	0.9999
[P2228][$B(CN)_4$]	−6.556	1.74	1.127	0.58	1.0000
[$P(2O2)_3 1$][NTf_2]	−8.716	7.63	1.552	2.55	0.9995
[P(5551)][NTf_2]	−7.991	3.77	1.440	1.26	0.9999
[$P(2O2)_3 1$][$B(CN)_4$]	−7.132	3.81	1.207	1.27	0.9998
[P(5551)][$B(CN)_4$]	−6.132	7.14	1.098	2.38	0.9992

All ionic liquids containing ether functionalities have higher densities than the corresponding analogues with methylene groups. This effect of the higher density is slightly more pronounced for the samples with three ether groups compared to ionic liquids with the $[P222(2O2O2)]^+$ cation. Regarding the anions, higher differences in the density ratio between ether and hydrocarbon cation are found for the samples with $[B(CN)_4]^-$.

The observation of only glass transitions in the DSC curves for ionic liquids is a common finding in the literature.[3] The commonly observed low melting transitions, characteristic of the class of ionic liquids, are the result of their highly destabilized crystal structures and analogous structural organization in liquid and crystalline state. The thermodynamic reason for the low melting points are the lower lattice enthalpies compared to conventional organic salts and high differences in entropy for the solid and liquid state. These effects results from the structure of the ions, as ionic liquids are, by design, made from asymmetric, charge delocalized, and flexible ions which do not pack well into a crystal lattice.[4] Combined with kinetic aspects, this often leads to pronounced supercooling, *i.e.* the absence of crystalline phases under the experimental conditions along with the occurrence of only glass transitions as observed for the ionic liquids in this study. The high entropy of melting is the result of the increase in configurational and conformational entropy upon transition from crystalline solid to disordered liquid. Thereby, the configurational entropy is the result of the possible ion arrangements in liquids state and corresponds to the different energetically accessible ion coordinations. It is often assumed that the ether functionalization significantly increases the conformational flexibility of cation side chains, and it is therefore reasonable to expect a marked decrease in the glass transition temperatures for ionic liquids with ether substituted cations. This is indeed the case when comparing the samples with the $[NTf_2]^-$ anion where the ether cations have T_g values which are 8 to 10 K lower than the alkylated analogs. Nonetheless, the samples with the $[B(CN)_4]^-$ anion showed only 1 K difference upon ether functionalization of the cation. Therefore, it seems reasonable to assume that either the gain in conformational entropy upon ether functionalization contributes less for the $[B(CN)_4]^-$ ionic liquids, or that the configurational entropy has a higher contribution in case of the ether substituted $[NTf_2]^-$ ionic liquids. The latter rationalization is supported by the observation that the different conformers of the imide anion also contribute to the (configurational) entropy.[5] Although the $[NTf_2]^-$ ionic liquids with purely alkylated cations have more conformational flexibility through the cis and trans isomerization, the glass tran-

sition temperatures of their counterparts with the $[B(CN)_4]^-$ anion are lower. However, the situation is reversed for the ether substituted samples. The experimental results overall indicate that the configurational entropy is the dominating factor for the glass transitions and that the conformational flexibility of the ions plays a minor role. The MD simulations for the $[NTf_2]^-$ ionic liquids further support the experimental findings by showing that in case of the ether substituted ionic liquids, the anion coordination is significantly more diffuse, *cf.* the discussion of spatial distribution functions below. The higher degree of freedom for the cation-anion coordination corresponds to an marked increase in configurational entropy as a result of the altered cation conformation upon ether functionalization of the side chains.

The higher densities of the $[NTf_2]^-$ ionic liquids compared to their $[B(CN)_4]^-$ complements can be clearly explained by the higher atomic weight of the elements constituting the imide anion. This interpretation cannot be applied to differences in density upon replacing methylene units with ether groups as the resulting cations have similar molecular weights and ion volumes. However, the higher densities of the ether ionic liquids can be well rationalized by a change in cation conformations upon ether functionalization. Is has been reported previously that ionic liquids with ether chains form more compact cations as a result of the curling of the ether chains towards the positively charged cation center.[1,6] As significantly higher densities are found for all ether samples, the curled, compact structures seem to be formed by all ether functionalized cations, independent of the anion.

Viscosity

Experimental values for the viscosity η are given in table S4. The resulting fitting parameters for the Vogel-Fulcher-Tammann equation S2 are given in table S5.

$$\eta = \eta_0 \exp\left(\frac{B}{T - T_0}\right) \tag{S2}$$

From the VFT fitting parameters for a particular transport property, Angell's strength

factor $|\delta = B/T_0|$ can be calculated.[7,8] Note that Angell's strength factor is usually termed D in the literature, which we did not use here to avoid confusing with the diffusion coefficients, also named D. δ is a measure for the deviation from Arrhenius-type behavior. δ is related to the kinetic fragility index m by $m = 16 + 590/\delta$.[7,8]. Low values of Angell's strength factor indicate highly fragile liquids, such as it is observed for the ionic liquids in this set. The values obtained of Angell's strength factor for viscosity and molar conductivity are quite similar and differ by -11% to $+12\%$. All cation with the ether-substitutions have lower δ-values, thus higher fragilities than the alkylated cations for a common cation. For the same cation the ionic liquids with the $[B(CN)_4]^-$ anion have higher fragilities than the ones with the $[NTf_2]^-$. The low values for the Angell strength factors δ_x, *i.e.* the high fagilities, are a common finding for ionic liquids and indicate a quite strong deviation from the Arrhenius-dependence of the transport properties, and thus pronounced T-dependent activation energies for the transport properties. The values show a clear trend towards higher fragilities for the ionic liquids with ether groups and for the $[B(CN)_4]^-$ anions. Literature findings show a trend for fragility increasing with conformational flexibility of the ions and decreasing for elongated alkyl side chains. The effect of the conformational flexiblity of the anions is for instance seen when for prototypical ionic liquids with a common cation, *e.g.* the 1-butyl-3-methyl imidazolium $[C_4C_1im]^+$ paried with the rigid anions tetrafluoroborate $[BF_4]^-$, $\delta_\eta([C_4C_1im][BF_4]) = 5.92$,[9] or hexafluorophosphate $[PF_6]^-$, $\delta_\eta([C_4C_1im][PF_6]) = 6.96$,[10] to the flexible $[Ntf_2]^-$ $\delta_\eta([C_4C_1im][NTf_2]) = 4.65$.[11] Elongation of alkyl side chains in the 1-alkyl-3-methyl-imidazolium cation $[C_xC_1im]^+$ leads to lower fragilities of the resulting liquids, as exemplified for the $[PF_6]^-$ anion, where an increase from butyl ($x = 4$) $\delta_\eta([C_4C_1im][PF_6]) = 6.96$,[10] over hexyl ($x = 6$), $\delta_\eta([C_6C_1im][PF_6]) = 7.81$,[11] to octyl ($x = 8$), δ_η $([C_8C_1im][Pf_6]) = 8.91$,[10] can be observed. Overall the trend towards higher fragilities for more flexible ions, as found in the literature, is also observed here when comparing the ether substituted cation with the fully alkylated ones. However, this trend is not found for the anions as the more flexible imides show higher Angell strength factors than the tetra-

cyanoborates when paired with the same cation. The "flexibility" in ionic liquids with the $[B(CN)_4]^-$ anion presumably stems from the aggregation behaviour observed in the MD simulation. In other words, the reorganization within anionic aggregates (which are not favourable for $[NTf_2]^-$) could impart $[B(CN)_4]^-$ anions with a "intermolecular conformational flexibility". The effect of increasing fragility with increasing alkyl chain length is only slightly pronounced when comparing the $[P222X]^+$ to the $[PXXX1]^+$ samples. Thereby, the triethylphosphonium samples, which have shorter side chains, when taking the length of all attached groups into account, have higher fragilities for the $[NTf_2]^-$ and alkylated $[B(CN)_4]^-$ samples. Due to the interrelation of the transport properties by the Walden relation S3,

$$\Lambda_{\mathrm{M}} = (\eta^{-1})^t, \tag{S3}$$

the values for the fragilities/δ_x are quite similar. The ether substituted ionic liquids have significantly higher fragilities than prototypical alkylated ionic liquids. Correlations between the entropy and therefore structure of liquids and the kinetic fragility have been pointed out in the past.[12] The tetracyanoborates therefore seem to undergo more distinct changes in their liquids structure upon approaching T_{g}.

Table S4: Experimental viscosity values for the ionic liquids at stated temperatures given in mPa · s.

Temperature T /° C	Ionic liquid							
	[P222(2O2O2)] [NTf_2]	[P2228] [NTf_2]	[P222(2O2O2)] [$B(CN)_4$]	[P2228] [$B(CN)_4$]	[$P(2O2)_3 1$] [NTf_2]	[P(5551)] [NTf_2]	[$P(2O2)_3 1$] [$B(CN)_4$]	[P(5551)] [$B(CN)_4$]
25	32.75	111.35	34.06	72.08	32.75	206.28	28.19	147.70
30	26.83	86.31	27.84	56.75	26.83	154.88	23.02	111.50
35	22.28	67.93	23.13	45.59	22.28	118.54	19.14	85.96
40	18.72	54.29	19.42	36.97	18.72	92.36	16.12	67.54
45	15.92	44.00	16.59	30.50	15.92	73.05	13.78	53.83
50	13.69	36.16	14.31	25.46	13.69	58.66	11.89	43.58
55	11.87	30.15	12.41	21.50	11.87	47.78	10.38	35.77
60	10.40	25.36	10.87	18.33	10.40	39.33	9.13	29.68
65	9.17	21.58	9.60	15.75	9.17	32.71	8.08	24.96
70	8.15	18.55	8.56	13.69	8.15	27.49	7.22	21.17
75	7.28	16.05	7.65	12.00	7.28	23.32	6.49	18.15
80	6.57	14.02	6.88	10.55	6.57	19.98	5.88	15.71
85	5.94	12.31	6.23	9.35	5.94	17.23	5.35	13.70
90	5.41	10.89	5.68	8.38	5.41	15.01	4.88	12.04
95	4.93	9.69	5.19	7.52	4.93	13.13	4.48	10.68
100	4.52	8.67	4.76	6.79	4.52	11.57	4.14	9.49
105	4.17	7.82	4.39	6.17	4.17	10.27	3.84	8.50

Table S5: Fitting parameters for the experimental viscosity data using VFT equation S2.

Ionic liquid	η_0 / 10^{-2} mPa · s	$\Delta\eta$ / 10^{-4}	B / K	ΔB	T_0 / K	ΔT_0 / 10^{-1}	R^2	δ_η [a)]
[P222(2O2O2)][NTf_2]	16.62	47.2	688.5	8.23	172.6	8.59	0.99999	3.99
[P2228][NTf_2]	9.44	33.4	937.2	10.5	165.7	8.32	1	5.66
[P222(2O2O2)][$B(CN)_4$]	17.05	46.7	674.3	8.06	170.8	8.73	0.99999	3.95
[P2228][$B(CN)_4$]	10.81	24.7	853.4	6.78	166.9	5.89	1	5.11
[$P(2O2)_3 1$][NTf_2]	14.54	16.6	705.5	3.48	167.9	3.73	0.99999	4.20
[P(5551)][NTf_2]	5.928	5.86	1119	3.05	161.0	2.10	1	6.95
[$P(2O2)_3 1$][$B(CN)_4$]	20.95	34.3	573.3	4.56	181.2	5.47	0.99999	3.16
[P(5551)][$B(CN)_4$]	9.17	19.6	932.5	6.17	171.9	4.73	1	5.43

[a)] Angell strength parameter $|\delta = B/T_0|$, with B and T_0 obtained from the Vogel-Fulcher-Tammann fit, as a measure for kinetic fragility.

Specific conductivity

Experimental values for the specific conductivity κ as obtained from impedance spectroscopy are given in table S6. The resulting fitting parameters for the Vogel-Fulcher-Tammann equation S4 are given in table S7.

$$\kappa = \kappa_0 \exp\left(\frac{B}{T - T_0}\right) \tag{S4}$$

Again, for a common cation the ionic liquids with the $[B(CN)_4]^-$ anion show higher fragilities, thus lower δ_κ values, than the ones with the $[NTf_2]^-$.

Table S6: Experimental values for the specific conductivity κ of the ionic liquids at the specified temperatures given in $mS \cdot cm^{-1}$.

Temperature T /° C	Ionic liquid							
	[P222(2O2O2)] [NTf$_2$]	[P2228] [NTf$_2$]	[P222(2O2O2)] [B(CN)$_4$]	[P2228] [B(CN)$_4$]	[P(2O2)$_3$1] [NTf$_2$]	[P(5551)] [NTf$_2$]	[P(2O2)$_3$1] [B(CN)$_4$]	[P(5551)] [B(CN)$_4$]
25	2.498	0.869	3.802	1.722	3.080	0.396	4.548	0.954
30	3.028	1.104	4.559	2.146	3.685	0.513	5.418	1.226
35	3.625	1.377	5.383	2.631	4.353	0.658	6.391	1.547
40	4.275	1.688	6.292	3.185	5.076	0.827	7.491	1.916
45	4.985	2.049	7.287	3.806	5.835	1.027	8.608	2.337
50	5.734	2.453	8.350	4.486	6.700	1.261	9.814	2.827
55	6.561	2.922	9.475	5.226	7.616	1.519	11.11	3.383
60	7.449	3.426	10.68	6.038	8.578	1.819	12.48	4.001
65	8.391	3.992	11.96	6.925	9.587	2.157	13.90	4.681
70	9.374	4.598	13.30	7.886	10.66	2.537	15.39	5.402
75	10.43	5.251	14.69	8.894	11.80	2.961	16.91	6.202
80	11.53	5.938	16.13	9.969	12.95	3.420	18.44	7.058
85	12.67	6.699	17.65	11.11	14.15	3.916	20.07	7.975
90	13.90	7.518	19.19	12.31	15.40	4.437	21.61	8.952
95	15.10	8.390	20.81	13.56	16.67	5.013	23.28	9.941
100	16.37	9.293	22.49	14.85	17.99	5.614	24.92	11.08

Table S7: Fitting parameters for the experimental specific conductivity κ using VFT equation S4.

Ionic liquid	κ_0 / $mS \cdot cm^{-1}$	$\Delta\kappa_0$	B / K	ΔB	T_0 / K	ΔT_0	R^2	δ_κ [a)]
$[P222(2O2O2)][NTf_2]$	416.5	10.2	-661.0	8.55	168.9	1.14	0.99999	3.91
$[P2228][NTf_2]$	582.9	24.3	-852.1	14.9	167.3	1.60	0.99999	5.09
$[P222(2O2O2)][B(CN)_4]$	462.3	6.34	-612.7	4.71	170.5	0.67	1	3.59
$[P2228][B(CN)_4]$	544.1	8.65	-720.6	5.45	173.0	0.66	1	4.17
$[P(2O2)_31][NTf_2]$	370.8	8.57	-615.1	7.96	169.8	1.13	0.99999	3.62
$[P(5551)][NTf_2]$	567.8	41.8	-942.8	26.4	168.8	2.57	0.99998	5.58
$[P(2O2)_31][B(CN)_4]$	300.2	10.2	-457.3	10.3	189.3	1.71	0.99998	2.42
$[P(5551)][B(CN)_4]$	573.8	29.1	-770.8	17.1	177.9	1.91	0.99998	4.33

[a)] Angell strength parameter $|\delta = B/T_0|$, with B and T_0 obtained from the Vogel-Fulcher-Tammann fit, as a measure for kinetic fragility.

Molar conductivity

Calculated values for the molar conductivity Λ_M are listed in table S8. The obtained parameters for the fitting using Vogel-Fulcher-Tammann equation S5 are given in table S9.

$$\Lambda_M = \Lambda_{M,0} \exp\left(\frac{B}{T - T_0}\right) \tag{S5}$$

As for viscosity and specific conductivity the samples with the $[B(CN)_4]^-$ anion have higher fragilities than the ones with the $[NTf_2]^-$ and the same cation.

Table S8: Calculated values for the molar conductivity Λ_M of the ionic liquids at the specified temperatures given in $S \cdot cm^1 \cdot mol^{-1}$.

Temperature T	Ionic liquid							
/° C	[P222(2O2O2)] [NTf_2]	[P2228] [NTf_2]	[P222(2O2O2)] [$B(CN)_4$]	[P2228] [$B(CN)_4$]	[$P(2O2)_3 1$] [NTf_2]	[P(5551)] [NTf_2]	[$P(2O2)_3 1$] [$B(CN)_4$]	[P(5551)] [$B(CN)_4$]
25	1.037	0.337	1.338	0.640	1.300	0.178	1.739	0.390
30	1.262	0.429	1.610	0.801	1.561	0.231	2.078	0.503
35	1.516	0.538	1.908	0.985	1.850	0.297	2.461	0.637
40	1.793	0.661	2.238	1.197	2.164	0.375	2.895	0.791
45	2.099	0.805	2.602	1.435	2.497	0.467	3.338	0.969
50	2.422	0.967	2.992	1.698	2.876	0.576	3.820	1.176
55	2.781	1.156	3.408	1.985	3.281	0.696	4.339	1.412
60	3.168	1.360	3.857	2.302	3.708	0.836	4.891	1.676
65	3.581	1.591	4.335	2.649	4.159	0.995	5.469	1.967
70	4.015	1.839	4.837	3.028	4.639	1.175	6.078	2.278
75	4.482	2.107	5.363	3.427	5.156	1.375	6.706	2.625
80	4.973	2.391	5.910	3.856	5.678	1.594	7.336	2.997
85	5.486	2.707	6.489	4.313	6.224	1.831	8.017	3.399
90	6.038	3.049	7.085	4.795	6.796	2.083	8.662	3.828
95	6.583	3.415	7.711	5.303	7.386	2.361	9.369	4.266
100	7.162	3.796	8.366	5.830	7.999	2.654	10.07	4.772

Table S9: Fitting parameters for the molar conductivity Λ_M using VFT equation S5

Ionic liquid	$\Lambda_{\mathrm{M},0}$ / $\mathrm{mS \cdot cm^{-1}}$	$\Delta\Lambda_{\mathrm{M},0}$	B / K	ΔB	T_0 / K	ΔT_0	R^2	$\delta_{\Lambda_\mathrm{M}}$ [a)]
$[P222(2O2O2)][NTf_2]$	228.3	6.06	-725.8	9.54	163.5	1.20	0.99999	4.44
$[P2228][NTf_2]$	300.9	13.7	-920.1	16.7	162.8	1.71	0.99999	5.65
$[P222(2O2O2)][B(CN)_4]$	217.7	3.79	-679.9	6.21	164.6	0.82	1	4.13
$[P2228][B(CN)_4]$	268.5	3.96	-784.9	5.22	168.2	0.60	1	4.67
$[P(2O2)_31][NTf_2]$	205.3	4.50	-677.6	7.81	164.3	1.04	1	4.12
$[P(5551)][NTf_2]$	334.0	24.5	-1005	26.9	165.2	2.52	0.99998	6.09
$[P(2O2)_31][B(CN)_4]$	148.1	4.85	-508.1	10.3	184.1	1.61	0.99998	2.76
$[P(5551)][B(CN)_4]$	305.1	16.1	-828.2	18.2	174.0	1.94	0.99999	4.76

a) Angell strength parameter $|\delta = B/T_0|$, with B and T_0 obtained from the Vogel-Fulcher-Tammann fit, as a measure for kinetic fragility.

FFC

It should be outlined that equation 1 in the main paper is limited to the description of dipolar coupling between spins of the same type, but the like spin case is not valid to explain the dipolar interaction between ^{1}H and ^{19}F spins. To describe dipolar coupling between ^{1}H and ^{19}F, the Redfield approach in the so-called S-spin equilibrium limit can be used which provides the following relaxation equation:[13–15]

$$\frac{1}{T_1} = \frac{1}{60\pi}(\mu_0\gamma_I\gamma_S\hbar)^2 S(S+1)\left[J(\omega_I-\omega_S)+3J(\omega_I)+6J(\omega_I+\omega_S)\right], \tag{S6}$$

where γ_S is the gyromagnetic ratio of spins of the type S, S is the spin quantum number of those spins and ω_S the Larmor frequency at the relaxation field strength. One advantage of the Redfield treatment is that for the like-spin as well as the S-spin equilibrium limit the same spectral densities can be used, which means that the expressions 4 to 6 from the main paper remain valid in combination with the relaxation equation S6.[13,15] However, the work of Kruk *et al.* provides evidence that in ionic liquids with a significant higher hydrogen than fluorine density, the relaxation contribution due to the dipolar interaction between ^{1}H and ^{19}F is negligible and the relaxation behaviour can be solely assigned to dipolar coupling

between different ^{1}H-spins.[13] It will be shown later, that this also holds for the $[NTf_2]^-$ ionic liquids studied in this paper. Hence, the NMR dispersion of all investigated samples can be fully described in the Redfield like-spin limit in combination with the spectral densities outlined in the theory section of the main paper.

The influence of dipolar coupling between ^{1}H and ^{19}F spins on the overall relaxation was investigated using the S-spin equilibrium relaxation expression S6 in combination with the spectral densities as it is outlined in the theory section of the main paper. The resulting

(a) $[P5551]^+$ (b) $[P(2O2)_31]^+$

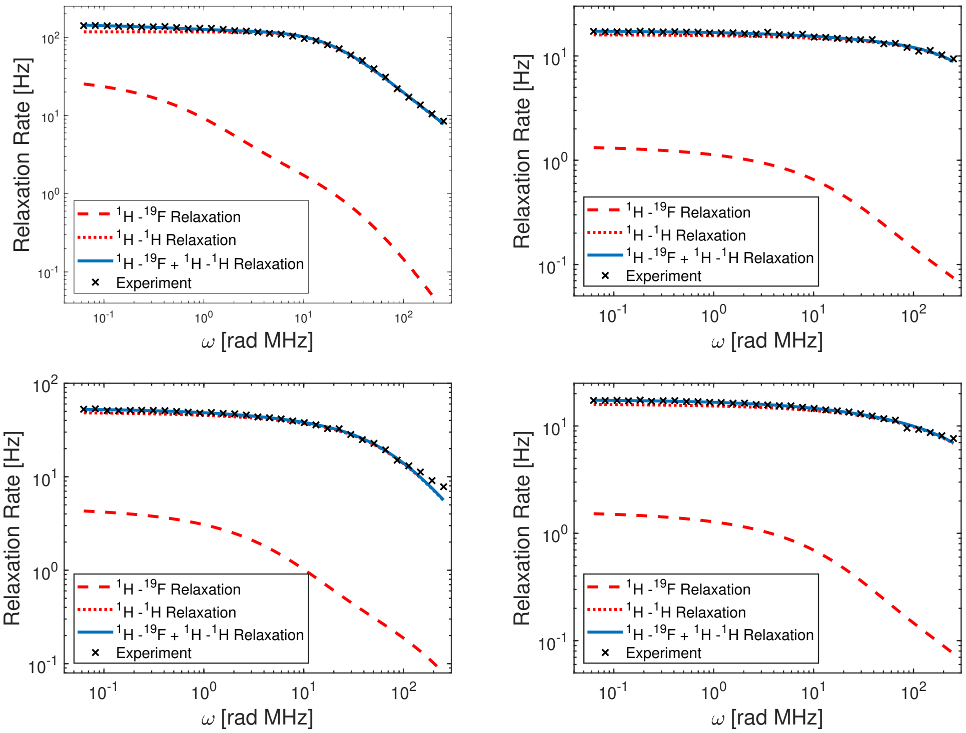

(c) $[P2228]^+$ (d) $[P2222O2O2]^+$

Figure S1: NMR dispersion of the investigated $[NTf_2]^-$ ionic liquids at 283 K. The (x)-markers correspond to the experimental values, whereas the full model fit is given by the solid lines (—). and its decomposition in ^{1}H-^{1}H and ^{1}H-^{19}F relaxation is represented by the dotted (· · ·) and dashed lines (−−) respectively.

overall fits and their decomposition into ^{1}H-^{1}H- and ^{1}H-^{19}F-dipolar coupling are displayed in figure S1. For visual clarity, the latter figure only shows the NMR dispersion at 283 K

but the results are generally valid across the whole observed temperature range. From figure S1, it becomes evident that the relaxation contribution due to dipolar coupling between ^{1}H and ^{19}F is small compared to the ^{1}H-^{1}H relaxation term. In more detail, in the low frequency regime, neglecting ^{1}H and ^{19}F dipolar coupling results in an error of less than 10 %, which decreases significantly with increasing field strength. In the high frequency regime, the error nearly vanishes to a relative value of less than 1 %. Overall, those findings agree with the results reported by Kruk *et al.* and show that the ^{1}H-^{19}F relaxation contribution can be neglected.[13] Another argument in favour of neglecting dipolar coupling between ^{1}H- and ^{19}F-spins is that the incorporation of this contribution results in at least one additional fitting parameter, which makes it more challenging to obtain reliable quantitative results from the model fits. In our analysis of data presented in this paper, we could observe that a full model fit including the ^{1}H-^{19}F relaxation contribution is far less numerically stable compared to the case when only ^{1}H-^{1}H dipolar coupling is considered. In fact, the obtained fitting parameters were strongly dependent on the chosen starting parameters, when the full model fit including the ^{1}H-^{19}F relaxation contribution was employed. Hence, for the following analysis presented in this paper, the ^{1}H-^{19}F relaxation contribution was ignored and the whole relaxation process was solely attributed to dipolar coupling between ^{1}H-spins.

The from the model fits obtained rotational and translational correlation times are listed in table S10 and S11. The resulting Arrhenius plots are displayed in figure S5 and S6.

Table S10: Rotational correlation times obtained from the model fits of the NMRD profiles. All correlation times are given in ns.

Temperature [K]	[P5551][NTf_2]	[$P(2O2)_31$][NTf_2]	[P5551][BCN_4]	[$P(2O2)_31$][BCN_4]	[P2228][NTf_2]	[P222(2O2O2)][NTf_2]	[P2228][BCN_4]	[P222(2O2O2)][BCN_4]
243	-	7.21*	-	8.13	-	10.77	-	4.04
253	-	5.26	-	3.39	3.89	3.46	1.63	1.29
263	12.84	2.28	5.59	1.16	1.96	1.39	0.84	0.46
273	7.60	0.90	3.18	0.61	1.22	0.59	0.54	0.18
283	4.58	0.54	1.54	0.33	0.76	0.33	0.29	0.10
295	2.19	-	0.47	-	0.38	-	0.11	-
303	1.43	-	0.25	-	-	-	-	-
313	0.85	-	0.10	-	-	-	-	-

* : $T = 248\,\mathrm{K}$, because the sample became solid during the FFC experiment, when cooled to 243 K.

Table S11: Translational correlation times obtained from the model fits of the NMRD profiles. All correlation times are given in ns.

Temperature [K]	[P5551][NTf_2]	[P(2O2)$_3$1][NTf_2]	[P5551][BCN_4]	[P(2O2)$_3$1][BCN_4]	[P2228][NTf_2]	[P222(2O2O2)][NTf_2]	[P2228][BCN_4]	[P222(2O2O2)][BCN_4]
243	-	100.56*	-	138.65	-	244.33	-	160.38
253	-	65.210	-	54.74	286.88	81.30	194.24	48.95
263	259.05	25.96	125.94	16.93	115.30	31.32	72.47	17.25
273	100.49	10.57	62.62	8.50	49.83	14.45	31.04	8.43
283	52.03	6.42	30.81	4.99	28.40	8.86	14.64	5.37
295	22.86	-	15.20	-	13.79	-	7.47	-
303	13.60	-	9.79	-	-	-	-	-
313	7.94	-	6.71	-	-	-	-	-

* : $T = 248\,\mathrm{K}$, because the sample became solid during the FFC experiment, when cooled to 243 K.

Table S12: Stretching parameters β obtained as described in the FFC fitting section of the main manuscript.

Temperature [K]	[P5551][NTf_2]	[P(2O2)$_3$1][NTf_2]	[P5551][BCN_4]	[P(2O2)$_3$1][BCN_4]	[P2228][NTf_2]	[P222(2O2O2)][NTf_2]	[P2228][BCN_4]	[P222(2O2O2)][BCN_4]
243	-	0.61*	-	0.51	-	0.43	-	0.40
253	-	0.71	-	0.40	0.23	0.25	0.26	0.22
263	0.24	0.75	0.19	0.29	0.24	0.22	0.27	0.17
273	0.23	0.72	0.17	0.42	0.26	0.24	0.35	0.18
283	0.23	0.63	0.13	0.51	0.32	0.40	0.37	0.31
295	0.24	-	0.10	-	0.33	-	0.40	-
303	0.29	-	0.11	-	-	-	-	-
313	0.33	-	0.10	-	-	-	-	-

* : $T = 248\,\mathrm{K}$, because the sample became solid during the FFC experiment, when cooled to 243 K.

The ratios τ_{trans}/τ_{rot} are given in table S13. The ratios τ_{trans}/τ_{rot} show a mostly temperature dependent behaviour and therefore indicate that the Stokes-Einstein-Debye relation is not valid.[16]

Table S13: τ_{trans}/τ_{rot} ratios.

Temperature [K]	[P5551][NTf_2]	[P(2O2)$_3$1][NTf_2]	[P5551][BCN_4]	[P(2O2)$_3$1][BCN_4]	[P2228][NTf_2]	[P222(2O2O2)][NTf_2]	[P2228][BCN_4]	[P222(2O2O2)][BCN_4]
243	-	13.95*	-	17.06	-	22.70	-	39.71
253	-	12.41	-	16.14	73.77	23.49	119.40	37.83
263	20.17	11.37	22.53	14.59	58.76	22.50	86.23	37.90
273	13.23	11.78	19.69	14.06	40.90	24.67	57.85	46.62
283	11.36	11.95	20.05	15.11	37.29	26.27	50.20	53.44
295	10.44	-	32.41	-	36.73	-	65.26	-
303	9.54	-	39.11	-	-	-	-	-
313	9.31	-	66.10	-	-	-	-	-

* : $T = 248\,\mathrm{K}$, because the sample became solid during the FFC experiment, when cooled to 243 K.

From Kruk *et al.* for instance, it is known that the low-frequency regime of the NMR dispersion can be approximated through the following linear equation:[13]

$$R_1 = R_1(0) - N\pi \left(\frac{\mu_0}{4\pi}\gamma_I^2\hbar\right)^2 \left(\frac{\sqrt{2}+8}{15}\right) D^{-\frac{3}{2}}\sqrt{\omega}, \tag{S7}$$

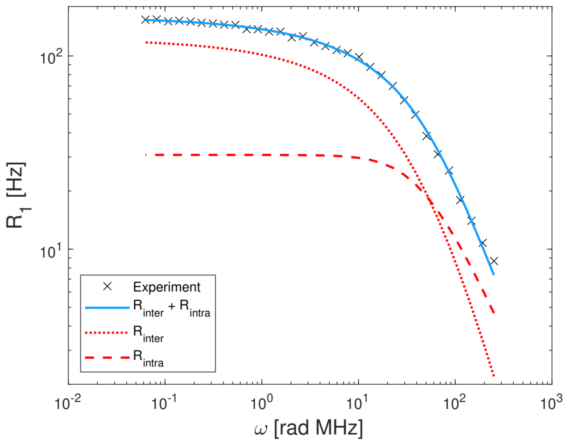

Figure S2: Spectral decomposition of the NMR dispersion of [P5551][$B(CN)_4$] at 273 K.

(a) Alkyl/Ether (b) [$N(Tf)_2$]/[$B(CN)_4$] (c) Short/Long

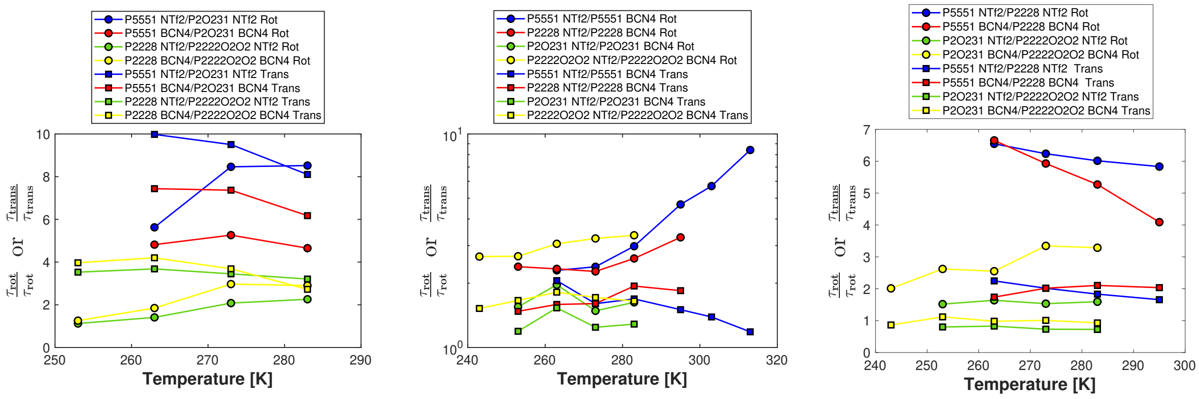

Figure S3: $\tau_{trans}/\tau_{trans}$ and τ_{rot}/τ_{rot} ratios as a function of temperature.

where R_{intra} is included in the constant $R_1(0)$ as it can be treated as frequency-independent in the low frequency regime. From this D can be approximated through a linear fit of low-frequency part of a NMRD profile. An example for this procedure is given in figure S4.

For this example, the value of D is $3.759 \cdot 10^{-12} \frac{\mathrm{m}^2}{\mathrm{s}}$, which is in reasonable agreement with the results from MD simulations and PGSTE measurements (compare table S16). From the relation $D = \frac{d^2}{2\tau_{trans}}$, τ_{trans} is estimated to 16.14 ns, which is in excellent agreement with the value obtained from the full NMRD profile fit (for d compare table S23, for τ_{trans} compare table S11).

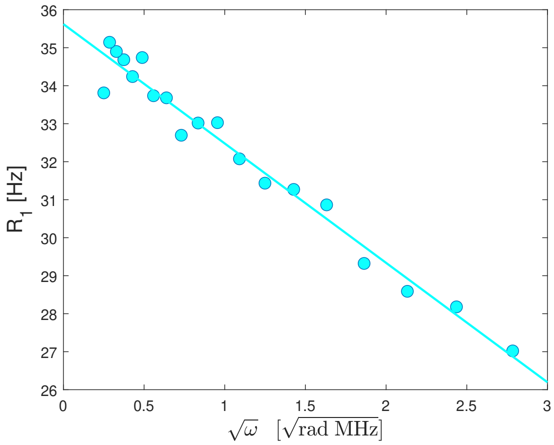

Figure S4: Linear fit of the low-frequency regime of a NMRD profile for the example of $[P5551][B(CN)_4]$ at 295 K.

(a) $[P5551]^+$ (b) $[P(2O2)_31]^+$

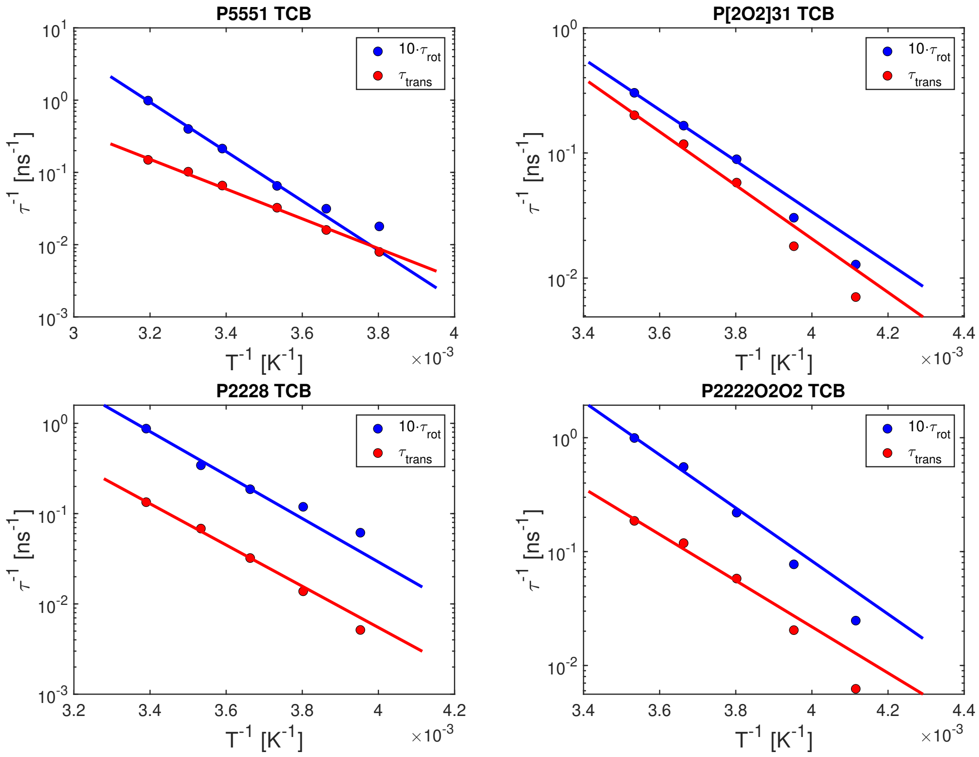

(c) $[P2228]^+$ (d) $[P2222O2O2]^+$

Figure S5: Arrhenius plots of the rotational and translation correlation times of the investigated $[B(CN)_4]$-ionic liquids

(a) $[P5551]^+$ (b) $[P(2O2)_3 1]^+$

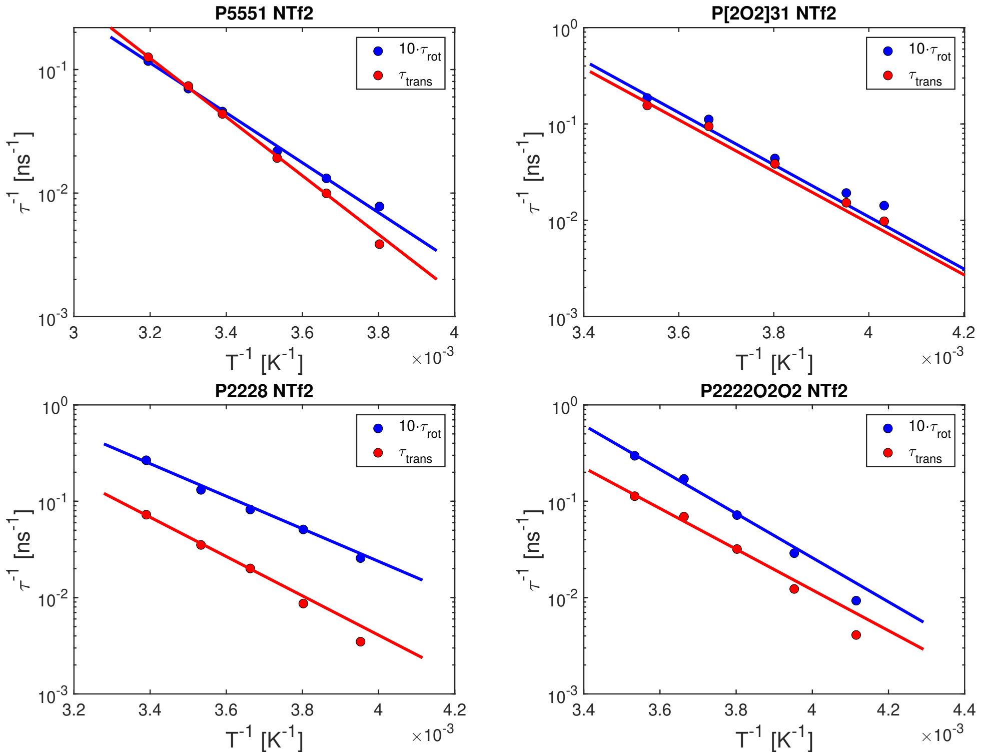

(c) $[P2228]^+$ (d) $[P2222O2O2]^+$

Figure S6: Arrhenius plots of the rotational and translation correlation times of the investigated $[N(Tf)_2]$-ionic liquids

Computational Details

Force Field Development

Force field parameters for the $[NTf_2]^-$ anion were taken from the literature unchanged.[17,18] For the $[B(CN)_4]^-$ anion, Lennard-Jones parameters for the nitrile[19] group and the boron atom[20,21] were taken from the literature. Parameters for B−C and C−N bond stretching (figure S8) as well as C−B−C and B−C−N angle bending (figure S7) were obtained from harmonic fits of the corresponding *ab initio* potential energy surface. The atomic charges for the $[B(CN)_4]^-$ anion were taken from the CHELPG or ADCH methods. CHELPG charges were used initially, in line with the $[NTf_2]^-$ anion.[22–24] As discussed in the main manuscript, the CHELPG charges proved inadequate, and instead ADCH charges were used successfully.[25]

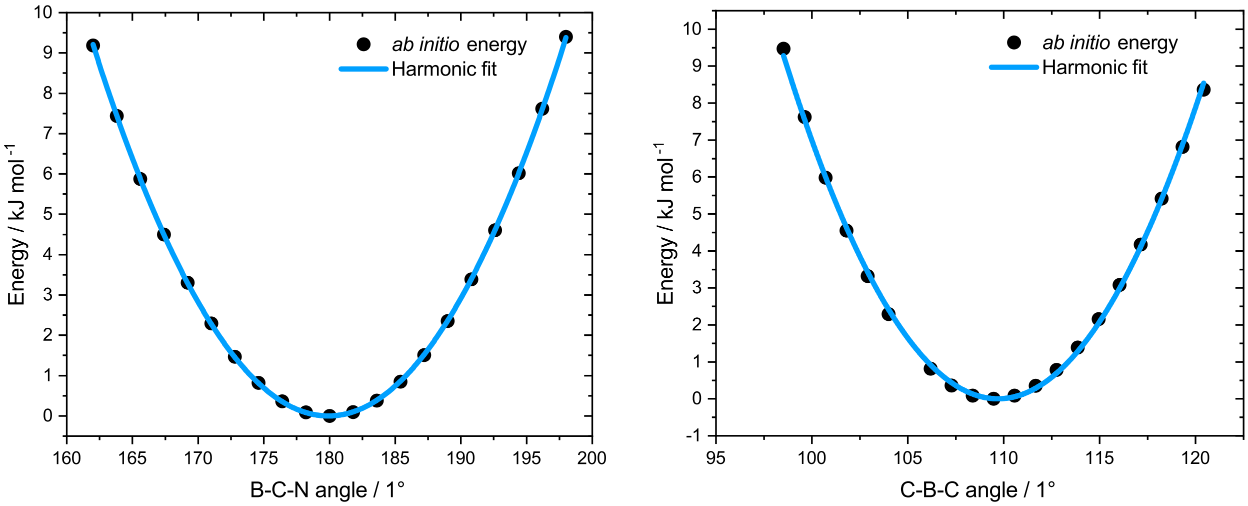

(a) B−C−N (b) C−B−C

Figure S7: Harmonic fits of the *ab initio* potential energy scans for (a) the B−C−N angle and (b) the C−B−C angle.

For all cations, ADCH atomic charges were used. Literature Lennard-Jones parameters were used for alkyl chains[17] and phosphorus[26]. Bond and angle stretching constants for all pairs and triples of atoms involving only C, O and H atoms were taken from the literature.[27,28] The P−C harmonic bond stretching parameters were obtained from the corresponding *ab*

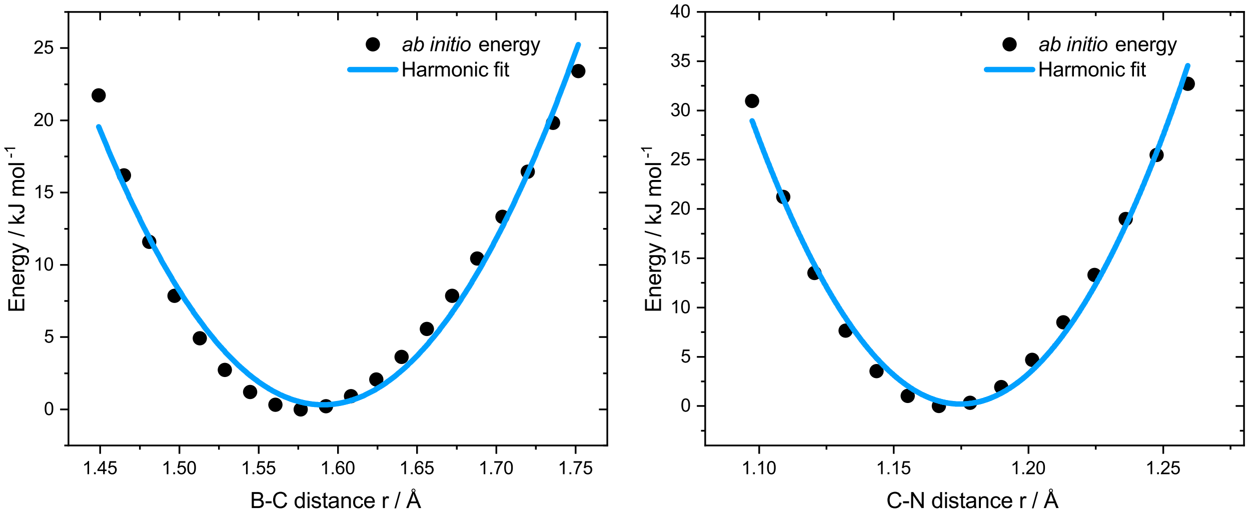

(a) B−C (b) C−N

Figure S8: Harmonic fits of the *ab initio* potential energy scans for (a) the B−C bond and (b) the C−N bond.

initio potential energy scan for $PMe_4{}^+$, figure S9. Dihedral parameters involving H atoms were taken from the literature.[26,28] All backbone dihedrals were determined from individual fits; this is with the exception of the $[P222(2O2O2)]^+$ and $[P2228]^+$ cation, for which the parameters were taken over from $[P(2O2)_3 1]^+$ and $[P5551]^+$.

In the main manuscript, we use artificially linearised (or curled) $[P(2O2)_3 1]^+$ (or $[P5551]^+$) cations as a targeted modification to evaluate the effect on macroscopic properties. To this end, we fit the truncated Fourier series used in the OPLS potential using the 'wrong' *ab initio* potential energy surface. Hence, we use the potential energy surface of the $[P1115]^+$ (or $[P111(2O2)]^+$) model cation to generate the target difference function to fit for the $[P(2O2)_3 1]^+$ (or $[P5551]^+$) cation. figure S10 shows the example for the $[P(2O2)_3 1]^+$ cation.

Ab initio scans required for the parameterization were performed at the full MP2/cc-pVTZ//B3LYP-GD3BJ/6-311+G(d,p) level of theory as described in the literature.[1]

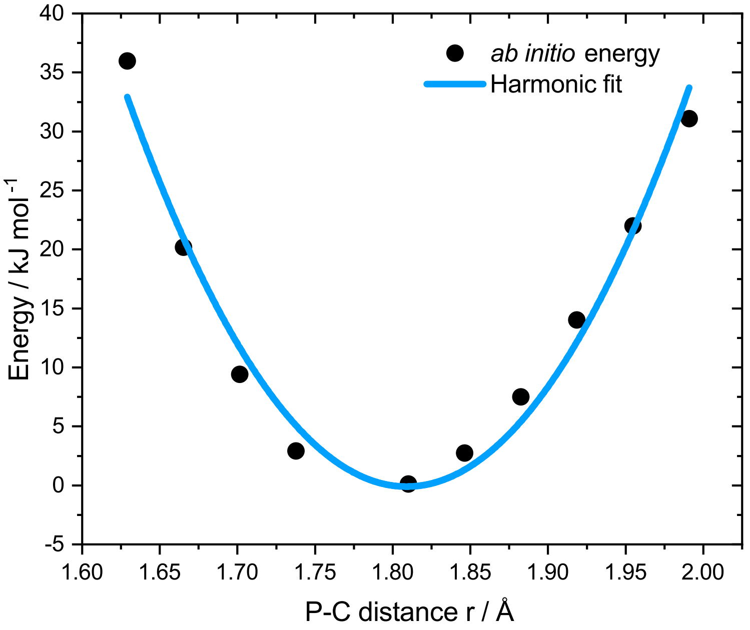

Figure S9: P−C bond stretching parameterization based on the tetramethylphosphonium cation (harmonic fit).

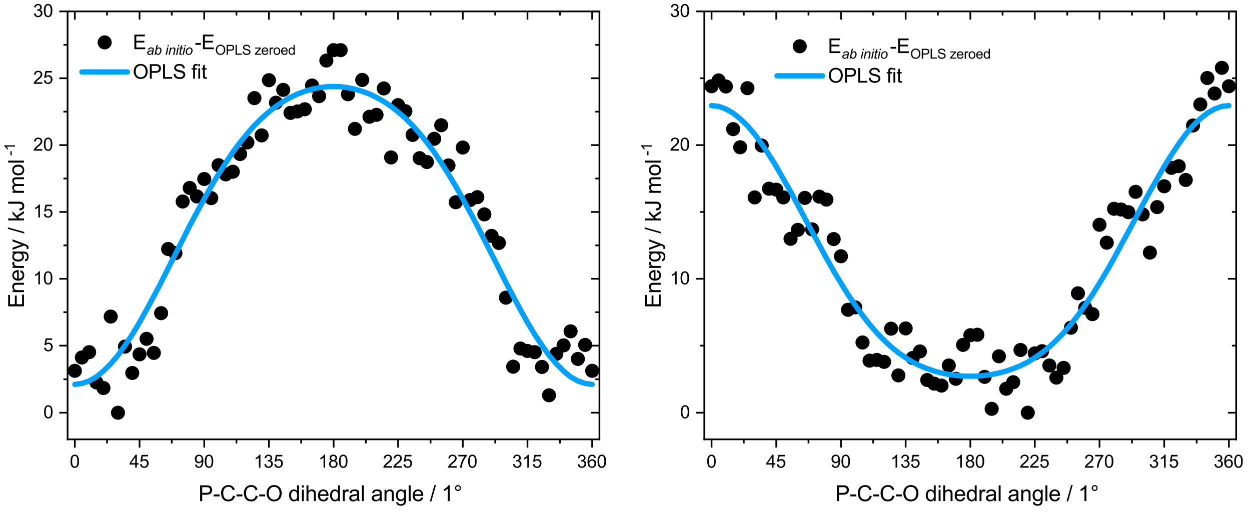

(a) curled (b) linear

Figure S10: The dihedral fit function leading to the (a) curled and (b) linear geometry.

Simulation Setup

Classical Molecular Dynamics simulations were run using the LAMMPS software package (version 11 Aug 2017).[29] polarizable simulations were run using the version 7 Aug 2019 compiled with the ttdamp branch to enable the use of a temperature grouped thermostat, which is now part of the official USER-DRUDE package.[30] Explicit polarizability was realised by means of Drude particles which were added to all non-Hydrogen atoms.[31,32] The Drude particles were assigned a mass of 0.4 Da and connected to their respective cores with a harmonic potential with a equilibrium distance of 0.0 Å and a spring constant of $k_{Drude} = 1000$ kcal mol^{-1} = 4184 kJ mol^{-1}.[33] The charges of drude particles were calculated as $q_{Drude} = \sqrt{\alpha k_{Drude}}$, where α are the atomic polarizabilities given in the literature.[34]

All simulations were run with periodic boundary conditions, solving Coulombic interactions with a particle-particle particle-mesh solver with a relative RMS force error of 10^{-5} for non-polarizable simulations and 10^{-6} for polarizable simulations. A cutoff of 12.0 Å was used for nonbonded (Coulomb and 12/6 Lennard-Jones) interactions together with a Van der Waals tail correction.[35] Short range dipole-dipole Coulombic interactions were damped *via* Thole screening using $\alpha = 2.6$.[31,36] Nonbonded interactions (atomic charges and LJ interactions, but not dipole-dipole interactions) were scaled with 0.0 for pairs of atoms separated by one or two bonds, and scaled with 0.5 for pairs of atoms separated by exactly three bonds. Geometric mixing was employed to obtain Lennard-Jones parameters between different atom types. To avoid double counting of induction effects, the Lennard-Jones ϵ parameters were scaled with the factors shown in table S14 evaluated from the predictive scheme, in line with the approach of the CL&Pol force field.

The SHAKE algorithm was used to fix all C−H bonds to their equilibrium value, with a tolerance of 10^{-4} and not more than 20 iterations.[37] Neighbour lists were used and rebuilt with a skin distance of 2.0 Å. The timestep for numerical integration was 1.0 fs for non-polarizable simulations and 0.5 fs for polarizable simulations. The momentum of the whole simulation box was subtracted every 1000 timesteps, for simulations in the microcanonical

Table S14: Scaling factors.

Fragment 1	Fragment 2	Scaling factor k
$[P5551]^+$	$[NTf_2]^-$	0.59
$[P(2O2)_31]^+$	$[NTf_2]^-$	0.55
$[P5551]^+$	$[B(CN)_4]^-$	0.62
$[P(2O2)_31]^+$	$[B(CN)_4]^-$	0.57
$[P(2O2)_31]^+$	$[P(2O2)_31]^+$	0.43 ($k_{++/--}$)
$[P5551]^+$	$[P5551]^+$	0.41 ($k_{++/--}$)
$[B(CN)_4]^-$	$[B(CN)_4]^-$	0.50 ($k_{++/--}$)

ensemble the velocities were rescaled to account for the resulting loss in kinetic energy.

Production runs for non-polarizable simulations were performed in the microcanonical (NVE) ensemble, integrating the equations of motion with the velocity-Verlet algorithm. Non-polarizable simulations were run at a target temperature of 400 K, whereas polarizable simulations were run at a target temperature of 298 K. The Drude particles (relative to their respective cores) were thermostatted at 1 K.[33] A Nosé-Hoover chain thermostat (and barostat) with a chain length of three and including the Martyna-Tuckerman-Klein correction was used for simulations in the NVT (NPT) ensemble.[38,39] Damping parameters were set to 1 ps for pressure control, 100 fs for temperature control, and 25 fs for temperature control of the internal motions of Drude-Core pairs. Furthermore, for polarizable simulations, a temperature grouped thermostat was used as described in the literature.[30,40]

Prior to the simulation, 512 ion pairs were packed into a cubic box and converted to a LAMMPS data file using fftool (`https://github.com/paduagroup/fftool`).[41] The polarizer and scaleLJ scripts were used to prepare input files for polarizable simulations. (`https://github.com/paduagroup/clandpol`) The preparation and equilibration procedure is described below.

The TRAVIS software package was used to generate radial and spatial distribution functions.[42,43] All other analyses were performed with the prealpha software package (source code, executable, input files and manual available on github, `https://github.com/FPhilippi/prealpha`). VMD was used for visualisation of spatial distribution functions.[44]

Preparation and Equilibration

After ion pairs were randomly packed into a cubic box, the energy of the system was minimised with a conjugate gradient algorithm (maximum of 100 iterations), and atoms were assigned random initial velocities corresponding to the target temperature. Pressure was kept at 1 bar for simulations in the NPT ensemble.

For the non-polarizable simulations, the following procedure was used. The system was kept at the target temperature of 400 K for 10 ps, heated to 600 K in a linear ramp over 10 ps, kept at 600 K for 500 ps, cooled to 400 K with a linear ramp over 10 ps, and kept at 400 K for 10 ps. This initial annealing procedure (540 ps) was performed in the NPT ensemble. In the following, the system was thermostatted to 400 K for 300 ps in NVT, and then for 500 ps in NPT. During the NPT run, the cell volume was recorded every 10 steps, and averaged over the 500 ps. Then this local average cell volume was appended to a file, and the simulation cell volume was compressed to the obtained local average over 100 ps. This 3 step sequence was repeated 20 times. Then, absence of drift in the cell volume was confirmed by visual inspection, and an overall average cell volume was obtained. The system was subsequently compressed to the overall average cell volume over 100 ps, and left to equilibrate in the NVT ensemble for 10 ns. Finally, this was followed by a 20 ns production run, during which the trajectory was written every 1 ps.

The general procedure for polarizable simulations was as follows. The system was kept at the target temperature of 298 K for 50 ps, heated to 600 K in a linear ramp over 50 ps, kept at 600 K for 100 ps, and cooled to 298 K with a linear ramp over 50 ps. This initial annealing procedure (250 ps) was performed in the NPT ensemble. In the following, the system was thermostatted to 298 K for 25 ps in NVT, and then for 150 ps in NPT. During the NPT run, the cell volume was recorded every 10 steps, and averaged over the 150 ps. Then this local average cell volume was appended to a file. This 2 step sequence was repeated 10-20 times, absence of drift in the cell volume was confirmed by visual inspection, and an overall average cell volume was obtained. The system was subsequently compressed to the overall

average cell volume over 25 ps, and left to equilibrate in the NVT ensemble for 4 ns. Finally, this was followed by a 20 ns production run, during which the trajectory was written every 1 ps. An additional short production run of 50 ps total duration was performed where the trajectory was written every 1 fs.

Validation

The density of the MD simulation can be obtained from the average of the cell vector a. Table S15 compares the simulation densities ρ_{MD} with the experimental densities $\rho_{exp.}$ from the literature.[1]

Table S15: Comparison of experimental and MD simulation densities. The second column indicates the charge fitting scheme used to parameterise the anion, and whether cation-cation and anion-anion Lennard-Jones interactions were scaled.

Ionic Liquid		a / Å	ρ_{MD} / g cm^{-3}	$\rho_{exp.}$ / g cm^{-3}	Dev.
$[P(2O2)_31][NTf_2]$	CHELPG	71.81(3)	1.253(1)	1.293(1)	-3%
$[P5551][NTf_2]$	CHELPG	73.42(3)	1.159(2)	1.202(1)	-4%
$[P(2O2)_31][B(CN)_4]$	CHELPG	68.12(1)	1.023(0)	0.995(1)	3%
$[P(2O2)_31][B(CN)_4]$	ADCH $k_{++/--}$	71.46(2)	0.886(1)	0.995(1)	-11%
$[P(2O2)_31][B(CN)_4]$	ADCH	68.69(3)	0.998(1)	0.995(1)	0.3%
$[P5551][B(CN)_4]$	CHELPG	70.18(1)	0.921(0)	0.907(1)	2%
$[P5551][B(CN)_4]$	ADCH $k_{++/--}$	73.53(3)	0.800(1)	0.907(1)	-12%
$[P5551][B(CN)_4]$	ADCH	70.54(2)	0.907(1)	0.907(1)	-0.03%

Experimental diffusion coefficients at 298 K are compared to those from the polarizable MD simulations in table S16 (Cation diffusion) and table S17 (Anion diffusion). The diffusion coefficients were obtained on a Bruker DMX300 spectrometer in combination with a Diff30 probe. For the diffusion coefficients of the cations an unaltered PGSTE sequence was used whereas for the diffusion coefficients of the TCB anion a hydrogen decoupled ^{13}C PG-STE pulse programme was employed.[45] Prior to the diffusion measurements a temperature calibration with ethylene glycol via the Merbach method was conducted.[46]

The rotational correlation times were obtained by fitting $C_2(t)$ according to equation S8.

Table S16: Comparison of cation self diffusion coefficients (at 298 K) from experiment and MD simulation.

Ionic Liquid		$10^{12}D^{+}_{self}$ / m^2s^{-1}		
		Experiment	MD simulation	Deviation
$[P(2O2)_31][NTf_2]$	CHELPG	26.2[1]	27.1(3)	3%
$[P5551][NTf_2]$	CHELPG	4.25[1]	8.0(4)	87%
$[P(2O2)_31][B(CN)_4]$	CHELPG	27.04	4.0(1)	-85%
$[P(2O2)_31][B(CN)_4]$	ADCH $k_{++/--}$	27.04	285(17)	954%
$[P(2O2)_31][B(CN)_4]$	ADCH	27.04	25(2)	-7%
$[P5551][B(CN)_4]$	CHELPG	6.357	2.1(1)	-67%
$[P5551][B(CN)_4]$	ADCH $k_{++/--}$	6.357	159(28)	2409%
$[P5551][B(CN)_4]$	ADCH	6.357	8.2(4)	28%

Table S17: Comparison of anion self diffusion coefficients (at 298 K) from experiment and MD simulation.

Ionic Liquid		$10^{12}D^{-}_{self}$ / m^2s^{-1}		
		Experiment	MD simulation	Deviation
$[P(2O2)_31][NTf_2]$	CHELPG	31.6[1]	32.8(5)	4%
$[P5551][NTf_2]$	CHELPG	5.33[1]	10.7(5)	101%
$[P(2O2)_31][B(CN)_4]$	CHELPG	37.72	4.4(3)	-88%
$[P(2O2)_31][B(CN)_4]$	ADCH $k_{++/--}$	37.72	297(25)	688%
$[P(2O2)_31][B(CN)_4]$	ADCH	37.72	33(3)	-12%
$[P5551][B(CN)_4]$	CHELPG	9.773	3.0(3)	-69%
$[P5551][B(CN)_4]$	ADCH $k_{++/--}$	9.773	166(13)	1597%
$[P5551][B(CN)_4]$	ADCH	9.773	11.0(2)	13%

Here, $A_3 = 1 - A_1 - A_2$, the integral gives equation S9 for the correlation time.

$$C_2(t) = A_1 e^{-t/\tau_1} + A_2 e^{-t/\tau_2} + A_3 e^{-t/\tau_3} \tag{S8}$$

$$\tau_{rot} = A_1\tau_1 + A_2\tau_2 + A_3\tau_3 \tag{S9}$$

The fit parameters are given in table S18 and table S19, the uncertainty was obtained from the standard errors of the fit using Gaussian error propagation.

For comparison with the molecular dynamics simulations, the FFC correlation times were extrapolated (or, where applicable, interpolated) using the Arrhenius fit to give the approximate experimental values in table S20.

Table S18: Fit parameters A_1 and A_2 for rotational correlation times.

Ionic Liquid		A_1	A_2
$[P(2O2)_31][NTf_2]$		0.0933(4)	0.2585(9)
$[P(2O2)_31][NTf_2]$	linear	0.2286(7)	0.1308(7)
$[P(2O2)_31][B(CN)_4]$		0.1947(6)	0.3998(15)
$[P(2O2)_31][B(CN)_4]$	linear	0.1418(4)	0.7017(3)
$[P5551][NTf_2]$		0.1710(8)	0.4329(9)
$[P5551][B(CN)_4]$		0.1831(6)	0.3593(6)
$[P(2O2)_31][B(CN)_4]$	ADCH	0.0974(4)	0.2706(10)
$[P(2O2)_31][B(CN)_4]$	ADCH $k_{++/--}$	0.1523(5)	0.2820(20)
$[P5551][B(CN)_4]$	ADCH	0.1443(8)	0.3517(9)
$[P5551][B(CN)_4]$	ADCH $k_{++/--}$	0.1117(6)	0.5200(19)

Table S19: Fit parameters τ_1, τ_2 and τ_3 for rotational correlation times.

Ionic Liquid		τ_1 / fs	τ_2 / fs	τ_3 / fs
$[P(2O2)_31][NTf_2]$		344(4)	47825(364)	403066(518)
$[P(2O2)_31][NTf_2]$	linear	442503(2513)	1241(12)	6516094(3604)
$[P(2O2)_31][B(CN)_4]$		1376(9)	729544(3775)	3035805(7530)
$[P(2O2)_31][B(CN)_4]$	linear	1434(11)	28388100(28766)	1548870(8852)
$[P5551][NTf_2]$		1615(13)	468084(1871)	3447897(5493)
$[P5551][B(CN)_4]$		1825(14)	1011990(3471)	10241600(11140)
$[P(2O2)_31][B(CN)_4]$	ADCH	353(4)	52033(401)	402896(581)
$[P(2O2)_31][B(CN)_4]$	ADCH $k_{++/--}$	518(2)	8552(90)	39569(102)
$[P5551][B(CN)_4]$	ADCH	1170(10)	277855(1386)	1991894(2563)
$[P5551][B(CN)_4]$	ADCH $k_{++/--}$	464(5)	122307(345)	20226(171)

Diffusion coefficients

Diffusion coefficients were obtained from the slope of a linear fit of the mean squared displacement using the Einstein relation. Data at short times must be discarded due to ballistic behavior, whereas data at long times suffer from poor statistics. Thus, the fits were performed for the mean squared displacements between 3 ns and 7 ns. The diffusion coefficients from the non-polarizable simulations (at 400 K simulation temperature) are given in table S21. Overall, the highest diffusion coefficients of both cation and anion were found for the ether functionalized cations and the $[B(CN)_4]^-$ anion, *i.e.* $[P(2O2)_31][B(CN)_4]$ and $[P222(2O2O2)][B(CN)_4]$.

Comparing ionic liquids which differ only in the presence of an ether oxygen (*i.e.* with

Table S20: Estimate for rotational and translational correlation times at 298 K, obtained from the Arrhenius fit of FFC data.

Ionic Liquid	τ_{Rot} / ns	τ_{Trans} / ns
[P5551][NTf_2]	1.82	19.0
[$P(2O2)_3 1$][NTf_2]	0.166	2.00
[P5551][$B(CN)_4$]	0.334	13.7
[$P(2O2)_3 1$][$B(CN)_4$]	0.140	2.06
[P2228][NTf_2]	0.343	11.9
[P222(2O2O2)][NTf_2]	0.129	3.62
[P2228][$B(CN)_4$]	0.102	6.16
[P222(2O2O2)][$B(CN)_4$]	0.0368	2.262

the same anion and the same cation backbone structure), it is evident that both cation and anions diffuse faster in those ionic liquids with an ether functionalised cation, in line with experimental observations. Within the group of ionic liquids with ether functionalised cations and identical anions, diffusion is faster for those with $[P(2O2)_3 1]^+$ cations rather than $[P222(2O2O2)]^+$ cations, presumably due to the additional ether functionalization. An opposite trend is observed for alkyl functionalised cations, where diffusion is observed to be slightly faster in ionic liquids with $[P2228]^+$ cations compared to those with $[P5551]^+$ cations. A possible explanation is the slightly higher molar mass of the latter.

Comparing pairs of ionic liquids with the same cation, it is evident that in all cases diffusion of both cation and anion is enhanced for $[B(CN)_4]^-$ based ionic liquids compared to $[NTf_2]^-$ based ionic liquids. This observation is in line with the experiments, although the difference is not very pronounced in most cases.

The $[P222(2O2O2)]^+$ and $[P2228]^+$ cations with one long side chain overall show the smallest ratios of cation to anion diffusion. This is likely a combination of the slightly lower mass compared to $[P(2O2)_3 1]^+$ and $[P5551]^+$ as well as the onset of interactions between the side chains (dipolar interactions between ether groups and Van der Waals interactions between alkyl side chains).

Table S21: Diffusion coefficients (at 400 K) from non-polarizable simulations.

Ionic Liquid		$10^{12}D^{+}_{self}$ / m^2s^{-1}	$10^{12}D^{-}_{self}$ / m^2s^{-1}	D^{+}_{self} / D^{-}_{self}
$[P(2O2)_31][NTf_2]$		83(7)	85(3)	0.97(9)
$[P(2O2)_31][NTf_2]$	linear	21(2)	21(1)	1.02(11)
$[P(2O2)_31][B(CN)_4]$		166(2)	192(6)	0.86(3)
$[P(2O2)_31][B(CN)_4]$	linear	53(4)	58(3)	0.91(8)
$[P5551][NTf_2]$		26(3)	27(2)	0.97(14)
$[P5551][NTf_2]$	curled	15(1)	16(1)	0.94(8)
$[P5551][B(CN)_4]$		63(8)	70(6)	0.90(14)
$[P222(2O2O2)][B(CN)_4]$		133(4)	154(16)	0.86(9)
$[P2228][B(CN)_4]$		67(3)	82(6)	0.81(7)
$[P222(2O2O2)][NTf_2]$		57(2)	65(3)	0.88(5)
$[P2228][NTf_2]$		28(2)	33(3)	0.84(8)
$[P2221][NTf_2]$		52(1)	50(3)	1.03(7)
$[P3331][NTf_2]$		50(3)	52(1)	0.96(7)
$[P4441][NTf_2]$		33(4)	33(3)	0.99(14)
$[P2221][NTf_2]$	heavy	37(2)	40(1)	0.93(5)
$[P3331][NTf_2]$	heavy	50(5)	48(3)	1.03(13)
$[P4441][NTf_2]$	heavy	33(2)	31(1)	1.05(6)

Rotational Correlation Times

Rotational correlation times, table S22, were obtained from $C_{rot}(t)$ as described above. These correlation times were obtained from the reorientation of the $P-CH_3$ vector, and are thus a measure for the rotation of the anion as a whole. In contrast, FFC also contains contributions from intramolecular motion (of the side chains). This aspect is related to the Octopus effect, *i.e.* the different relaxation behavior of polar and apolar domains.[47] Furthermore, the FFC experiments were necessarily run at different temperatures than the MD simulations. The rotational correlation times from the MD simulation were 237.7(5) ps for $[P(2O2)_31][NTf_2]$ and 1569(5) ps for $[P5551][NTf_2]$. The experimental correlation times were 165.5 ps and 1824 ps, respectively. Regarding the ionic liquids with $[B(CN)_4]^-$ anion, our initial simulation setup with CHELPG charges leads to simulated orientational correlation times which are much too long. For both $[P(2O2)_31][B(CN)_4]$ and $[P5551][B(CN)_4]$, experimental correlation times are between those obtained for the simulations with the ADCH

charges. The ADCH charges with the original CL&Pol approach - scaling only cation-anion Lennard-Jones interactions - lead to correlation times which are longer than the experimental ones. In contrast, the simulated correlation times are too short for the MD simulations with cation-cation and anion-anion Lennard-Jones scaling. Overall, relatively good agreement between FFC experiment and MD simulation is observed, despite the subtle difficulties in force field parameterization and the different theoretical frameworks of the two methods. From a technical point of view this is reassuring since the temperature grouped thermostat does not explicitly act on molecular rotations, but appears to nevertheless generate rotational dynamics whose trends follow those of the translational dynamics in comparison with experimental values.

Table S22: Rotational correlation times from the molecular dynamics simulation.

Ionic Liquid		τ_{rot} / ns
$[P(2O2)_31][NTf_2]$		0.2737(5)
$[P(2O2)_31][NTf_2]$	linear	4.276(7) [a)]
$[P(2O2)_31][B(CN)_4]$		1.523(6)
$[P(2O2)_31][B(CN)_4]$	linear	20.16(2) [a)]
$[P5551][NTf_2]$		1.569(5)
$[P5551][B(CN)_4]$		5.051(10) [a)]
$[P(2O2)_31][B(CN)_4]$	ADCH	0.269(1)
$[P(2O2)_31][B(CN)_4]$	ADCH + $k_{++/--}$	0.0249(1)
$[P5551][B(CN)_4]$	ADCH	1.102(3)
$[P5551][B(CN)_4]$	ADCH + $k_{++/--}$	0.0711(3)

[a)] Did not decorrelate fully.

As mentioned in the main manuscript, the tetracyanoborate anion showed subtleties in its parameterisation which require further attention in the future, and potentially complementary *ab initio* simulations. However, the $[NTf_2]^-$ anion is very well parameterised. Notwithstanding the different temperatures of simulation and experiment, Figure S11 demonstrates that the simulations agree well with our experiments.

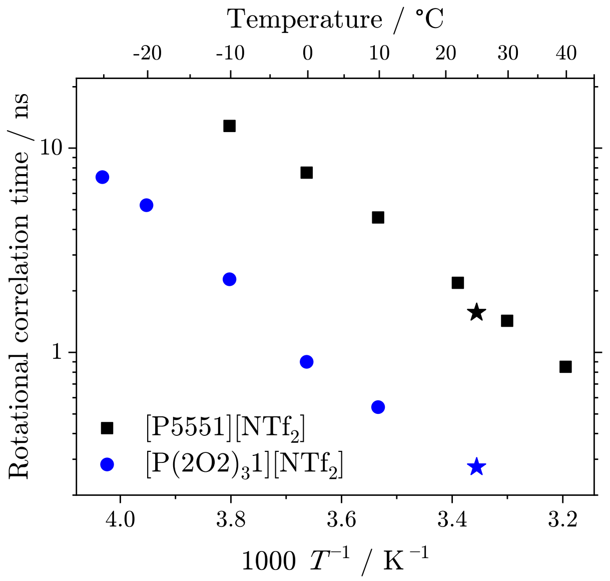

Figure S11: Rotational correlation times. The stars correspond to the correlation time from the MD simulation, squares and circles correspond to the experimental rotational correlation times.

Anion aggregation

The anion aggregation is visible in the anion-anion radial distribution functions, figure S12. The peak around 5.3 Å corresponds to anion-anion dimers. For $[P(2O2)_31][B(CN)_4]$ in the original CL&Pol force field with CHELPG anion atomic charges, the number integral at the position of the first minimum (7.1 Å) evaluates to 1.72. Thus, on average, every $[B(CN)_4]^-$ anion has almost two other anions as neighbour. This peak is still present for the force field with ADCH anion atomic charges, however it is much less pronounced, with a number integral of 0.97 at the position of the first minimum (7.0 Å). If in addition global Lennard-Jones scaling is introduced, then the dimer peak vanishes almost completely and only a weak shoulder is observed, closely following the non-polarizable simulation. This interesting situation warrants a more detailed study by means of *ab initio* simulations, which however is far beyond the scope of this work.

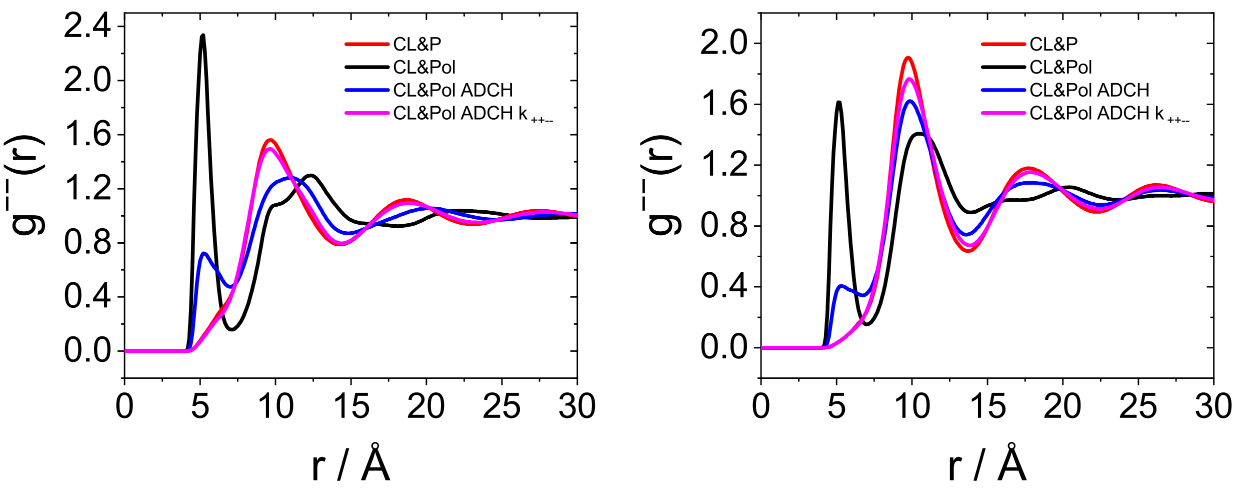

(a) $[P(2O2)_31][B(CN)_4]$ (b) $[P5551][B(CN)_4]$

Figure S12: anion-anion Radial distribution functions.

FFC distances

The exponentially weighed FFC distances are shown in table S23. These distances were computed using the prealpha software package, using equation S10 and S11.

$$b = \left(\frac{\sum_{i \neq j} e^{-r_{ij}} r_{ij}^{-6}}{\sum_{i \neq j} e^{-r_{ij}}} \right)^{-1/6} \tag{S10}$$

$$d = \left(\frac{\sum_{i \neq j} e^{-r_{ij}} r_{ij}^{-3}}{\sum_{i \neq j} e^{-r_{ij}}} \right)^{-1/3} \tag{S11}$$

Here, r_{ij} is the distance between the spins i and j. In the table, subscripts indicate the atom types of these two spins.

Atomic charges

figure S13 compares the atomic charges obtained with two different schemes, ADCH and CHELPG, for different cations in this work. For the accessible terminal hydrogen atoms, both charge schemes show good agreement, and the predicted charges are similar for the different cations, figure S13a. The same would be expected for the phosphorus atom in the phosphonium group, *i.e.* the atomic charge on this centre should not change significantly with

Table S23: Exponentially weighed FFC distances in Ångström.

Ionic Liquid	Force Field	d_{HH}	b_{HH}	d_{HF}	b_{FF}	d_{FF}
[P(2O2)$_3$1][NTf$_2$]	CL&Pol	3.470	2.016	3.525	2.141	3.844
[P(2O2)$_3$1][B(CN)$_4$]	CL&Pol	3.438	2.015			
[P5551][NTf$_2$]	CL&Pol	3.504	2.057	3.553	2.141	3.917
[P5551][NTf$_2$]	CL&Pol curled	3.523	2.063	3.556	2.141	3.923
[P5551][B(CN)$_4$]	CL&Pol	3.483	2.057			
[P(2O2)$_3$1][B(CN)$_4$]	CL&Pol, ADCH	3.486	2.015			
[P5551][B(CN)$_4$]	CL&Pol, ADCH	3.514	2.056			
[P(2O2)$_3$1][B(CN)$_4$]	CL&Pol, ADCH $k_{++/--}$	3.561	2.018			
[P5551][B(CN)$_4$]	CL&Pol, ADCH $k_{++/--}$	3.590	2.060			
[P2221][NTf$_2$]	CL&P, heavy	3.770	1.973	3.550	2.136	3.876
[P2221][NTf$_2$]	CL&P	3.758	1.972	3.549	2.135	3.876
[P2222O2O2][NTf$_2$]	CL&P	3.574	2.011	3.562	2.134	3.904
[P2222O2O2][B(CN)$_4$]	CL&P	3.590	2.012			
[P2228][NTf$_2$]	CL&P	3.571	2.039	3.576	2.134	3.994
[P2228][B(CN)$_4$]	CL&P	3.586	2.038			
[P(2O2)$_3$1][NTf$_2$]	CL&P	3.528	2.007	3.552	2.133	3.970
[P(2O2)$_3$1][NTf$_2$]	CL&P, linear	3.524	2.001	3.558	2.136	4.050
[P(2O2)$_3$1][B(CN)$_4$]	CL&P	3.550	2.007			
[P(2O2)$_3$1][B(CN)$_4$]	CL&P, linear	3.540	2.001			
[P3331][NTf$_2$]	CL&P, heavy	3.627	2.010	3.560	2.135	3.957
[P3331][NTf$_2$]	CL&P	3.638	2.010	3.561	2.135	3.928
[P4441][NTf$_2$]	CL&P, heavy	3.571	2.034	3.568	2.135	4.020
[P4441][NTf$_2$]	CL&P	3.577	2.034	3.563	2.135	3.992
[P5551][NTf$_2$]	CL&P	3.542	2.049	3.580	2.135	4.055
[P5551][B(CN)$_4$]	CL&P	3.556	2.049			

variations in the side chain. This is the case for the ADCH charges but not for the CHELPG charges, since the latter show serious stability issues with buried atoms, figure S13b.

Electrostatic potential

Figure S14 shows the electrostatic potential (ESP) evaluated at the full MP2/cc-pVTZ//B3LYP-GD3BJ/6-311+G(d,p) level of theory (using the MP2 density).[48,49] In all cases, the fully extended geometries were used with all-anti side chains to facilitate the interpretation of the graphs. The ESP was evaluated on the plane containing the backbone atoms of the longest side chain, with 100 grid points per dimension. The dashed lines correspond to the 0.001 a.u.

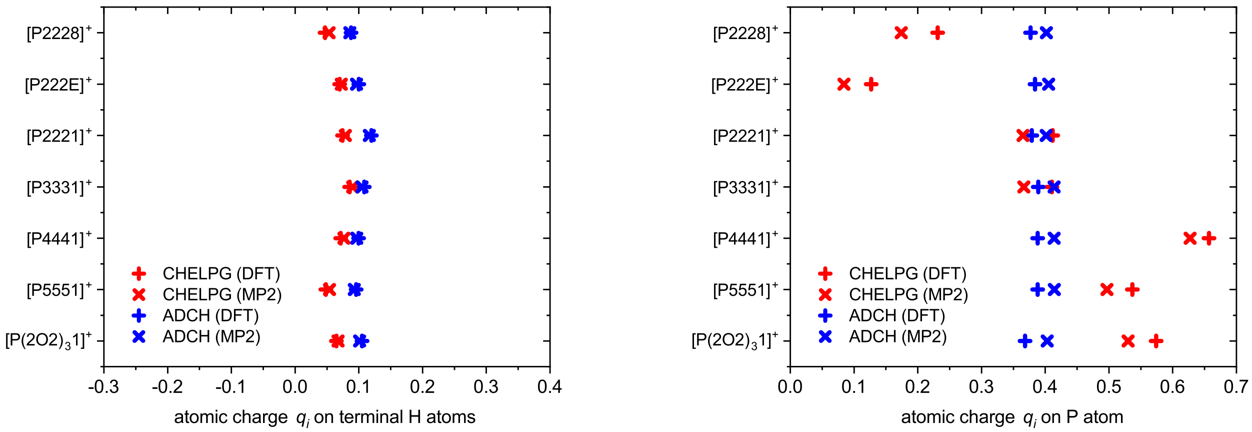

(a) Terminal Hydrogen Atoms

(b) Phosphonium centre

Figure S13: Atomic charges derived from CHELPG (red) and ADCH (blue). Hydrogen atoms (a) do not pose a problem for CHELPG, however the fitted charges become unstable for buried atoms, such as the P atom (b). Here, E=(2O2O2)

isosurface, *i.e.* the van der Waals surface.

(a) [P5551]$^+$ (b) [P(2O2)$_3$1]$^+$

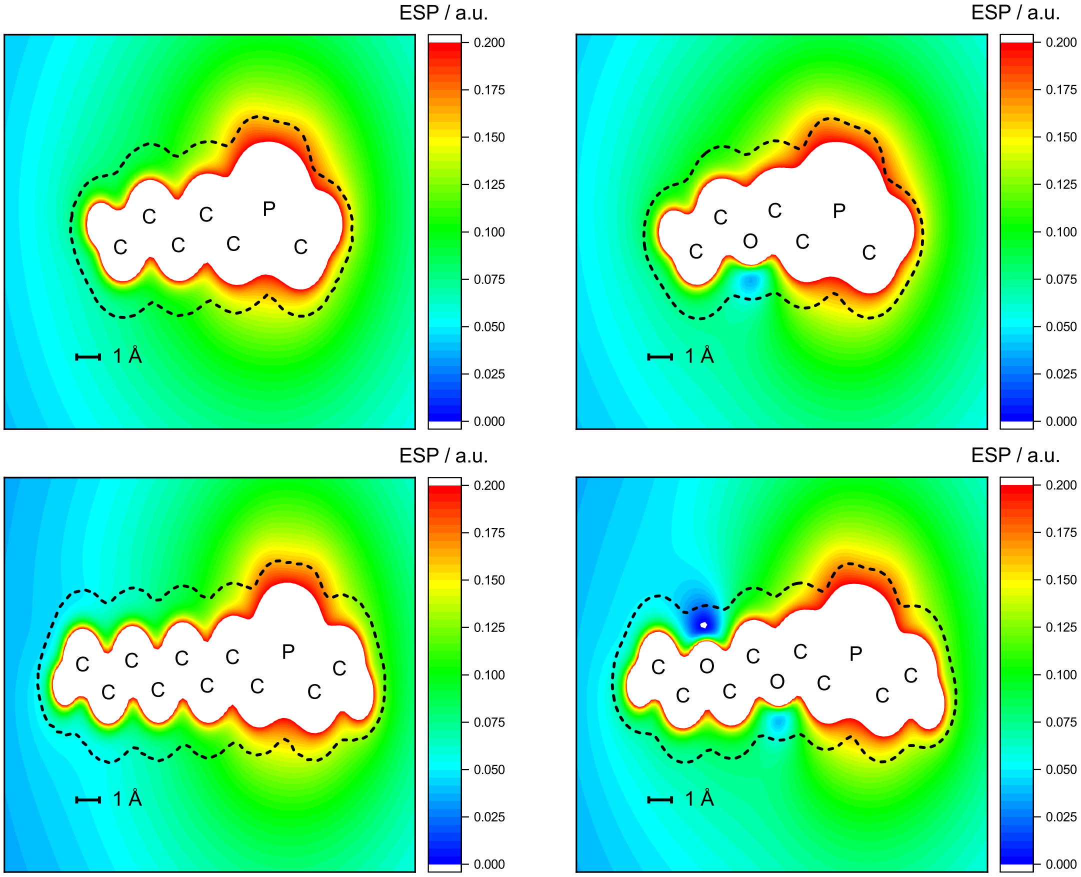

(c) [P2228]$^+$ (d) [P2222O2O2]$^+$

Figure S14: ESP plots of the investigated cations.

References

(1) Philippi, F.; Rauber, D.; Kuttich, B.; Kraus, T.; Kay, C. W. M.; Hempelmann, R.; Hunt, P. A.; Welton, T. Ether functionalisation, Ion Conformation and the Optimisation of Macroscopic Properties in Ionic Liquids. *Phys. Chem. Chem. Phys.* **2020**, *22*, 23038–23056.

(2) Philippi, F.; Rauber, D.; Zapp, J.; Präsang, C.; Scheschkewitz, D.; Hempelmann, R. Multiple Ether-Functionalized Phosphonium Ionic Liquids as Highly Fluid Electrolytes. *ChemPhysChem* **2019**, *20*, 443–455.

(3) Gómez, E.; Calvar, N.; Domínguez, Á. In *Ionic Liquids - Current State of the Art*; InTech: 2015, pp 199–208.

(4) Dean, P. M.; Pringle, J. M.; MacFarlane, D. R. Structural Analysis of Low Melting Organic Salts: Perspectives on Ionic Liquids. *Phys. Chem. Chem. Phys.* **2010**, *12*, 9144.

(5) Philippi, F.; Pugh, D.; Rauber, D.; Welton, T.; Hunt, P. A. Conformational Design Concepts for Anions in Ionic Liquids. *Chem. Sci.* **2020**, *11*, 6405–6422.

(6) Kashyap, H. K.; Santos, C. S.; Daly, R. P.; Hettige, J. J.; Murthy, N. S.; Shirota, H.; Castner, E. W.; Margulis, C. J. How Does the Ionic Liquid Organizational Landscape Change when Nonpolar Cationic Alkyl Groups Are Replaced by Polar Isoelectronic Diethers? *J. Phys. Chem. B* **2013**, *117*, 1130–1135.

(7) Sippel, P.; Lunkenheimer, P.; Krohns, S.; Thoms, E.; Loidl, A. Importance of Liquid Fragility for Energy Applications of Ionic Liquids. *Sci. Rep.* **2015**, *5*, 13922.

(8) Schreiner, C.; Zugmann, S.; Hartl, R.; Gores, H. J. Temperature Dependence of Viscosity and Specific Conductivity of Fluoroborate-Based Ionic Liquids in Light of the Fractional Walden Rule and Angell's Fragility Concept †. *J. Chem. Eng. Data* **2010**, *55*, 4372–4377.

(9) Harris, K. R.; Kanakubo, M.; Woolf, L. A. Temperature and Pressure Dependence of the Viscosity of the Ionic Liquid 1-Butyl-3-methylimidazolium Tetrafluoroborate: Viscosity and Density Relationships in Ionic Liquids. *J. Chem. Eng. Data* **2007**, *52*, 2425–2430.

(10) Harris, K. R.; Kanakubo, M.; Woolf, L. A. Temperature and Pressure Dependence of the Viscosity of the Ionic Liquids 1-Methyl-3-octylimidazolium Hexafluorophosphate and 1-Methyl-3-octylimidazolium Tetrafluoroborate. *J. Chem. Eng. Data* **2006**, *51*, 1161–1167.

(11) Harris, K. R.; Kanakubo, M.; Woolf, L. A. Temperature and Pressure Dependence of the Viscosity of the Ionic Liquids 1-Hexyl-3-methylimidazolium Hexafluorophosphate and 1-Butyl-3-methylimidazolium Bis(trifluoromethylsulfonyl)imide. *J. Chem. Eng. Data* **2007**, *52*, 1080–1085.

(12) Kelton, K. F. Kinetic and Structural Fragility—A Correlation between Structures and Dynamics in Metallic Liquids and Glasses. *J. Phys.: Condens. Matter* **2017**, *29*, 023002.

(13) Kruk, D.; Meier, R.; Rachocki, A.; Korpała, A.; Singh, R. K.; Rössler, E. A. Determining Diffusion Coefficients of Ionic Liquids by Means of Field Cycling Nuclear Magnetic Resonance Relaxometry. *J. Chem. Phys.* **2014**, *140*, 244509.

(14) Rainer Kimmich, *NMR-Tomography, Diffusometry, Relaxometry*; Springer Berlin, Heidelberg: 1997.

(15) Kimmich, R.; Anoardo, E. Field-Cycling NMR Relaxometry. *Prog. Nucl. Magn. Reson. Spectrosc.* **2004**, *44*, 257–320.

(16) Becher, M.; Horstmann, R.; Kloth, S.; Rössler, E. A.; Vogel, M. A Relation between the Formation of a Hydrogen-Bond Network and a Time-Scale Separation of Translation and Rotation in Molecular Liquids. *J. Phys. Chem. Lett.* **2022**, *13*, 4556–4562.

(17) Canongia Lopes, J. N.; Deschamps, J.; Padua, A. A. H. Modeling Ionic Liquids Using a Systematic All-Atom Force Field. *J. Phys. Chem. B* **2004**, *108*, 2038–2047.

(18) Gouveia, A. S. L.; Bernardes, C. E. S.; Tomé, L. C.; Lozinskaya, E. I.; Vygodskii, Y. S.; Shaplov, A. S.; Lopes, J. N. C.; Marrucho, I. M. Ionic liquids with Anions Based on Fluorosulfonyl Derivatives: From Asymmetrical Substitutions to a Consistent Force Field M odel. *Phys. Chem. Chem. Phys.* **2017**, *19*, 29617–29624.

(19) Price, M. L. P.; Ostrovsky, D.; Jorgensen, W. L. Gas-Phase and Liquid-State Properties of Esters, Nitriles, and Nitro Compounds with the OPLS-AA Force Field. *J. Comput. Chem.* **2001**, *22*, 1340–1352.

(20) Mayo, S. L.; Olafson, B. D.; Goddard, W. A. DREIDING: A Generic Force Field for Molecular Simulations. *J. Chem. Phys.* **1990**, *94*, 8897–8909.

(21) De Andrade, J.; Böes, E. S.; Stassen, H. Computational Study of Room Temperature Molten Salts Composed by 1-Alkyl-3-methylimidazolium CationsForce-Field Proposal and Validation. *J. Phys. Chem. B* **2002**, *106*, 13344–13351.

(22) Canongia Lopes, J. N.; Padua, A. A. H. CL&P: A Generic and Systematic Force Field for Ionic Liquids Modeling. *Theor. Chem. Acc.* **2012**, *131*, 1129.

(23) Breneman, C. M.; Wiberg, K. B. Determining Atom-Centered Monopoles from Molecular Electrostatic Potentials. The Need for High Sampling Density in Formamide Conformational Analysis. *J. Comput. Chem.* **1990**, *11*, 361–373.

(24) Chirlian, L. E.; Francl, M. M. Atomic Charges Derived from Electrostatic Potentials: A Detailed Study. *J. Comput. Chem.* **1987**, *8*, 894–905.

(25) Lu, T.; Chen, F. Atomic Dipole Moment Corrected Hirshfeld Population Method. *J. Theor. Comput. Chem.* **2012**, *11*, 163–183.

(26) Canongia Lopes, J. N.; Padua, A. A. H. Molecular Force Field for Ionic Liquids III: Imidazolium, Pyridinium, and Phosphonium Cations; Chloride, Bromide, and Dicyanamide Anions. *J. Phys. Chem. B* **2006**, *110*, 19586–19592.

(27) Kaminski, G.; Jorgensen, W. L. Performance of the AMBER94, MMFF94, and OPLS-AA Force Fields for Modeling Organic Liquids. *J. Chem. Phys.* **1996**, *100*, 18010–18013.

(28) Jorgensen, W. L.; Maxwell, D. S.; Tirado-Rives, J. Development and Testing of the OPLS All-Atom Force Field on Conformational Energetics and Properties of Organic Liquids. *J. Am. Chem. Soc.* **1996**, *118*, 11225–11236.

(29) Plimpton, S. Fast Parallel Algorithms for Short-Range Molecular Dynamics. *J. Comput. Phys.* **1995**, *117*, 1–19.

(30) Goloviznina, K.; Gong, Z.; Costa Gomes, M. F.; Pádua, A. A. H. Extension of the CL&Pol Polarizable Force Field to Electrolytes, Protic Ionic Liquids, and Deep Eutectic Solvents. *J. Chem. Theory Comput.* **2021**, *17*, 1606–1617.

(31) Dequidt, A.; Devémy, J.; Pádua, A. A. H. Thermalized Drude Oscillators with the LAMMPS Molecular Dynamics Simulator. *J. Chem. Inf. Model.* **2016**, *56*, 260–268.

(32) Goloviznina, K.; Canongia Lopes, J. N.; Costa Gomes, M.; Pádua, A. A. H. Transferable, Polarizable Force Field for Ionic Liquids. *J. Chem. Theory Comput.* **2019**, *15*, 5858–5871.

(33) Lamoureux, G.; Roux, B. Modeling Induced Polarization with Classical Drude Oscillators: Theory and Molecular Dynamics Simulation Algorithm. *J. Chem. Phys.* **2003**, *119*, 3025–3039.

(34) Heid, E.; Szabadi, A.; Schröder, C. Quantum Mechanical Determination of Atomic Polarizabilities of Ionic Liquids. *Phys. Chem. Chem. Phys.* **2018**, *20*, 10992–10996.

(35) Sun, H. COMPASS: An ab Initio Force-Field Optimized for Condensed-Phase ApplicationsOverview with Details on Alkane and Benzene Compounds. *J. Phys. Chem. B* **1998**, *102*, 7338–7364.

(36) Thole, B. Molecular Polarizabilities Calculated with a Modified Dipole Interaction. *Chem. Phys.* **1981**, *59*, 341–350.

(37) Ryckaert, J.-P.; Ciccotti, G.; Berendsen, H. J. Numerical Integration of the Cartesian Equations of Motion of a System with Constraints: Molecular Dynamics of n-Alkanes. *J. Comput. Phys.* **1977**, *23*, 327–341.

(38) Shinoda, W.; Shiga, M.; Mikami, M. Rapid Estimation of Elastic Constants by Molecular Dynamics Simulation Under Constant Stress. *Phys. Rev. B* **2004**, *69*, 134103.

(39) Martyna, G. J.; Tobias, D. J.; Klein, M. L. Constant Pressure Molecular Dynamics Algorithms. *J. Chem. Phys.* **1994**, *101*, 4177–4189.

(40) Son, C. Y.; McDaniel, J. G.; Cui, Q.; Yethiraj, A. Proper Thermal Equilibration of Simulations with Drude Polarizable Models: Temperature-Grouped Dual-Nosé–Hoover Thermostat. *J. Phys. Chem. Lett.* **2019**, *10*, 7523–7530.

(41) Martínez, L.; Andrade, R.; Birgin, E. G.; Martínez, J. M. PACKMOL: A Package for Building Initial Configurations for Molecular Dynamics Simulations. *J. Comput. Chem.* **2009**, *30*, 2157–2164.

(42) Brehm, M.; Kirchner, B. TRAVIS - A Free Analyzer and Visualizer for Monte Carlo and Molecular Dynamics Trajectories. *J. Chem. Inf. Model.* **2011**, *51*, 2007–2023.

(43) Brehm, M.; Thomas, M.; Gehrke, S.; Kirchner, B. TRAVIS—A Free Analyzer for Trajectories from Molecular Simulation. *J. Chem. Phys.* **2020**, *152*, 164105.

(44) Humphrey, W.; Dalke, A.; Schulten, K. VMD: Visual molecular dynamics. *J. Mol. Graph.* **1996**, *14*, 33–38.

(45) Tanner, J. E. Use of the Stimulated Echo in NMR Diffusion Studies. *J. Chem. Phys.* **1970**, *52*, 2523–2526.

(46) Ammann, C.; Meier, P.; Merbach, A. A Simple Multinuclear NMR Thermometer. *J. Magn. Reson.* **1982**, *46*, 319–321.

(47) Daly, R. P.; Araque, J. C.; Margulis, C. J. Communication: Stiff and Soft Nano-Environments and the “ Octopus Effect ” are the Crux of Ionic Liquid Structural and Dynamical Heterogeneity. *J. Chem. Phys.* **2017**, *147*, 061102.

(48) Zhang, J. libreta: Computerized Optimization and Code Synthesis for Electron Repulsion Integral Evaluation. *J. Chem. Theory Comput.* **2018**, *14*, 572–587.

(49) Lu, T.; Chen, F. Multiwfn: A Multifunctional Wavefunction Analyzer. *J. Comput. Chem.* **2012**, *33*, 580–592.
